# Supplementary material for: Loss of EHMT2 enhances NK cell-driven anti-tumor immunity through TGF-β1 suppression
Source: EMBO Mol Med. 2025 Dec 9;18(1):232–74. doi: 10.1038/s44321-025-00357-6 (PMC12808752; doi:10.1038/s44321-025-00357-6)
Supplement: Supplementary file 1 — Appendix [file 44321_2025_357_MOESM1_ESM.pdf]

Appendix

Appendix Figure S1. Role for EHMT1 in suppressing NK cell-mediated cytotoxicity in uveal melanoma. Page 2

Appendix Figure S2. Validation of *EHMT2* and *AZGP1* knockdown in UM cells. Page 3-4

Appendix Figure S3. Monitoring the cell surface expression of NKG2D ligands ULBP3 and MICB on UM cells under EHMT2 inhibition and *TGF-β1* knockdown using flow cytometry. Page 5-6

Appendix Figure S4. Monitoring the cell surface expression of NKG2D ligands ULBP3 and MICB under EHMT2 inhibition and *TGF-β1* overexpression using flow cytometry. Page 7

Appendix Figure S5. ULBP3 is partly necessary for EHMT2 inhibition-induced NK cell-mediated cytotoxicity. Page 8

Appendix Figure S6. TGF-β1 downregulates NK cell receptors expression. Page 9

Appendix Figure S7. Analysis of BRD4770 induced toxicity in mice. Page 10-11

Appendix Figure S8. Clonogenic assays for measuring the impact of pharmacological and genetic inhibition of EHMT2 on the indicated cancer cell lines. Page 12-13

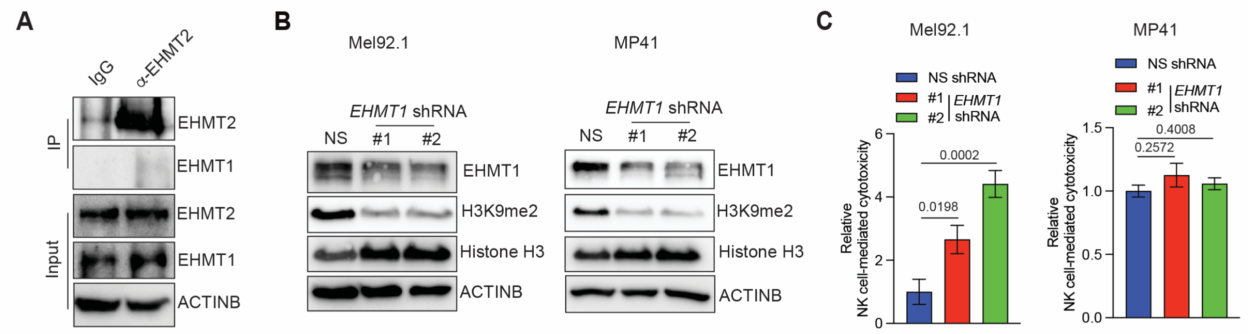

Appendix Figure S1. Role for EHMT1 in suppressing NK cell-mediated cytotoxicity in uveal melanoma. A. Lysates of Mel92.1 cells were immunoprecipitated using control IgG or anti-EHMT2 antibodies and analyzed by immunoblotting for the indicated proteins in immunoprecipitated (IP) and input samples. ACTINB was used as a loading control for input. **B.** Mel92.1 and MP41 cells expressing either non-specific (NS) shRNA or *EHMT1* shRNAs were analyzed for indicated proteins using immunoblotting. Histone H3 and ACTINB were used as loading controls. **C.** Mel92.1 and MP41 cells expressing either NS shRNA or *EHMT1* shRNAs were analyzed for NK cell-mediated cytotoxicity using LDH-based cytotoxicity assay. Relative NK cell-mediated cytotoxicity is plotted. (n=6). P-values were calculated using unpaired two-tailed Student's t-test. All quantitative data are shown as the mean ± SEM.

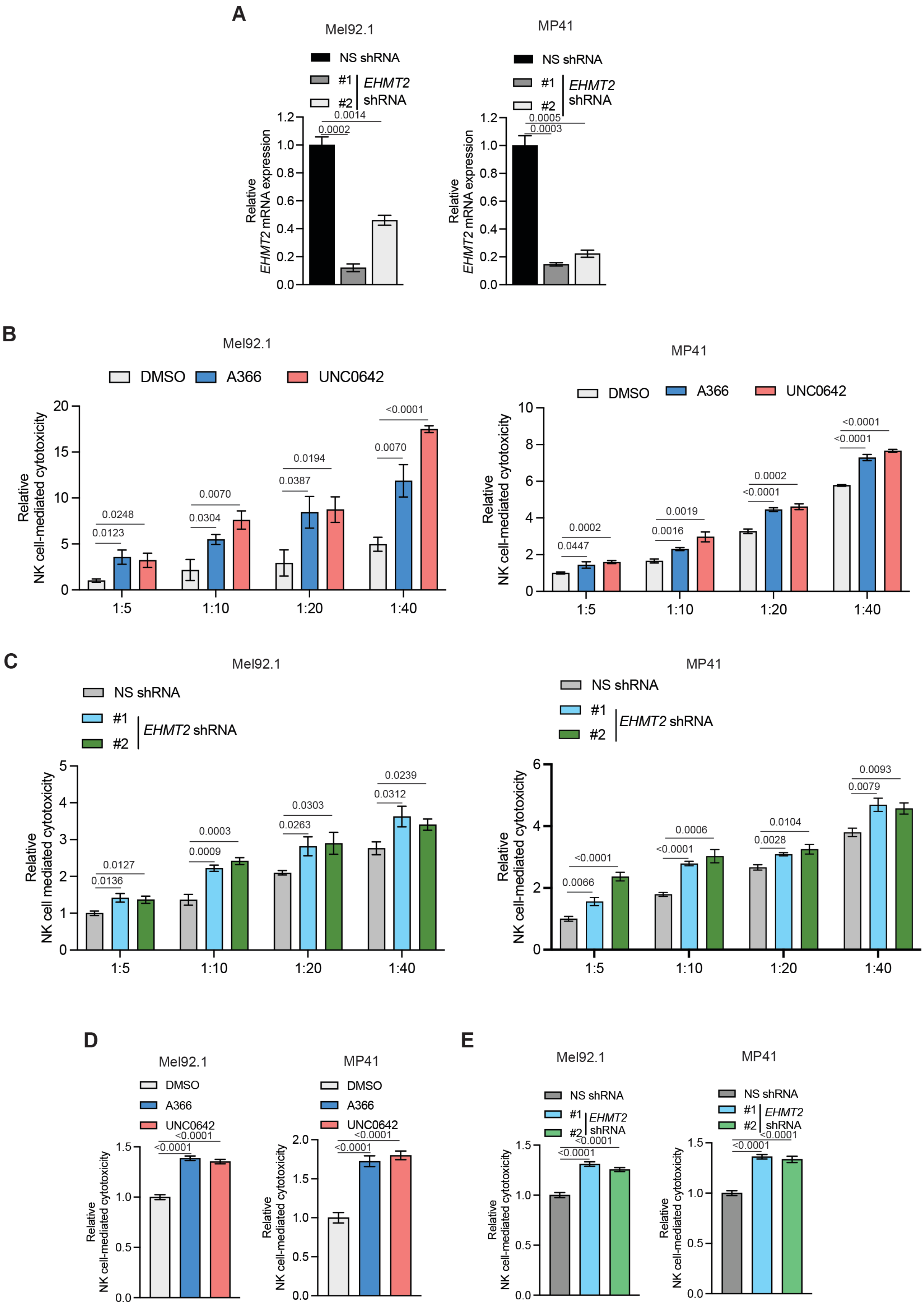

A
Mel92.1
MP41
NS shRNA
#1
#2
EHMT2 shRNA
Relative EHMT2 mRNA expression
0.0014
0.0002
0.0005
0.0003
B
Mel92.1
MP41
DMSO
A366
UNC0642
Relative NK cell-mediated cytotoxicity
0.0248
0.0123
0.0070
0.0304
0.0194
0.0387
<0.0001
0.0070
0.0002
0.0447
0.0019
0.0016
0.0002
<0.0001
<0.0001
<0.0001
1:5
1:10
1:20
1:40
C
Mel92.1
MP41
NS shRNA
#1
#2
EHMT2 shRNA
Relative NK cell mediated cytotoxicity
Relative NK cell-mediated cytotoxicity
0.0127
0.0136
0.0003
0.0009
0.0303
0.0263
0.0239
0.0312
<0.0001
0.0066
0.0006
<0.0001
0.0104
0.0028
0.0093
0.0079
1:5
1:10
1:20
1:40
D
Mel92.1
MP41
DMSO
A366
UNC0642
Relative NK cell-mediated cytotoxicity
<0.0001
<0.0001
E
Mel92.1
MP41
NS shRNA
#1
#2
EHMT2 shRNA
Relative NK cell-mediated cytotoxicity
<0.0001
<0.0001

Appendix Figure S2. Validation of *EHMT2* and *AZGP1* knockdown in UM cells. A. Mel92.1 and MP41 cells expressing either non-specific (NS) shRNA or *EHMT2* shRNAs were analyzed for mRNA expression for *EHMT2* mRNA were analyzed using RT-qPCR analysis. Relative mRNA expression for indicated genes are plotted. *ACTINB* was used for normalization. (n=3). P-values were calculated using unpaired two-tailed Student's t-test. **B.** Mel92.1 and MP41 cells were analyzed for NK cell-mediated cytotoxicity using LDH-based cytotoxicity assay at indicated cancer cell:NK cell ratios (1:5, 1:10, 1:20 or 1:40) after treatment with DMSO or indicated small molecule inhibitors of EHMT2 for 48 hr. NK cell-mediated cytotoxicity under indicated conditions is plotted relative to DMSO treatment at 1:5 cancer cell:NK cell ratio. (n=5). P-values were calculated using unpaired two-tailed Student's t-test. **C.** Mel92.1 and MP41 cells expressing either NS shRNA or *EHMT2* shRNA were analyzed for NK cell-mediated cytotoxicity using a LDH-based cytotoxicity assay at indicated cancer cell:NK cell ratios (1:5, 1:10, 1:20 or 1:40). Relative NK cell-mediated cytotoxicity for under indicated conditions is plotted. (n=5). P-values were calculated using unpaired two-tailed Student's t-test. **D.** Mel92.1 and MP41 cells were analyzed for NK cell-mediated cytotoxicity using a calcein-AM-based quantitative assay after treatment with DMSO or indicated inhibitors of EHMT2 for 48 hr. NK cell-mediated cytotoxicity under indicated conditions is plotted relative to DMSO treatment at 1:5 cancer cell:NK cell ratio. (n=5). P-values were calculated using unpaired two-tailed Student's t-test. **E.** Mel92.1 and MP41 cells expressing either NS shRNA or *EHMT2* shRNA were analyzed for NK cell-mediated cytotoxicity using a calcein-AM-based quantitative assay. Relative NK cell-mediated cytotoxicity for under indicated conditions is plotted. (n=5). P-values were calculated using unpaired two-tailed Student's t-test. All quantitative data are shown as the mean ± SEM.

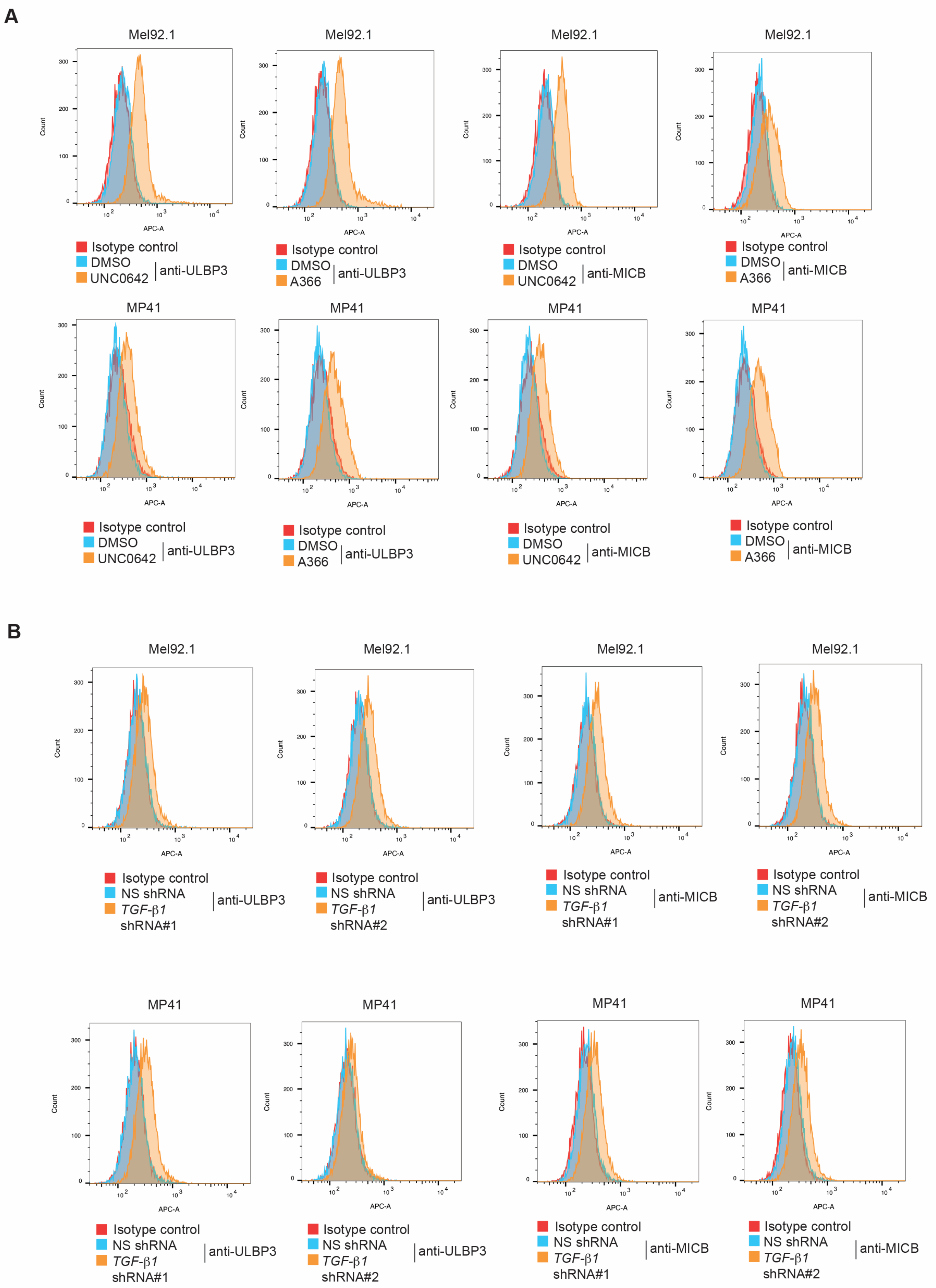
A
Mel92.1
Count
APC-A
Isotype control
DMSO
UNC0642
anti-ULBP3
Mel92.1
Isotype control
DMSO
A366
anti-ULBP3
Mel92.1
Isotype control
DMSO
UNC0642
anti-MICB
Mel92.1
Isotype control
DMSO
A366
anti-MICB
MP41
Isotype control
DMSO
UNC0642
anti-ULBP3
MP41
Isotype control
DMSO
A366
anti-ULBP3
MP41
Isotype control
DMSO
UNC0642
anti-MICB
MP41
Isotype control
DMSO
A366
anti-MICB
B
Mel92.1
Isotype control
NS shRNA
TGF-β1 shRNA#1
anti-ULBP3
Mel92.1
Isotype control
NS shRNA
TGF-β1 shRNA#2
anti-ULBP3
Mel92.1
Isotype control
NS shRNA
TGF-β1 shRNA#1
anti-MICB
Mel92.1
Isotype control
NS shRNA
TGF-β1 shRNA#2
anti-MICB
MP41
Isotype control
NS shRNA
TGF-β1 shRNA#1
anti-ULBP3
MP41
Isotype control
NS shRNA
TGF-β1 shRNA#2
anti-ULBP3
MP41
Isotype control
NS shRNA
TGF-β1 shRNA#1
anti-MICB
MP41
Isotype control
NS shRNA
TGF-β1 shRNA#2
anti-MICB

Appendix Figure S3. Monitoring the cell surface expression of NKG2D ligands ULBP3 and MICB on UM cells under EHMT2 inhibition and *TGF-β1* knockdown using flow cytometry. **A.** Mel92.1 and MP41 cells were treated with DMSO, UNC0642 (3 μM) or A366 (3 μM) for 72 hr and were analyzed for the cell surface expression of ULBP3 and MICB using flow cytometry, The representative histograms under the indicated conditions are shown. **B.** Mel92.1 and MP41 cells expressing non-specific (NS) shRNA or *TGF-β1* shRNA were analyzed for the cell surface expression of ULBP3 and MICB using flow cytometry. The representative histograms under the indicated conditions are shown.

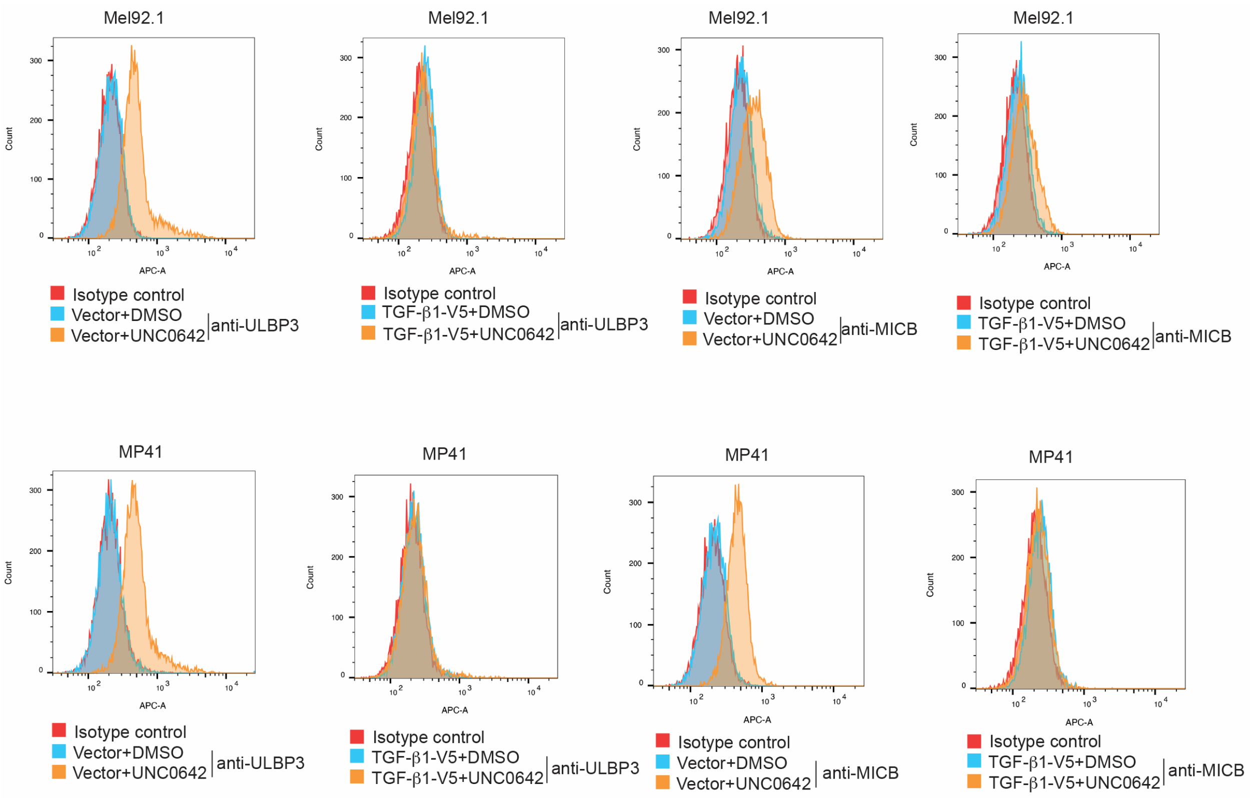

Appendix Figure S4. Monitoring the cell surface expression of NKG2D ligands ULBP3 and MICB under EHMT2 inhibition and *TGF-β1* overexpression using flow cytometry. Mel92.1 and MP41 cells expressing either an empty vector or V5-tagged *TGF-β1* ORF were treated with DMSO or UNC0642 (3 μM) for 72 hr and analyzed for the cell surface expression of ULBP3 and MICB using flow cytometry. The representative histograms under the indicated conditions are shown.

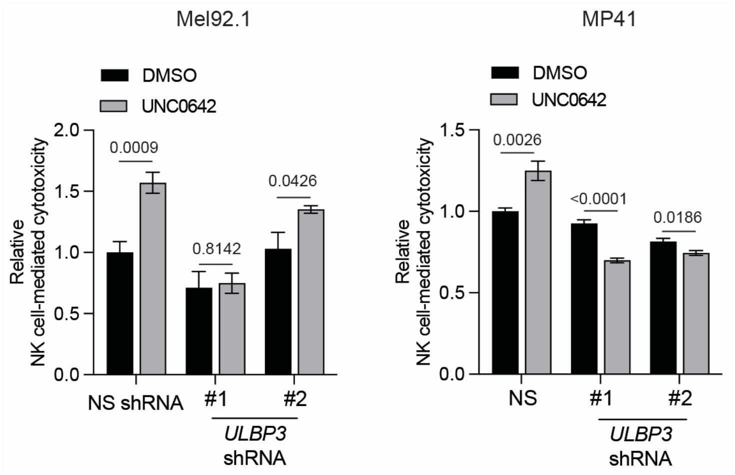

Appendix Figure S5. ULBP3 is partly necessary for EHMT2 inhibition-induced NK cell-mediated cytotoxicity. Mel92.1 and MP41 cells expressing non-specific (NS) shRNA or *ULBP3* shRNAs were treated with DMSO or EHMT2 inhibitor UNC0642 (1 μM) for 48 hr and were analyzed for NK cell-mediated cytotoxicity using LDH-based assay. Relative NK cells-mediated cytotoxicity is plotted under indicated conditions. (n=6). P-values were calculated using unpaired two-tailed Student's t-test. All quantitative data are shown as the mean ± SEM.

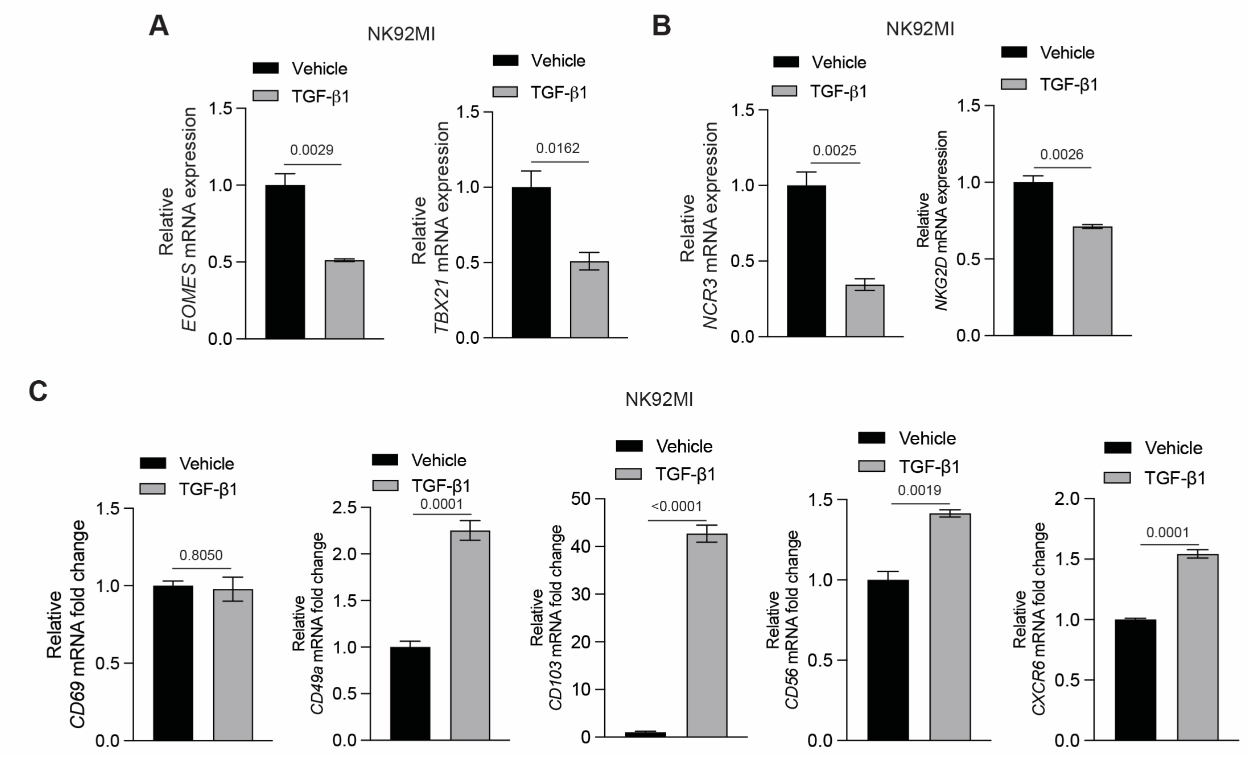

Appendix Figure S6. TGF-β1 downregulates NK cell receptors expression. A. NK92MI cells were treated with TGF- β1 (10 ng/ml) for 24 hr and analyzed for *EOMES* and *TBX21* mRNA levels using RT-qPCR. Relative mRNA levels are presented under indicated conditions. *ACTINB* was used for normalization. (n=3). P-values were calculated using unpaired two-tailed Student's t-test. **B.** NK92MI cells were treated with TGF- β1 (10 ng/ml) for 24 hr and analyzed for *NCR3* and *NKG2D* mRNA levels using RT-qPCR. Relative mRNA levels are presented under the indicated conditions. *ACTINB* was used for normalization. (n=3). P-values were calculated using unpaired two-tailed Student's t-test. **C.** NK92MI cells were treated with TGF-β1 (10 ng/ml) for 24 hr and analyzed for *CD69, CD49a CD103, CD56* and *CXCR6* mRNA levels using RT-qPCR. Relative mRNA levels are presented under the indicated conditions. *ACTINB* was used for normalization. (n=3). P-values were calculated using unpaired two-tailed Student's t-test. All quantitative data are shown as the mean ± SEM.

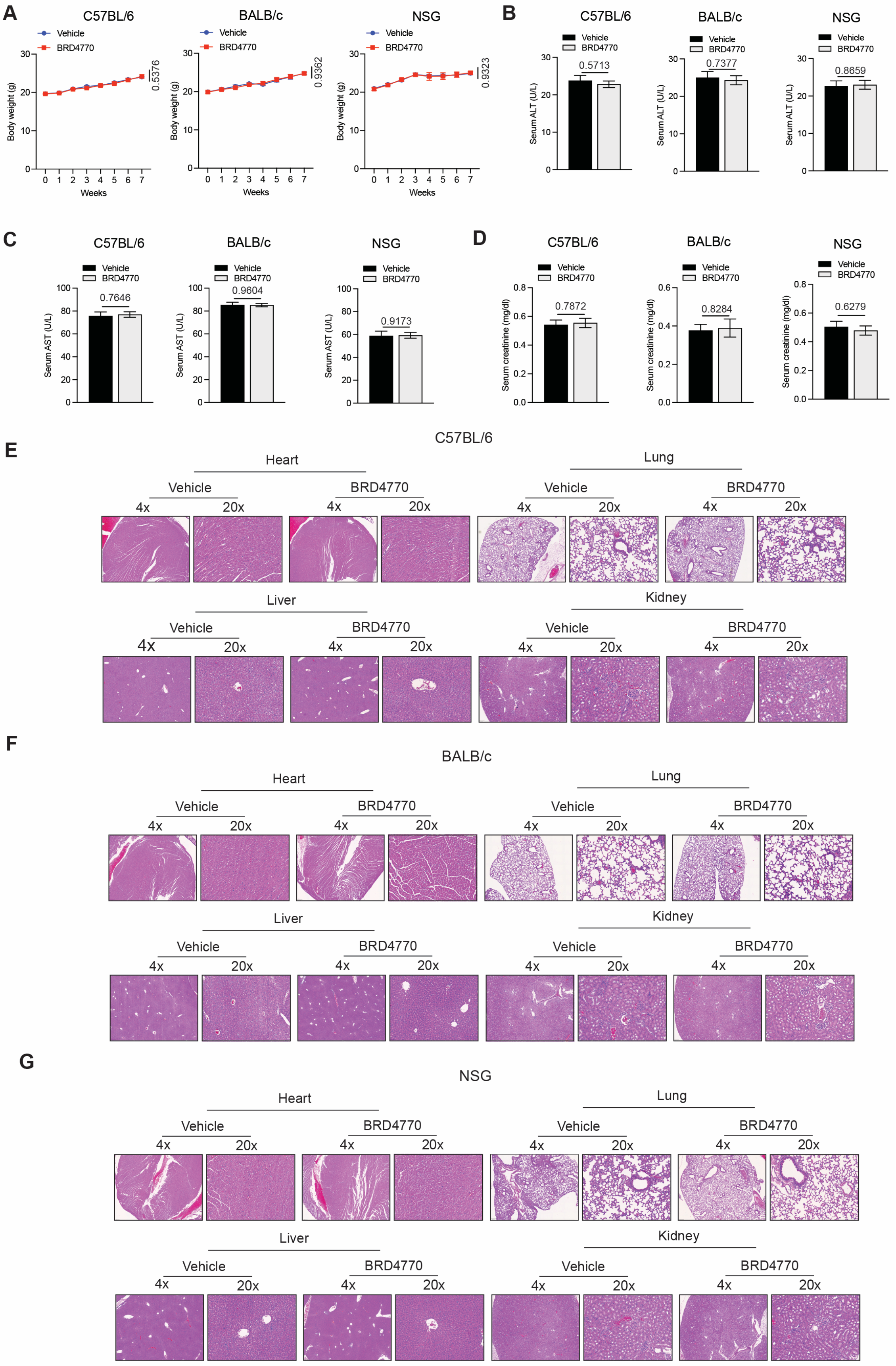

A
C57BL/6
BALB/c
NSG
Vehicle
BRD4770
Body weight (g)
Weeks
0.5376
0.9362
0.9323
B
Serum ALT (U/L)
0.5713
0.7377
0.8659
C
Serum AST (U/L)
0.7646
0.9604
0.9173
D
Serum creatinine (mg/dl)
0.7872
0.8284
0.6279
E
C57BL/6
Heart
Lung
Liver
Kidney
4x
20x
F
BALB/c
G
NSG

Appendix Figure S7. Analysis of BRD4770 induced toxicity in mice. **A.** C57BL/6, BALB/c and NSG mice were treated with vehicle or BRD4770 (15 mg/kg body weight) for 7 weeks. Body weights were measured at the indicated weeks. Average body weights are plotted at the indicated time points. (n=6). For the body weight measurements in mice, the statistical assessment was performed using the area under the curve method followed by unpaired two-tailed Student's *t*-tests. **B.** C57BL/6, BALB/c and NSG mice were treated with vehicle or BRD4770 (15 mg/kg body weight). Serum ALT levels were measured at the end of the experiment and is plotted under the indicated conditions. (n=6). P-values were calculated using unpaired two-tailed Student's t-test. **C.** C57BL/6, BALB/c and NSG mice were treated with vehicle or BRD4770 (15 mg/kg body weight). Serum AST levels were measured at the end of the experiment and is plotted under the indicated conditions. (n=6). P-values were calculated using unpaired two-tailed Student's t-test. **D.** C57BL/6, BALB/c and NSG mice were treated with vehicle or BRD4770 (15 mg/kg body weight). Serum creatinine levels were measured at the end of the experiment and is plotted under the indicated conditions. (n=6). P-values were calculated using unpaired two-tailed Student's t-test. **E.** Histopathological examination of vital organs (heart, lung, liver, and kidney) from C57BL/6 mice treated with vehicle or BRD4770 (15 mg/kg body weight) as shown by H&E staining images (Scale bar, 4×: 250 μm, 20×: 50 μm). **F.** Histopathological examination of vital organs (heart, lung, liver, and kidney) from BALB/c mice treated with vehicle or BRD4770 (15 mg/kg body weight) shown by H&E staining images (Scale bar, 4×: 250 μm, 20×: 50 μm). **G.** Histopathological examination of vital organs (heart, lung, liver, and kidney) from NSG mice treated with vehicle or BRD4770 (15 mg/kg body weight) shown by H&E staining images (Scale bar, 4×: 250 μm, 20×: 50 μm). All quantitative data are presented as the mean ± SEM.

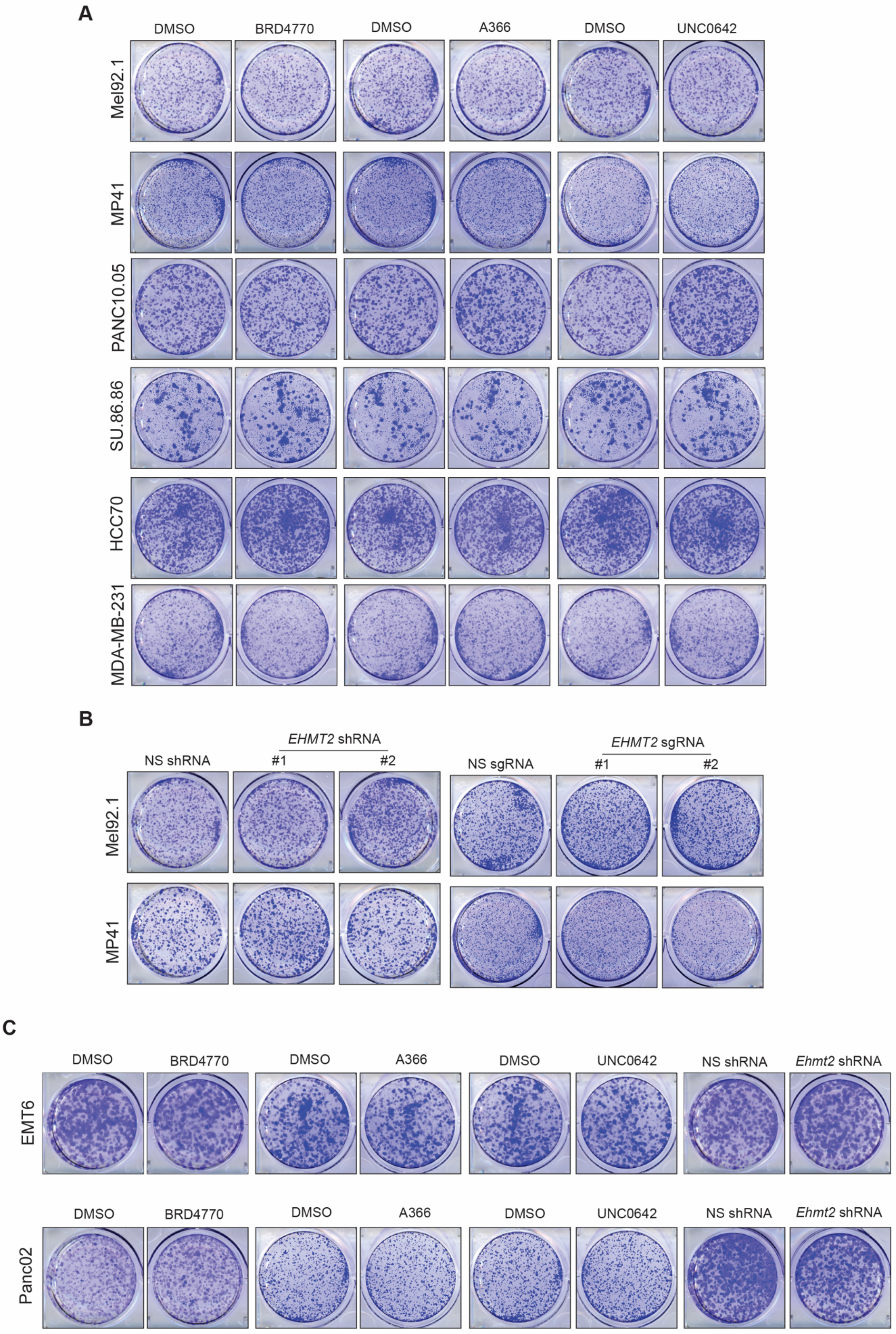
A
DMSO
BRD4770
DMSO
A366
DMSO
UNC0642
Mel92.1
MP41
PANC10.05
SU.86.86
HCC70
MDA-MB-231
B
NS shRNA
EHMT2 shRNA
#1
#2
NS sgRNA
EHMT2 sgRNA
#1
#2
Mel92.1
MP41
C
DMSO
BRD4770
DMSO
A366
DMSO
UNC0642
NS shRNA
Ehmt2 shRNA
EMT6
Panc02

Appendix Figure S8. Clonogenic assays for measuring the impact of pharmacological and genetic inhibition of EHMT2 on the indicated cancer cell lines. A. Indicated human cancer cell lines were treated with DMSO or EHMT2 inhibitors UNC0642 (1 μM) and A366 (1 μM) and were analyzed by clonogenic assay. Representative images of clonogenic assays for the indicated human cancer cell lines under the indicated conditions are shown. **B.** Indicated uveal melanoma cell lines expressing either non-specific (NS) shRNA or *EHMT2* shRNAs, or non-specific (NS) sgRNAs or *EHMT2*-targeting sgRNAs were analyzed by clonogenic assay. Representative images of clonogenic assays for the indicated uveal melanoma cell lines under the indicated conditions are shown. **C.** Indicated mouse cancer cell lines were treated with DMSO or indicated EHMT2 inhibitors BRD4770 (1 μM), UNC0642 (1 μM) and A366 (1 μM) or expressing NS shRNA or *Ehmt2* shRNA were analyzed by clonogenic assay. Representative images of clonogenic assays for the indicated mouse cancer cell lines under the indicated conditions are shown.
