# Supplementary material for: Loss of EHMT2 enhances NK cell-driven anti-tumor immunity through TGF-β1 suppression
Source: EMBO Mol Med. 2025 Dec 9;18(1):232–74. doi: 10.1038/s44321-025-00357-6 (PMC12808752; doi:10.1038/s44321-025-00357-6)
Supplement: Supplementary file 2 — Table EV1 [file 44321_2025_357_MOESM2_ESM.docx]

**Table EV1.** List of inhibitors targeting indicated chromatin modifiers and the concentrations at which they were used in the chemical genetic screen.

| **S.No.** | **Target protein** | **Inhibitors** | **Inhibitor**  **concentrations (µm)** |
| --- | --- | --- | --- |
| 1 | BAZ2A/2B | BAZ2-ICR | 1 |
| 2 | BAZ2A/2B | GSK2801 | 1 |
| 3 | BET family | JQ1 | 1 |
| 4 | BRD9/7 | BI-9564 | 1 |
| 5 | BRD9/7 | TP-472 | 1 |
| 6 | BRD9 | I-BRD9 | 1 |
| 7 | BRPF1/2/3; BRPF1B | NI-57 | 1 |
| 8 | BRPF1/2/3; BRPF1B | OF1 | 1 |
| 9 | BRPF1/2/3; BRPF1B | PFI-4 | 1 |
| 10 | CECR2 | NVS-CECR2-1 | 1 |
| 11 | CREBBP, EP300 | I-CBP112 | 1 |
| 12 | CREBBP, EP300 | SGC-CBP30 | 1 |
| 13 | DOT1L | SGC0946 | 1 |
| 14 | EED | A-395 | 1 |
| 15 | EZH2/H1 | GSK343 | 3 |
| 16 | G9a (EHMT2)/GLP | A-366 | 1 |
| 17 | G9a (EHMT2)/GLP | UNC0638 | 1 |
| 18 | G9a (EHMT2)/GLP | UNCO642 | 1 |
| 19 | IDH1 mutant | GSK864 | 1 |
| 20 | JMJD3/UTX (KDM6A/B) | GSK-J4 | 5 |
| 21 | LSD1 (KDM1A) | GSK-LSD1 | 1 |
| 22 | PAD4 (PADI4) | GSK484 | 10 |
| 23 | PRMT Type I | MS023 | 1 |
| 24 | PRMT3 | SGC707 | 1 |
| 25 | PRMT4 | TP-064 | 1 |
| 26 | PRMT4/6 | MS049 | 5 |
| 27 | PRMT5 | GSK591 | 1 |
| 28 | SETD7 | (R)-PFI-2 | 1 |
| 29 | SMARCA2/4, PB1 | PFI-3 | 1 |
| 30 | SMYD2 | BAY-598 | 1 |
| 31 | SUV420H1/H2 (KMT5B/C) | A-196 | 1 |
| 32 | WDR5 | OICR-9429 | 3 |
