# Supplementary material for: Loss of EHMT2 enhances NK cell-driven anti-tumor immunity through TGF-β1 suppression
Source: EMBO Mol Med. 2025 Dec 9;18(1):232–74. doi: 10.1038/s44321-025-00357-6 (PMC12808752; doi:10.1038/s44321-025-00357-6)
Supplement: Supplementary file 3 — Table EV2 [file 44321_2025_357_MOESM3_ESM.docx]

**Table EV2.** Oligonucleotides, and other sequence-based reagents

| Reagents and tools table | | |
| --- | --- | --- |
| Human primers | Forward primer | Reverse primer |
| *EHMT2* | CACCCTCTCTCCCCAGCATG | GTGTGAAAGGGTGGTGGGGA |
| *AZGP1* | AGGAGTGCCCTGCGACTCTG | ACTCAGGCTCCTGCACCTCG |
| *NOX5* | GGGCAGGAGGGAACAGCAGA | TGCAAGGTGGAGGATGCCCA |
| *BANCR* | GCAGCACCAATGGGCCAACT | AGTCGCCACTCCACTCAGCA |
| *SLAMF7* | TTCCCAGAGGGCCAGGTGTG | TGAAGCCTGGCTGGAGGTCC |
| *SERPINA1* | GCCTGTTGCTGTCCCTGCAG | AGGGGACTTCCTGGCTGCTG |
| *BST2* | GAGCTGACCGAGGCCCAGAA | CTGCAGACGCGTCCTGAAGC |
| *LINC00426* | GTTTCACTCGTCGCCCAGCC | TCTCACTCTGTCGCCCAGGC |
| *KRT23* | GTCCGGGGCTGCTAACAACG | CCCACGACATCAGGCGGGTA |
| *TGFB1* | CATTTGGAGCCTGGACACGC | TAGTACACGATGGGCAGCGG |
| *ULBP1* | CCACCAGGACTGGCAAACTG | ATTGGGAGGCCAAGGTGGTA |
| *ULBP2* | CAGGCACAACCCAACTCAGG | GCCAGACAGAAGGGCGAGTT |
| *ULBP3* | CCTCGCGATTCTTCCGTACC | GCCCCACCTCTCTCAGCAT |
| *ULBP4* | TCGCCACCAATGGAGAGAAA | ATTGCCTCCCAGTGCCCTAA |
| *ULBP5* | GCTTCTGCTCCTGCTGTCCA | GGGACTGACGGGTGTGACTG |
| *ULBP6* | GCCATGTCCTCAGGCACAAC | TCAGATGCCAGGGAGGATGA |
| *MICA* | CCTGCAATCCCAGCACTTTG | ATTCACCACCAAGCCCGTCT |
| *MICB* | CACGTTCGCCCTTTGTTCAG | GGAGGCAGAGGTTGCAGTGA |
| *B7H6* | CTGGATCAAGTGGGCATGAA | CTTGATGGTGGGACCAGTGA |
| *BAG6* | CCGGTTGCTGGAGTTGTGTA | CCCACGAGACATACGACGAA |
| *GALECTIN 3* | CCAGCAGTTCCCTAGGTTGG | AAAATTAGCCGGGTGTGGTG |
| *MLL5* | TCCTGGGTAAAGAGCCCTGA | TTATTCGGAAGCCTCCTCCA |
| *CCL11* | CCCCTTCAGCGACTAGAGAG | TCTTGGGGTCGGCACAGAT |
| *CXCL5* | AGCTGCGTTGCGTTTGTTTAC | TGGCGAACACTTGCAGATTAC |
| *CCL24* | ACATCATCCCTACGGGCTCT | CTTGGGGTCGCCACAGAAC |
| *CCL23* | CATCTCCTACACCCCACGAAG | GGGTTGGCACAGAAACGTC |
| *CCL27* | GCAGCATTCCTACTGCCAC | AGGTGAAGCACGAAAGCCTG |
| *CXCL10* | GTGGCATTCAAGGAGTACCTC | TGATGGCCTTCGATTCTGGATT |
| *CXCL7* | GTAACAGTGCGAGACCACTTC | CTTTGCCTTTCGCCAAGTTTC |
| *CXCL8* | TTTTGCCAAGGAGTGCTAAAGA | AACCCTCTGCACCCAGTTTTC |
| *CXCL3* | CCGTGGTCACTGAACTGCGC | ACTTCGGTTTGGGCGCAGTG |
| *CXCL1* | GTCCGTGGCCACTGAACTGC | ACTTCGGTTTGGGCGCAGTG |
| *CXCL2* | CCACTGAACTGCGCTGCCAG | ACTTCGGTTTGGGCGCAGTG |
| *CCL1* | CTCATTTGCGGAGCAAGAGAT | CTCATTTGCGGAGCAAGAGAT |
| *CCL13* | CTCAACGTCCCATCTACTTGC | TCTTCAGGGTGTGAGCTTTCC |
| *CCL15* | TCCCAGGCCCAGTTCATAAAT | TGCTTTGTGAGATGTAGGAGGT |
| *CCL16* | ACAGAAAGGCCCTCAACTGTC | TCCTTGATGTACTCTTGGACCC |
| *CCL28* | TGCACGGAGGTTTCACATCAT | TTGGCAGCTTGCACTTTCATC |
| *CCL4* | CTGTGCTGATCCCAGTGAATC | TCAGTTCAGTTCCAGGTCATACA |
| *CXCL13* | GCTTGAGGTGTAGATGTGTCC | CCCACGGGGCAAGATTTGAA |
| *CXCL16* | CCCGCCATCGGTTCAGTTC | CCCCGAGTAAGCATGTCCAC |
| *CXCL6* | AGAGCTGCGTTGCACTTGTT | GCAGTTTACCAATCGTTTTGGGG |
| *Lymphotactin* | TGCTCTCTCACTGCATACATTG | TGGTGTAGGTCTTGATTCTGCT |
| *EOMES* | TCCGAGCGGTACTACCTCCA | TAGGAGCCGGGTACACAGGT |
| *TBX21* | TATGCGGACTCTGCCCATGG | GAGTCTCCTTCGCCCAGTCC |
| *NCR3* | GGACAGGGAATGGGACTCGG | CCCACGGCCACAGAGAGAAA |
| *IFN-γ* | AGGCAGCCAACCTAAGCAAGA | ACTGGCTCAGATTGCAGGCA |
| *CD103* | TGCTGGCCGCTTTCAATGT | ACAGGATGGCAAAGGATTTCAT |
| *CD69* | ATTGTCCAGGCCAATACACATT | CCTCTCTACCTGCGTATCGTTTT |
| *CD49a* | CTGGACATAGTCATAGTGCTGGA | ACCTGTGTCTGTTTAGGACCA |
| *CD56* | GGCATTTACAAGTGTGTGGTTAC | TTGGCGCATTCTTGAACATGA |
| *CXCR6* | GACTATGGGTTCAGCAGTTTCA | GGCTCTGCAACTTATGGTAGAAG |
| *NKG2D* | GAGTGATTTTTCAACACGATGGC | ACAGTAACTTTCGGTCAAGGGAA |
| *ACTINB* | GTCTTCCCCTCCATCGTGGG | CCTCTCTTGCTCTGGGCCTC |
| *EHMT2* sgRNA#1 | CAAGAGGTGACCATCCCCCG | CGGGGGATGGTCACCTCTTG |
| *EHMT2* sgRNA#2 | CGGACAGGTACAACTGCCGA | TCGGCAGTTGTACCTGTCCG |
| Mouse primers | | |
| *Ehmt2* | AGCGGATACCTCCCAGCCTA | GAAGGCCCTGAGCTGTCGAT |
| *Actinb* | TTGCAGCTCCTTCGTTGCCG | GGAATACAGCCCGGGGAGCA |
| CUT&RUN PRIMERS | | |
| *AZGP1* | GGGCAGATCACGAGGTCAGG | CTGAGGCAGGGGTTTCACCA |
| *ACTINB* | TCTTGGCTGGGCGTGACTGT | AAGGTGGGCTCTACAGGGCA |
