# Supplementary figures and images for: Loss of EHMT2 enhances NK cell-driven anti-tumor immunity through TGF-β1 suppression
Source: EMBO Mol Med. 2025 Dec 9;18(1):232–74. doi: 10.1038/s44321-025-00357-6 (PMC12808752; doi:10.1038/s44321-025-00357-6)

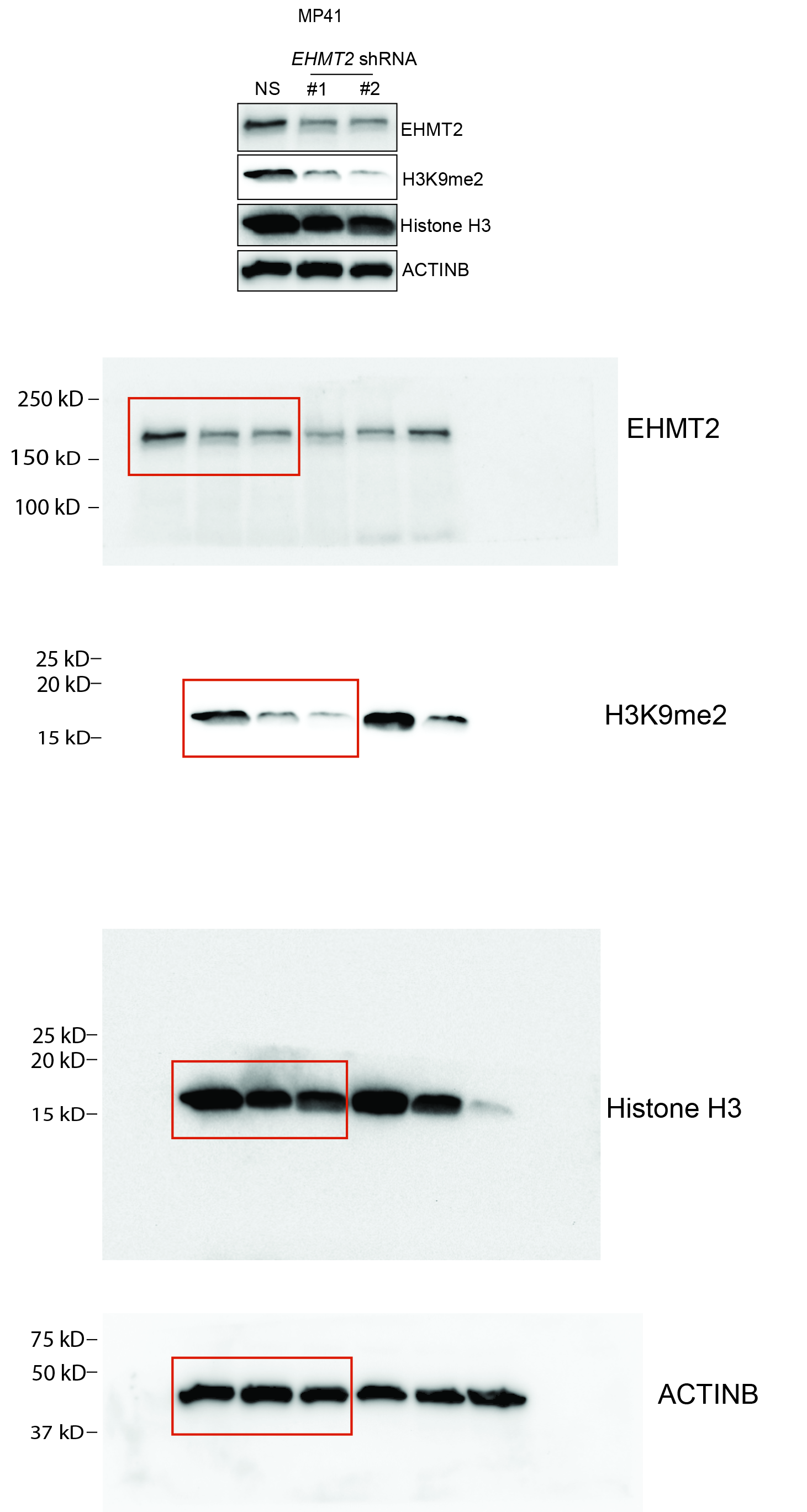

Supplement: Supplementary file 6 — Source data Fig. 1 [file 44321_2025_357_MOESM6_ESM.zip › Figure 1/1F/FIgure 1F-MP41 Western blots.tif]

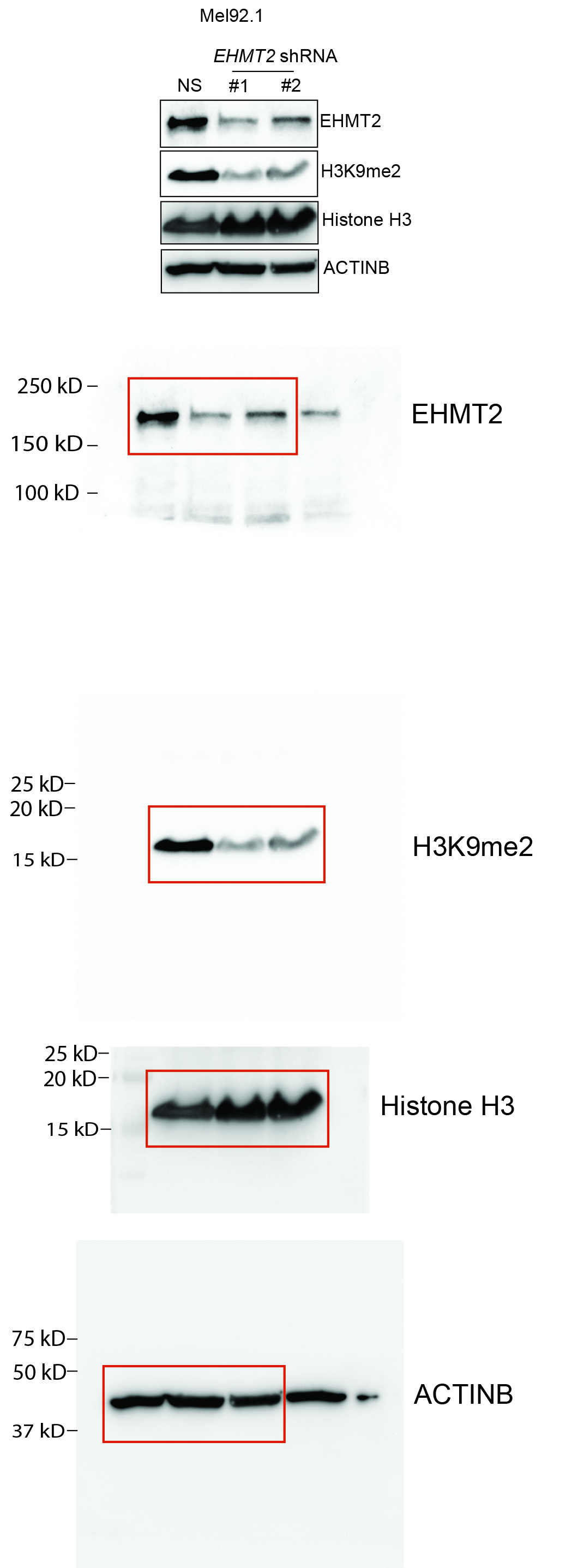

Supplement: Supplementary file 6 — Source data Fig. 1 [file 44321_2025_357_MOESM6_ESM.zip › Figure 1/1D/FIgure 1D-Mel92.1 Western blots.tif]

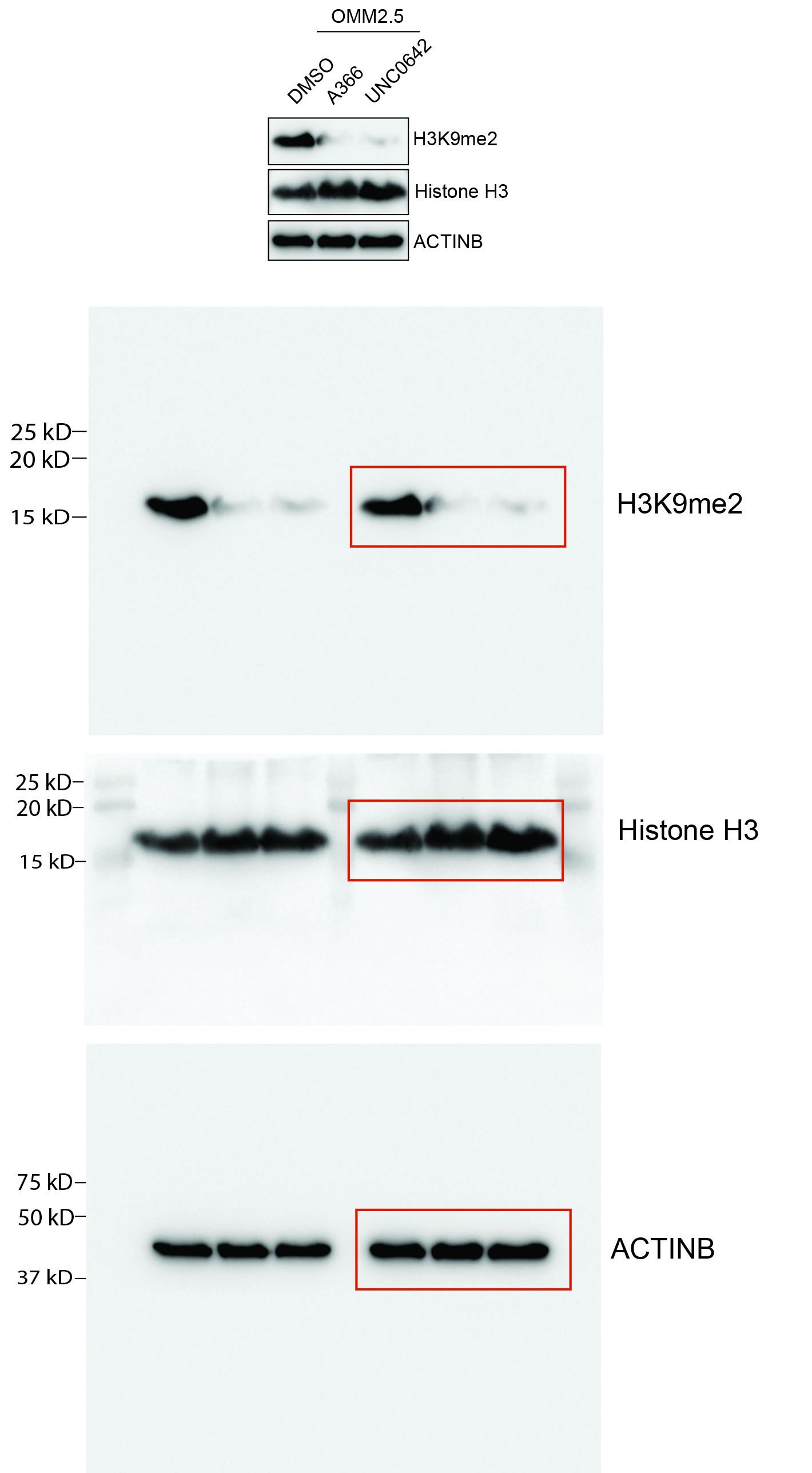

Supplement: Supplementary file 6 — Source data Fig. 1 [file 44321_2025_357_MOESM6_ESM.zip › Figure 1/1B/OMM2.5/FIgure 1B-OMM2.5 Western blots.tif]

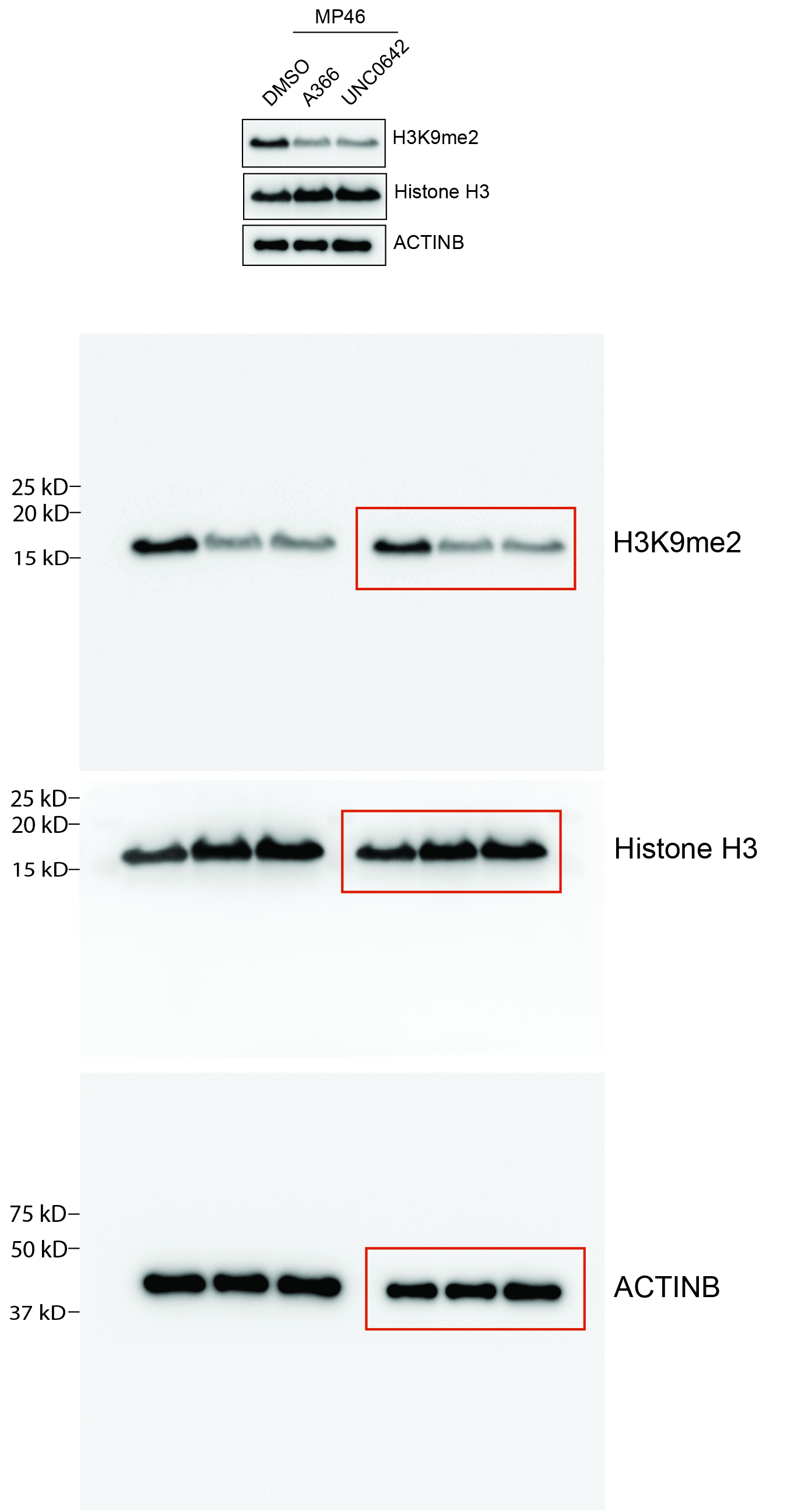

Supplement: Supplementary file 6 — Source data Fig. 1 [file 44321_2025_357_MOESM6_ESM.zip › Figure 1/1B/MP46/FIgure 1B-MP46 Western blots.tif]

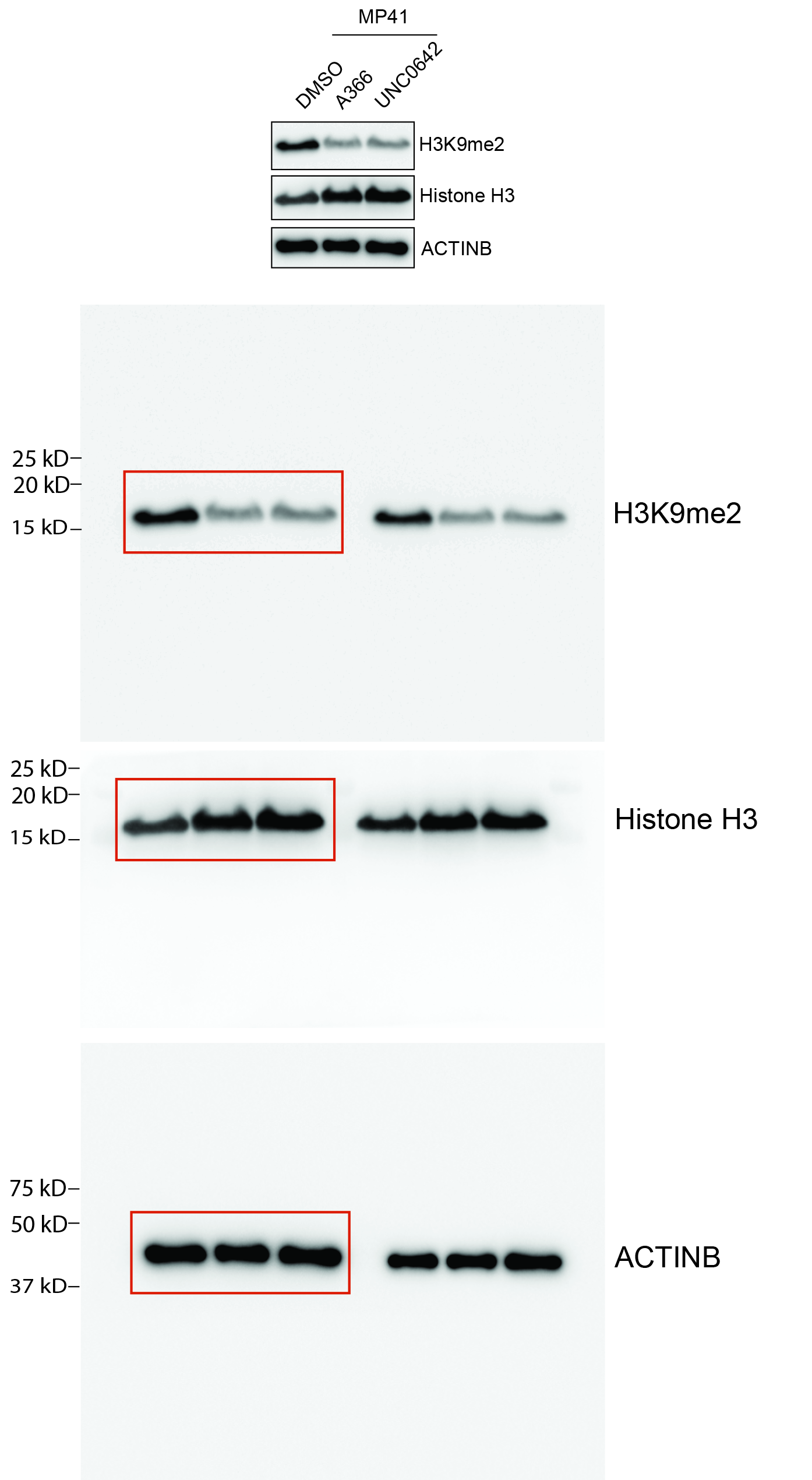

Supplement: Supplementary file 6 — Source data Fig. 1 [file 44321_2025_357_MOESM6_ESM.zip › Figure 1/1B/MP41/FIgure 1B-MP41 Western blots.tif]

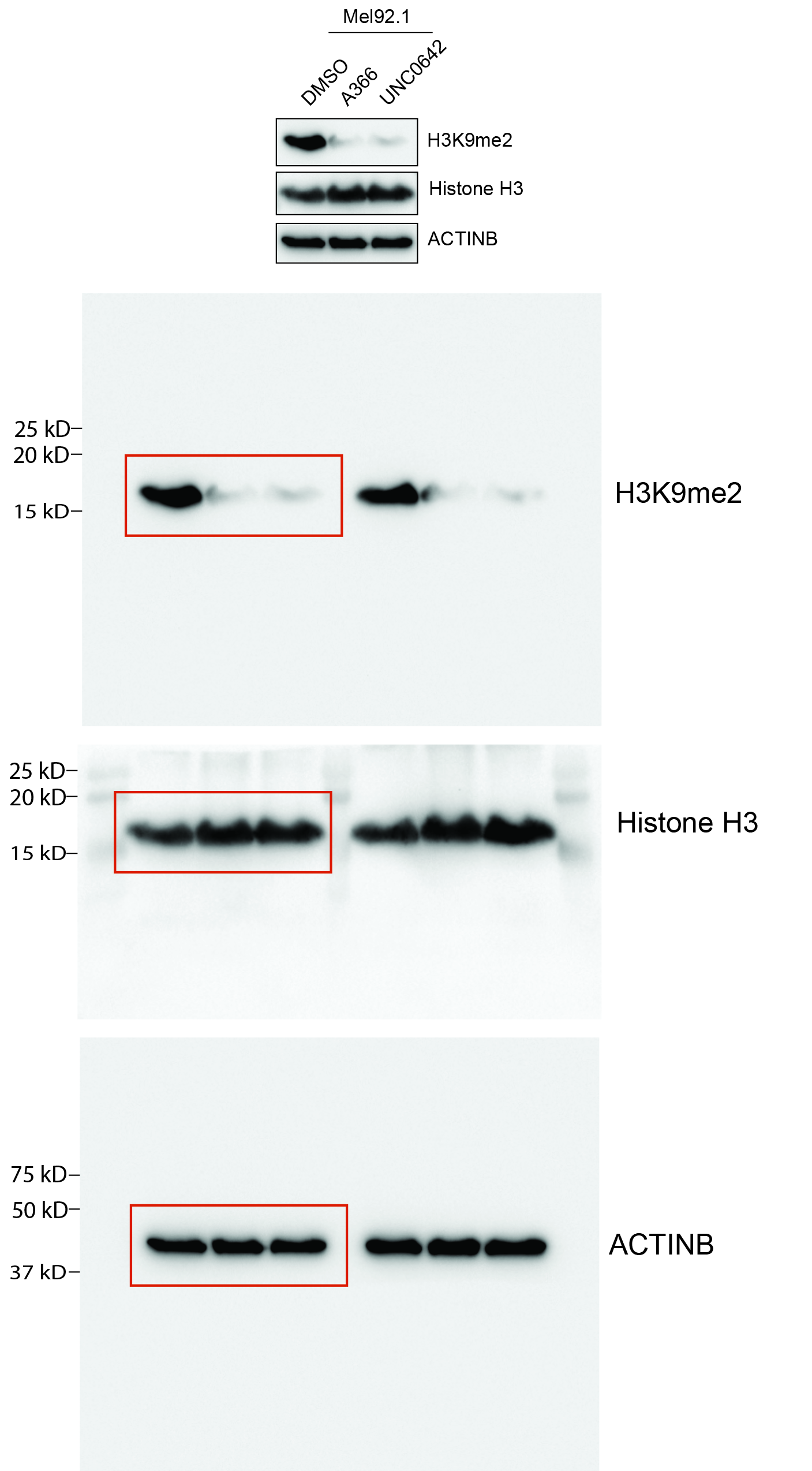

Supplement: Supplementary file 6 — Source data Fig. 1 [file 44321_2025_357_MOESM6_ESM.zip › Figure 1/1B/Mel92.1/FIgure 1B-Mel92.1 Western blots.tif]

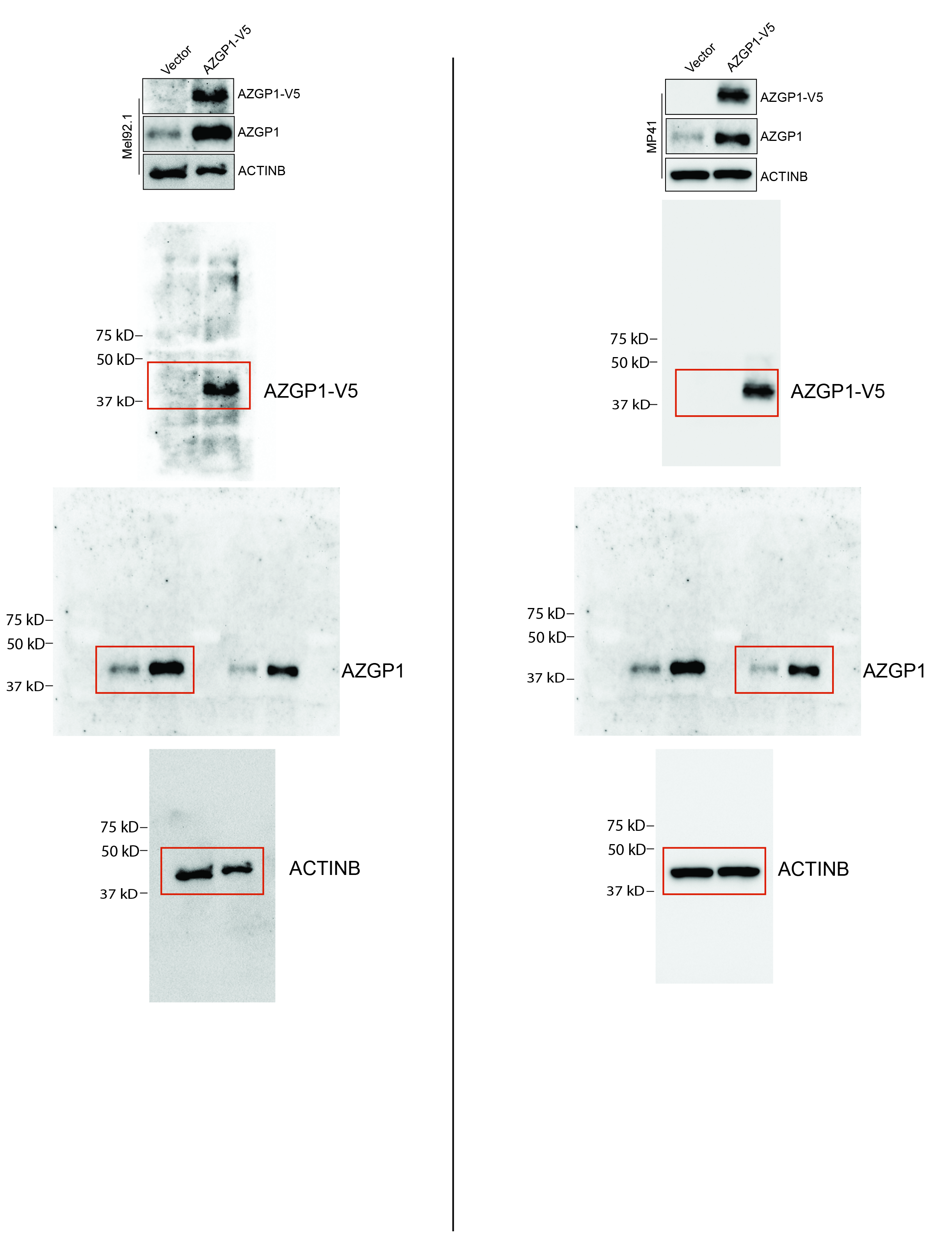

Supplement: Supplementary file 7 — Source data Fig. 2 [file 44321_2025_357_MOESM7_ESM.zip › Figure 2/2I/FIgure 2I-Mel92.1 and MP41 Western blots.tif]

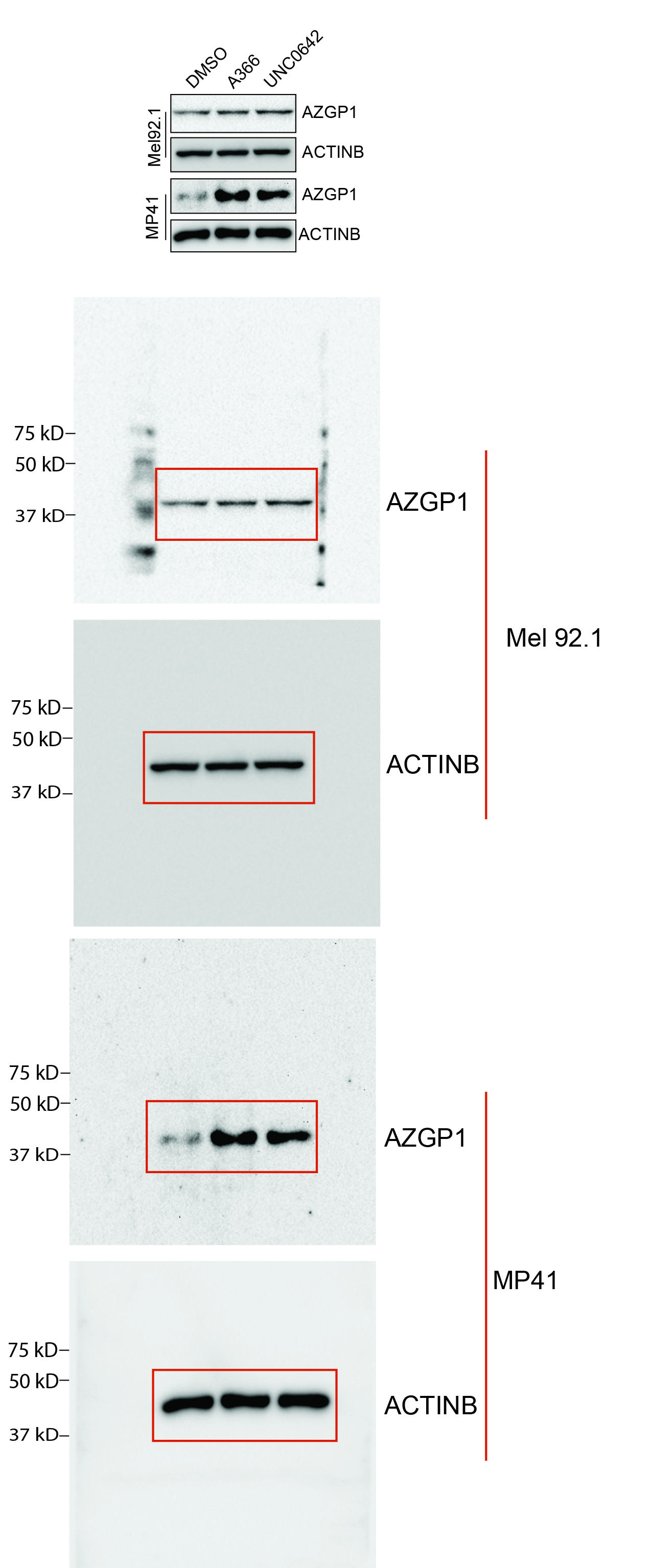

Supplement: Supplementary file 7 — Source data Fig. 2 [file 44321_2025_357_MOESM7_ESM.zip › Figure 2/2E/FIgure 2E-Mel92.1 and MP41 Western blots.tif]

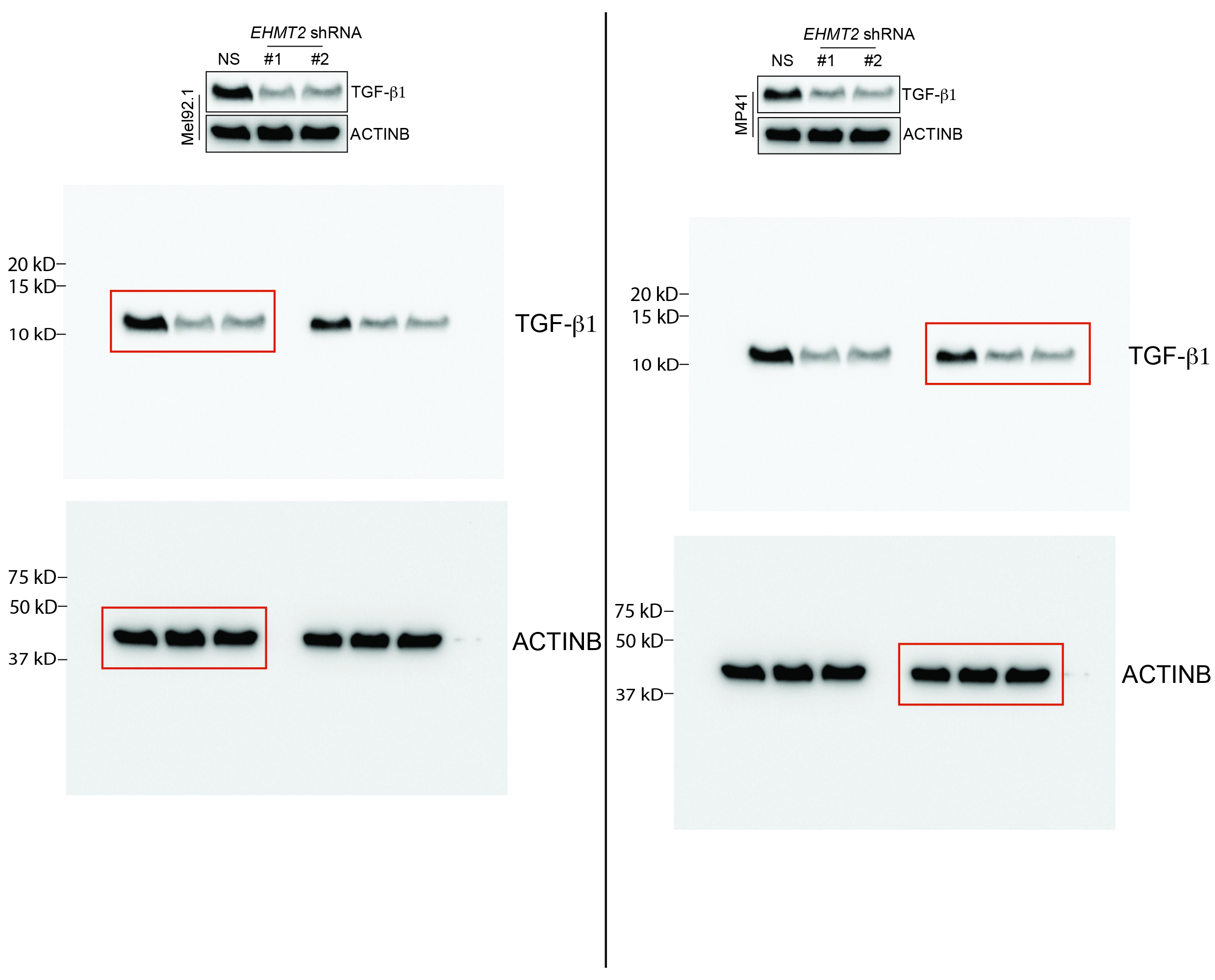

Supplement: Supplementary file 8 — Source data Fig. 3 [file 44321_2025_357_MOESM8_ESM.zip › Figure 3/3B/Figure 3B-Mel92.1 and MP41 Western blots.tif]

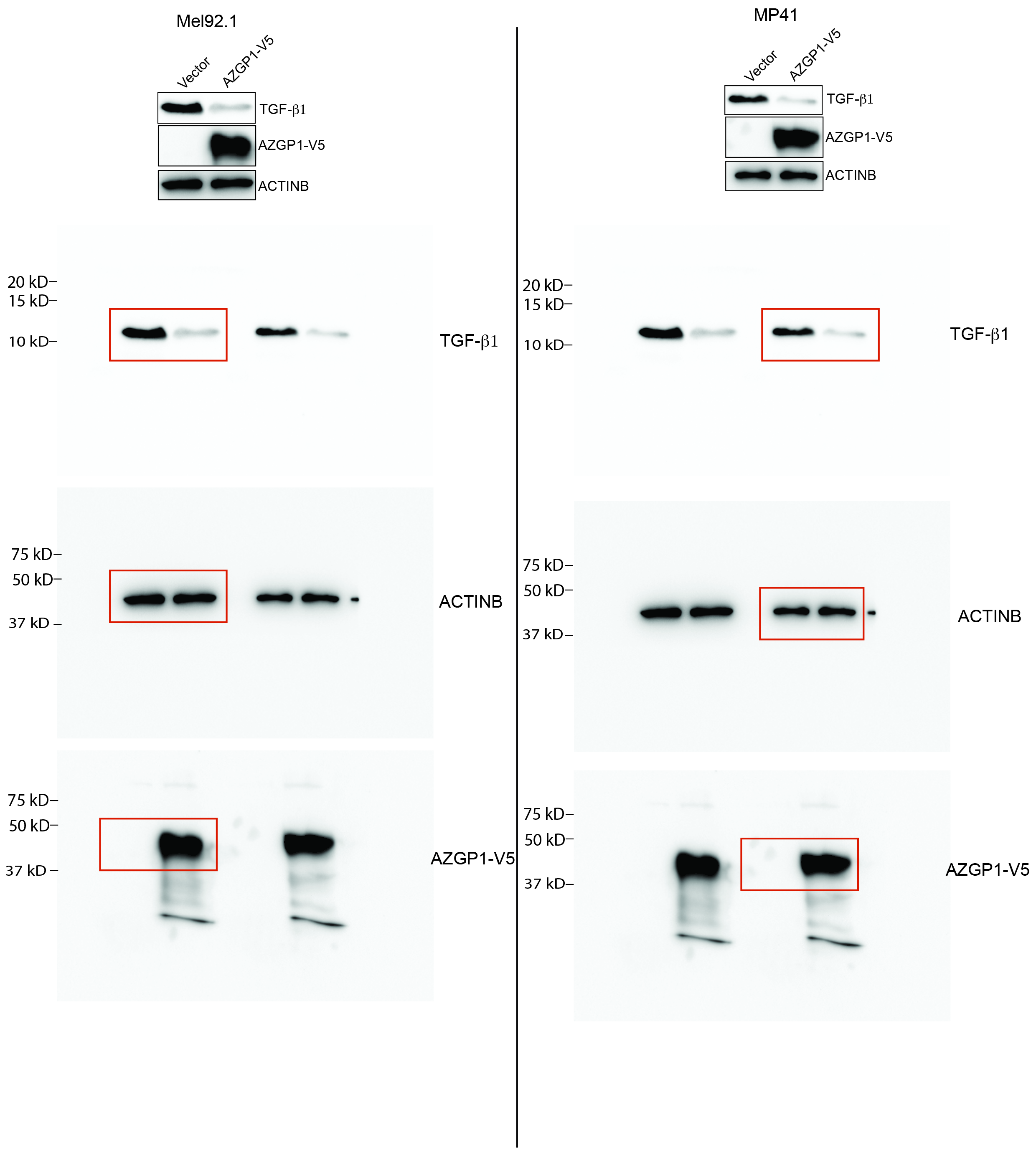

Supplement: Supplementary file 8 — Source data Fig. 3 [file 44321_2025_357_MOESM8_ESM.zip › Figure 3/3C/Figure 3C-Mel92.1 and MP41 Western blots.tif]

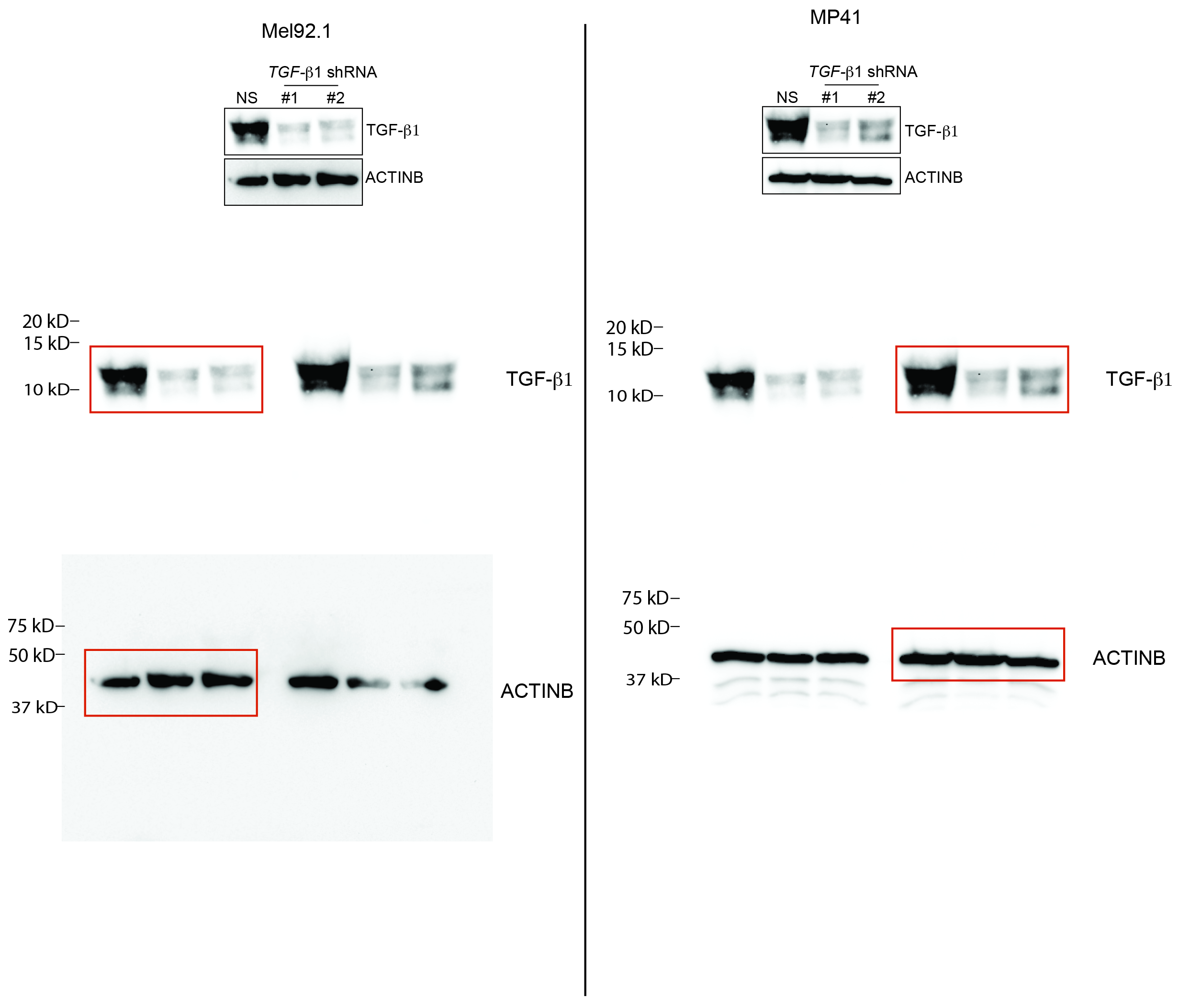

Supplement: Supplementary file 8 — Source data Fig. 3 [file 44321_2025_357_MOESM8_ESM.zip › Figure 3/3D/Figure 3D-Mel92.1 and MP41 Western blots.tif]

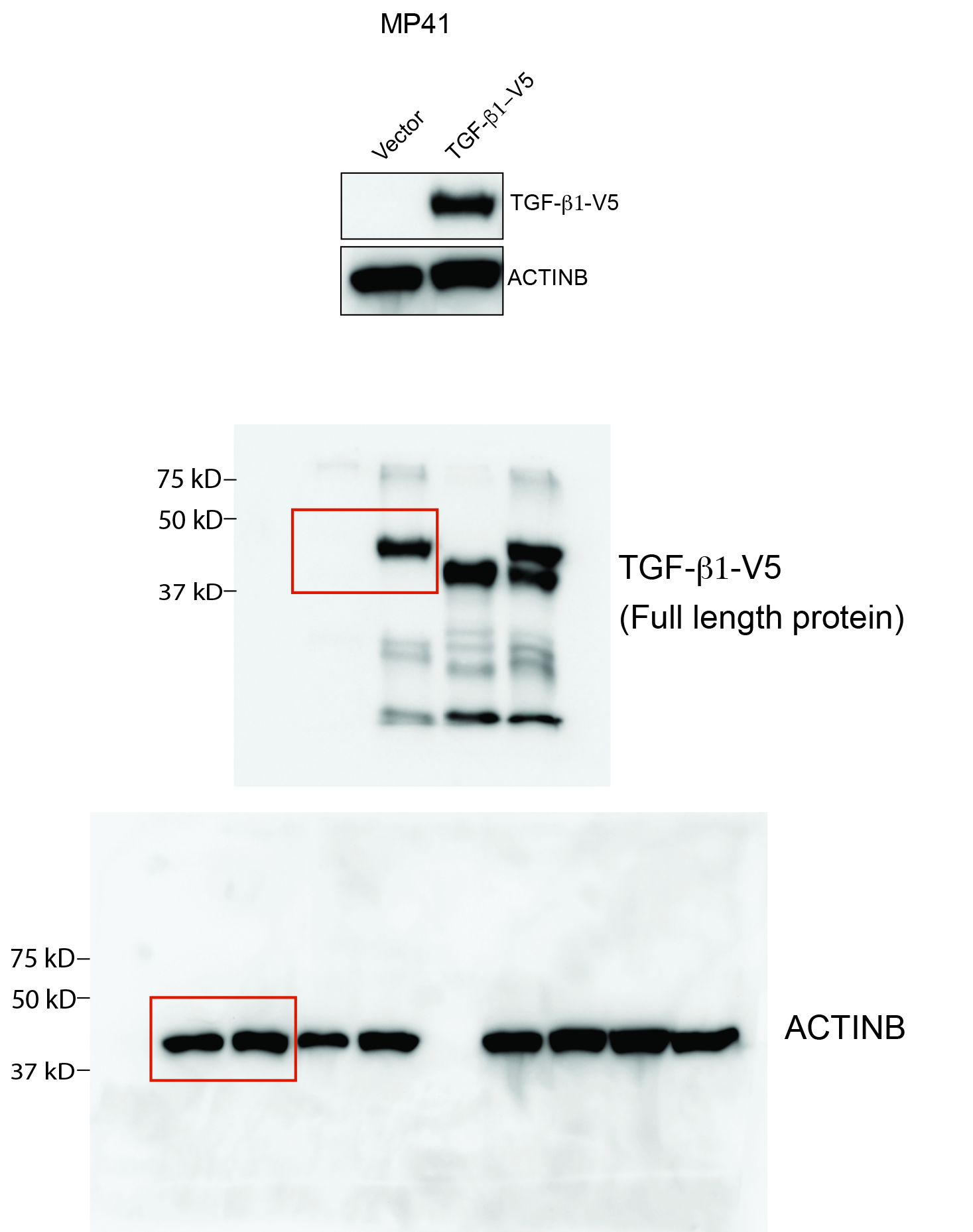

Supplement: Supplementary file 8 — Source data Fig. 3 [file 44321_2025_357_MOESM8_ESM.zip › Figure 3/3H/Figure 3H-MP41 Western blots.tif]

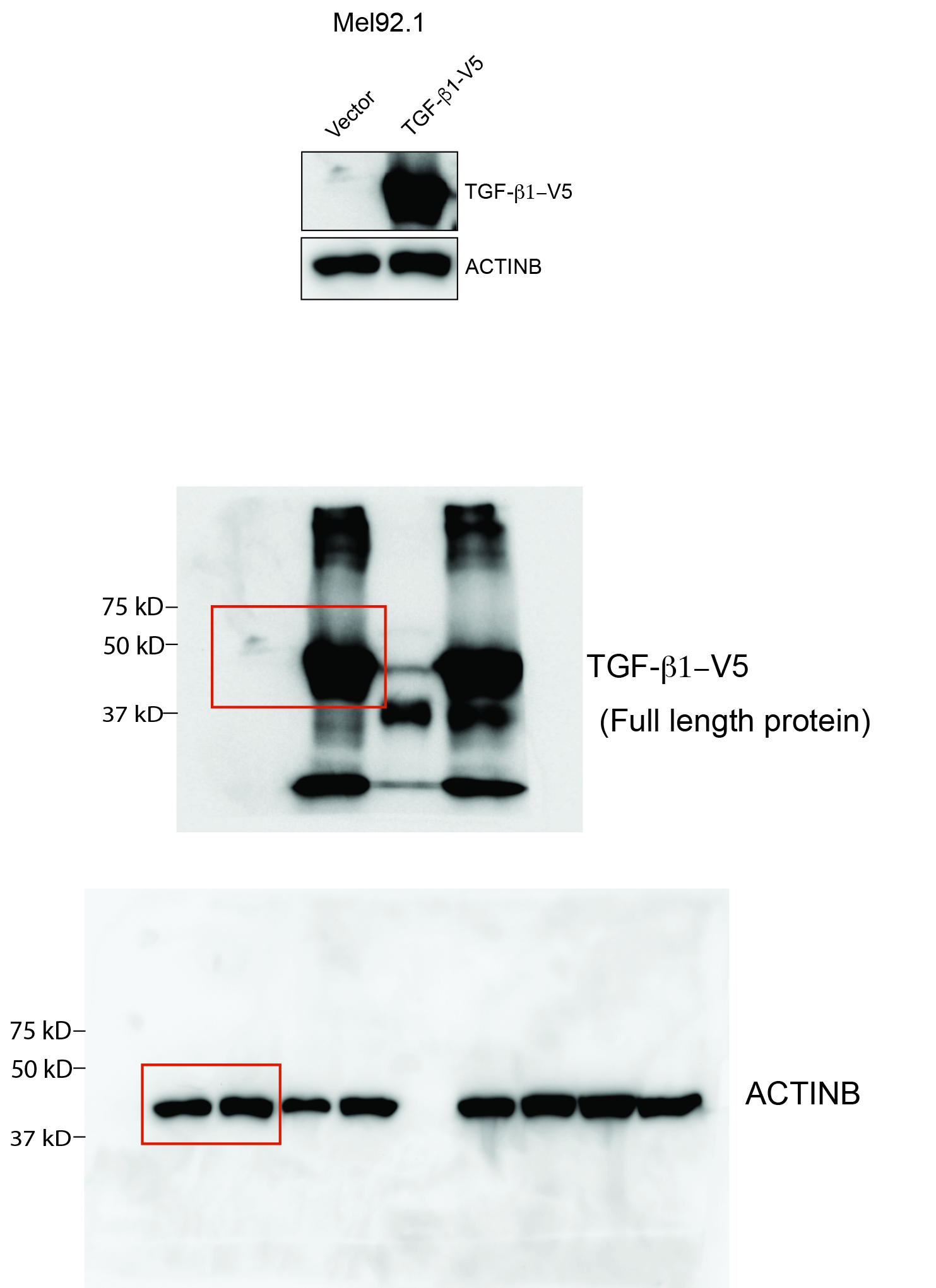

Supplement: Supplementary file 8 — Source data Fig. 3 [file 44321_2025_357_MOESM8_ESM.zip › Figure 3/3F/Figure 3F-Mel92.1 Western blot.tif]

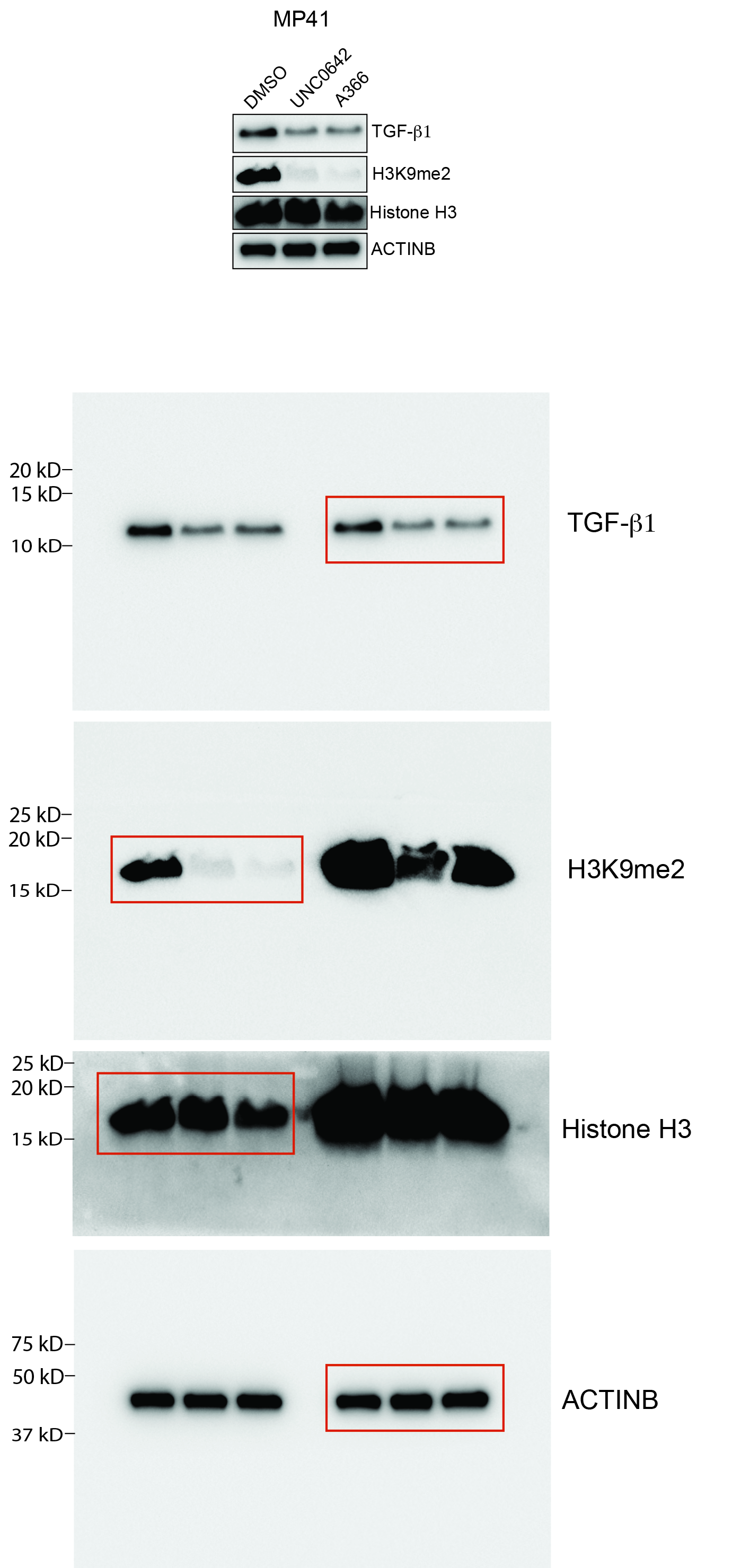

Supplement: Supplementary file 8 — Source data Fig. 3 [file 44321_2025_357_MOESM8_ESM.zip › Figure 3/3A/MP41/Figure 3A-MP41 Western blots.tif]

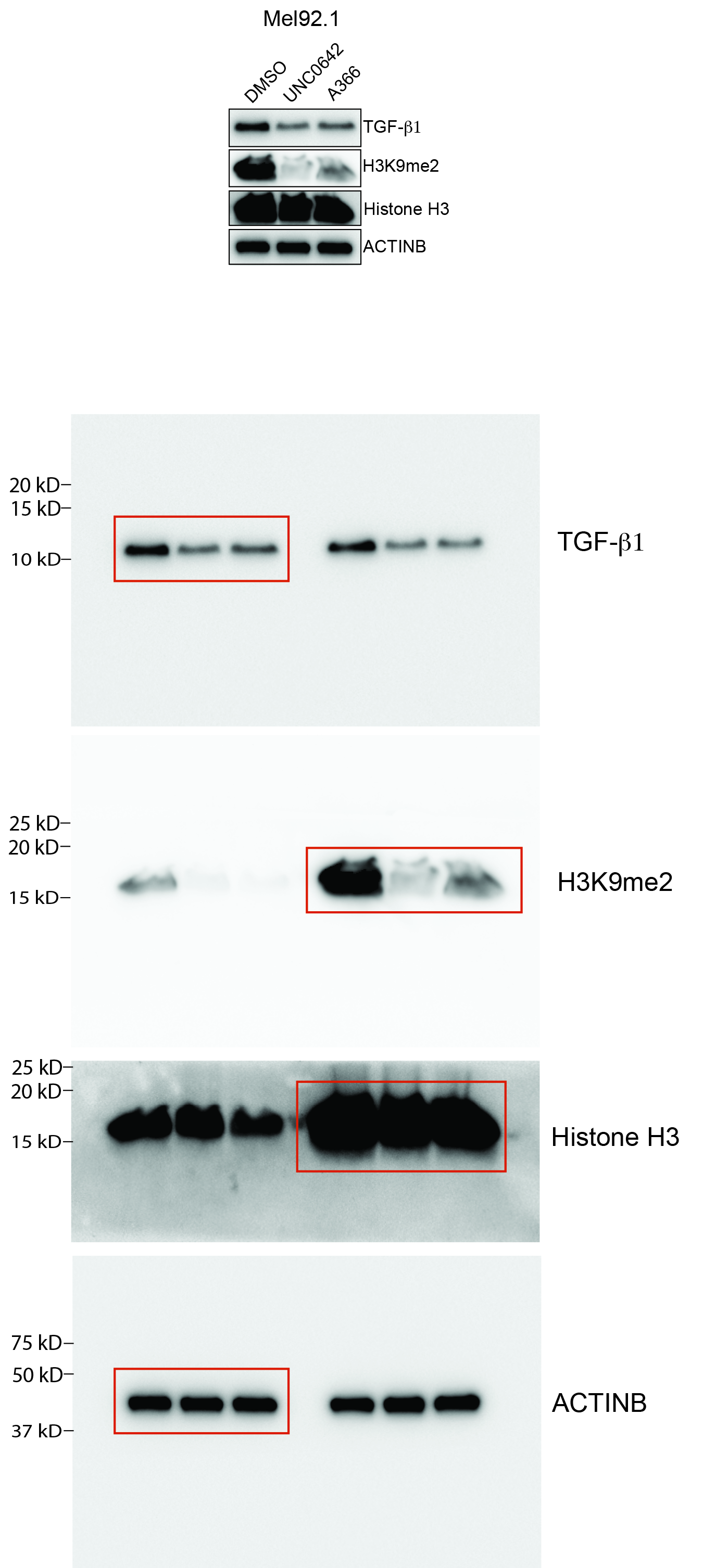

Supplement: Supplementary file 8 — Source data Fig. 3 [file 44321_2025_357_MOESM8_ESM.zip › Figure 3/3A/Mel92.1/Figure 3A-Mel92.1 Western blots.tif]

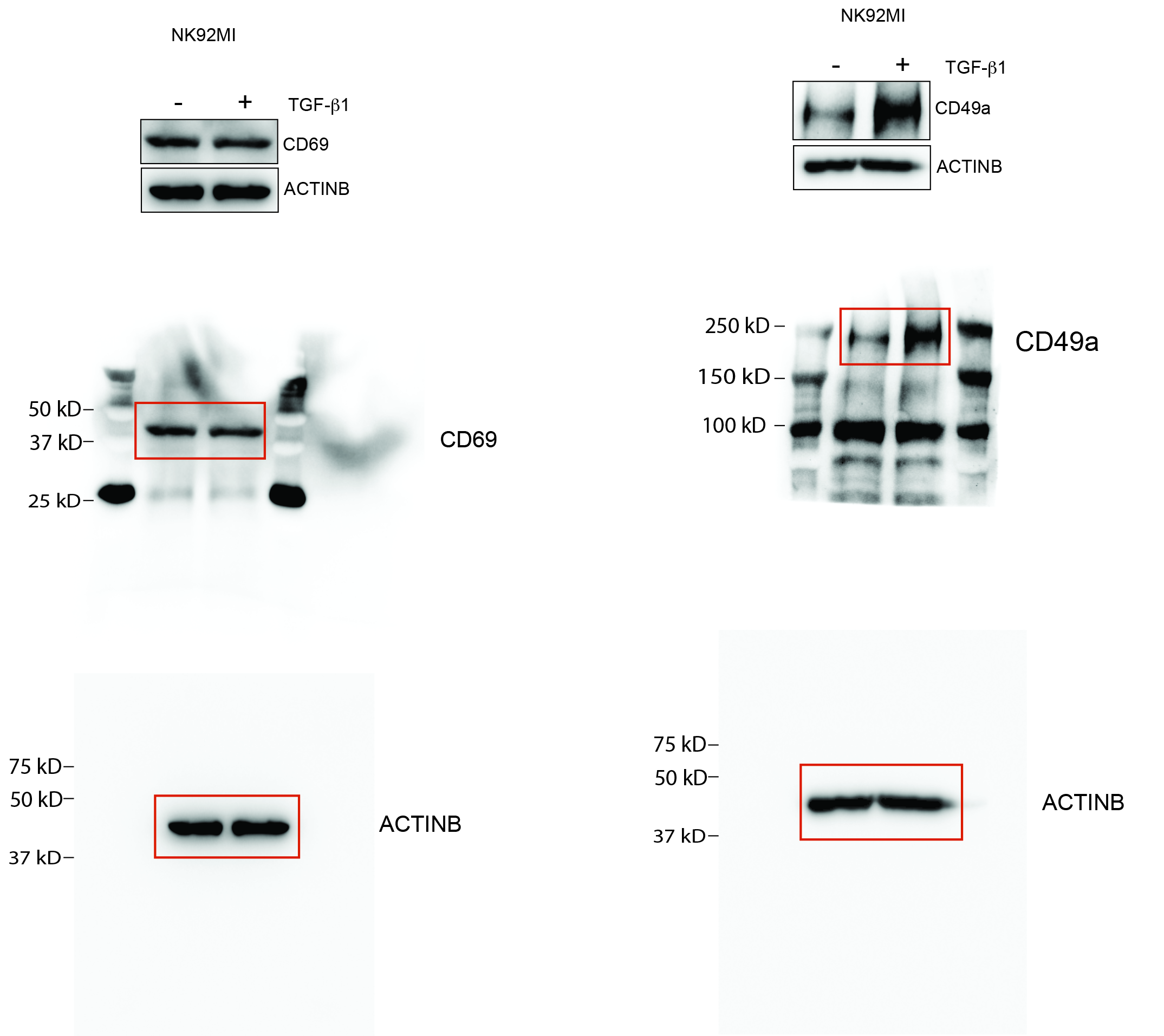

Supplement: Supplementary file 9 — Source data Fig. 4 [file 44321_2025_357_MOESM9_ESM.zip › Figure 4/4K/Figure 4K CD69 and CD49a panel Western blots.tif]

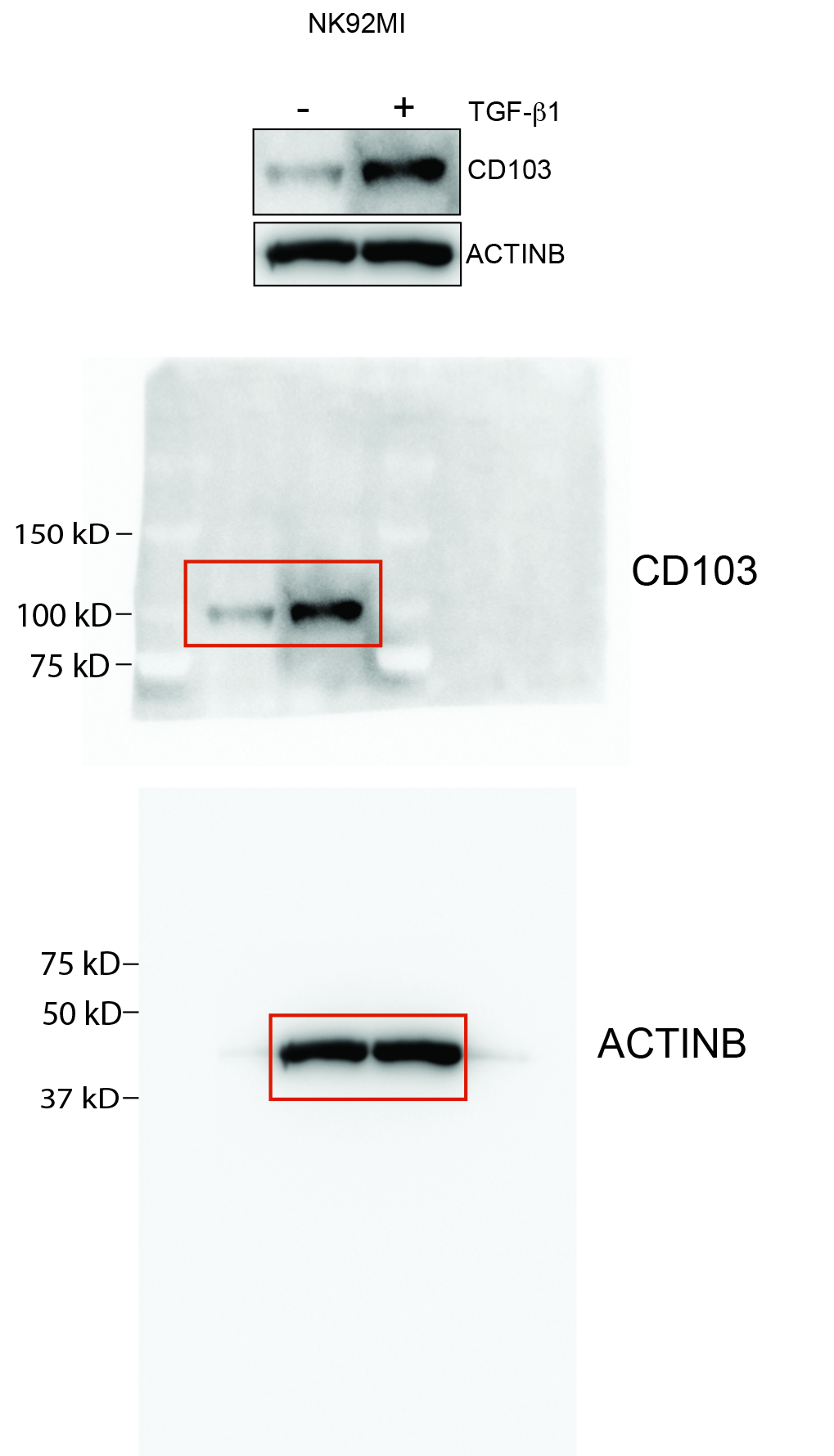

Supplement: Supplementary file 9 — Source data Fig. 4 [file 44321_2025_357_MOESM9_ESM.zip › Figure 4/4K/Figure 4K CD103 panel Western blots.tif]

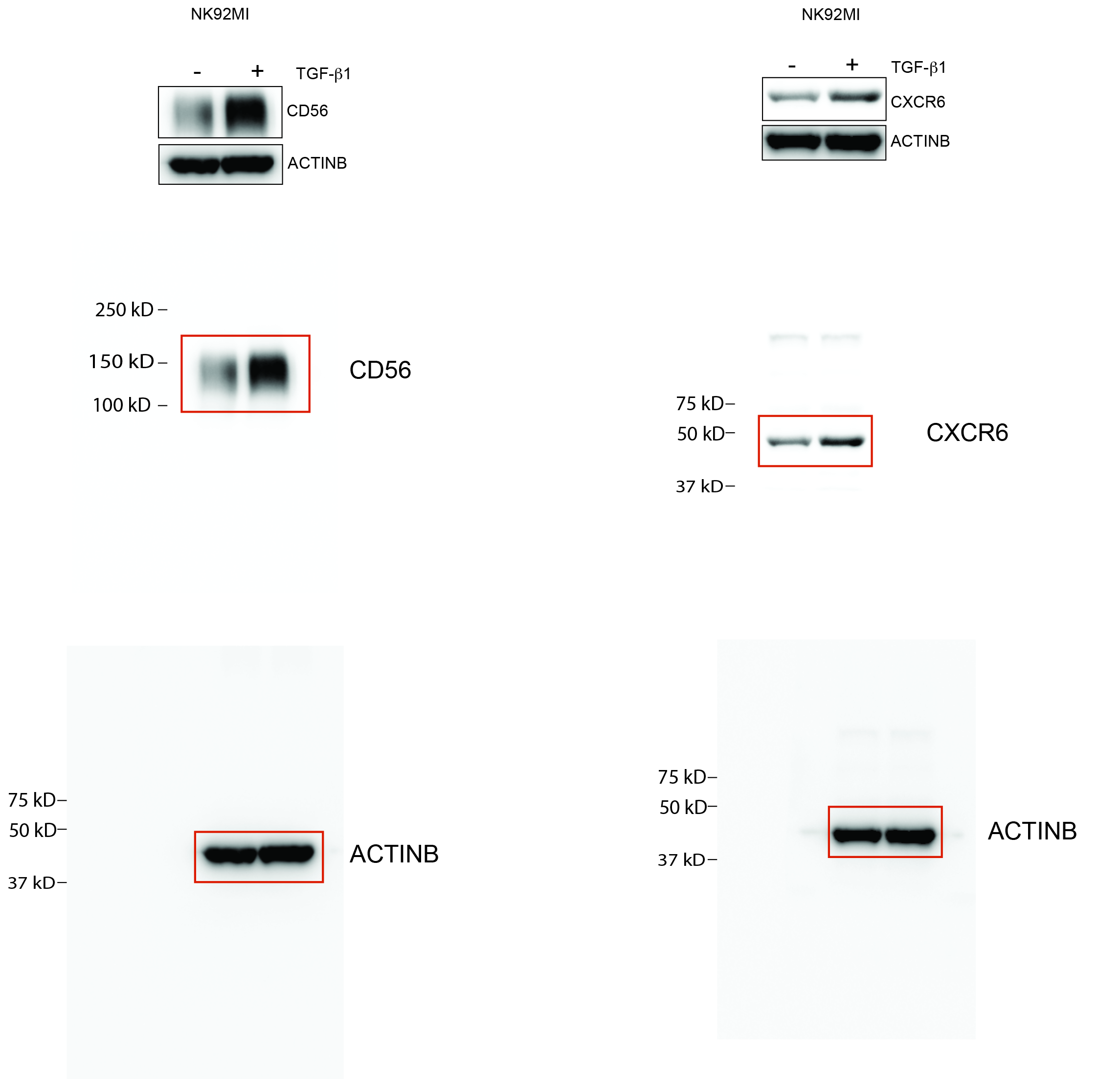

Supplement: Supplementary file 9 — Source data Fig. 4 [file 44321_2025_357_MOESM9_ESM.zip › Figure 4/4K/Figure 4K CD56 and CXCR6 panel Western blots.tif]

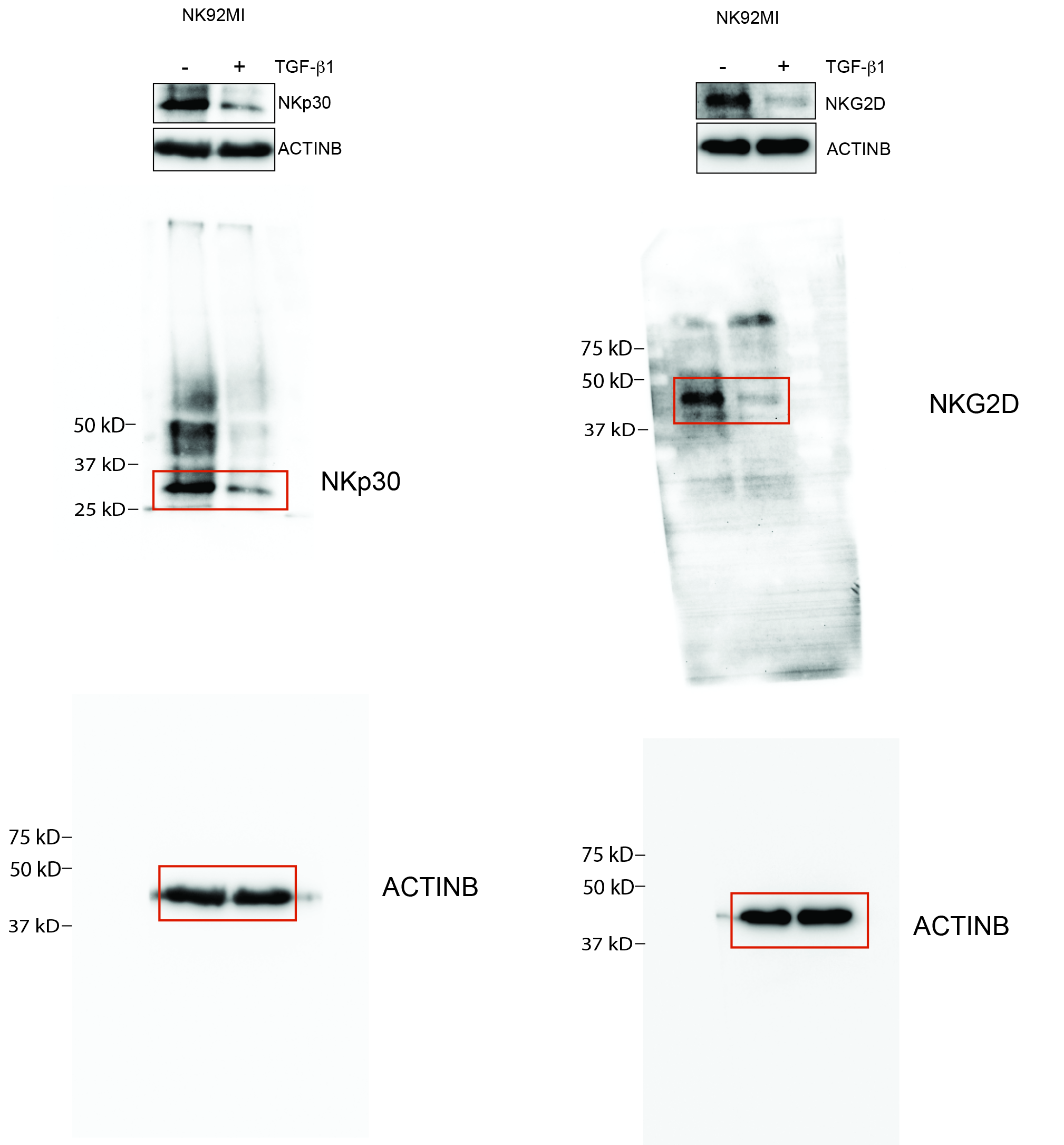

Supplement: Supplementary file 9 — Source data Fig. 4 [file 44321_2025_357_MOESM9_ESM.zip › Figure 4/4J/Figure 4J Western blots.tif]

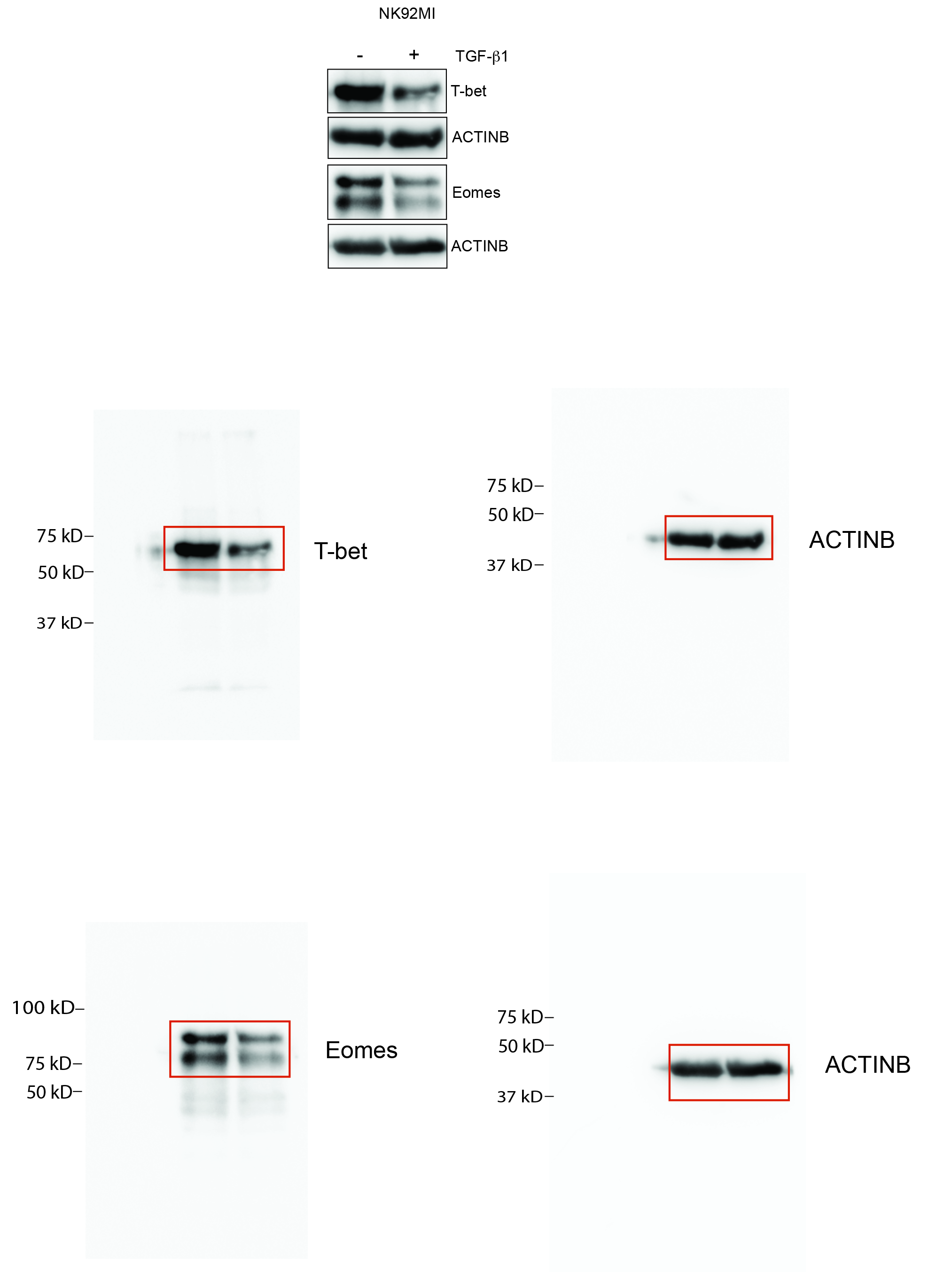

Supplement: Supplementary file 9 — Source data Fig. 4 [file 44321_2025_357_MOESM9_ESM.zip › Figure 4/4I/Figure 4I Western blots.tif]

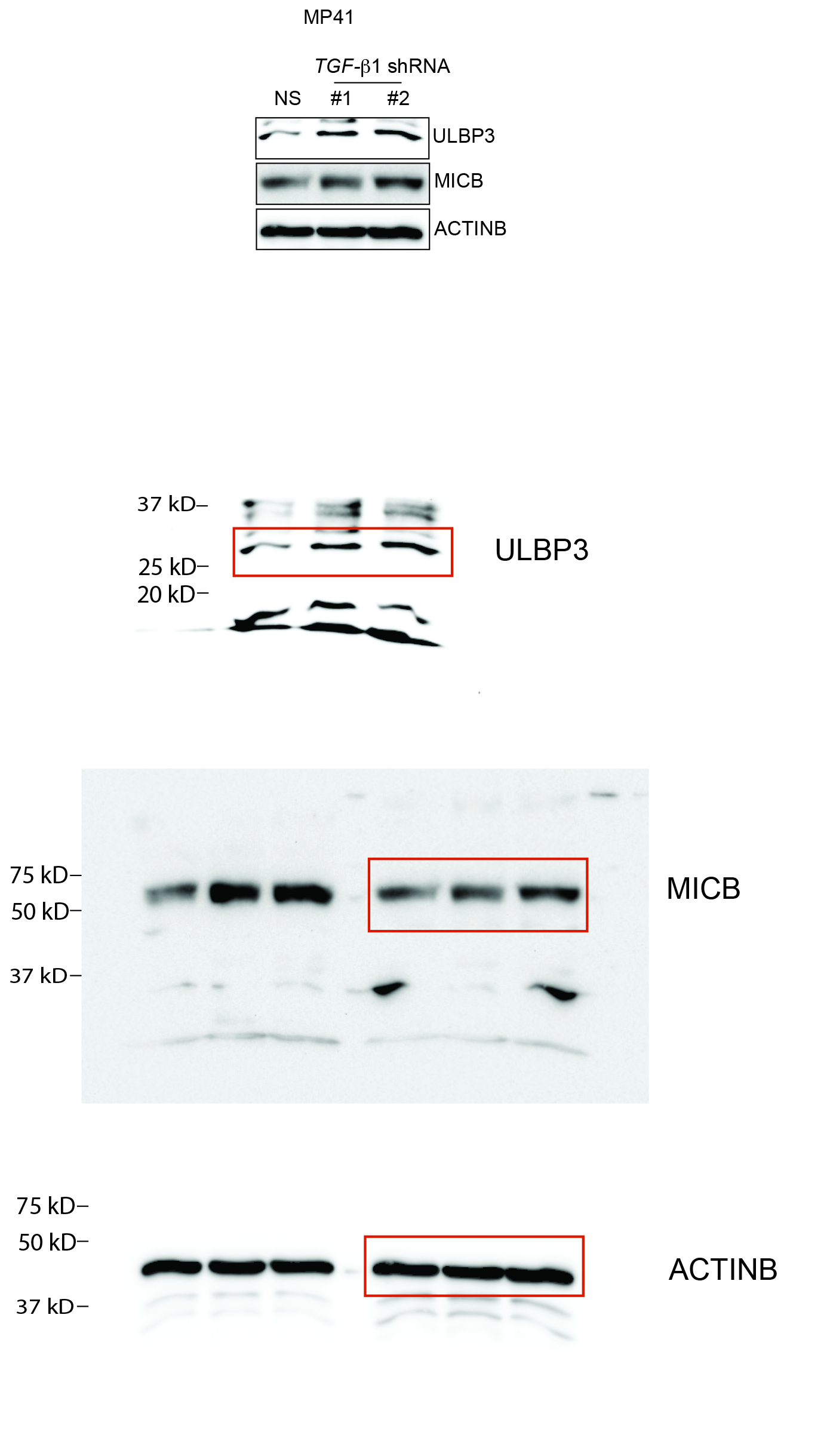

Supplement: Supplementary file 9 — Source data Fig. 4 [file 44321_2025_357_MOESM9_ESM.zip › Figure 4/4B/MP41/Figure 4B-MP41 Western blots.tif]

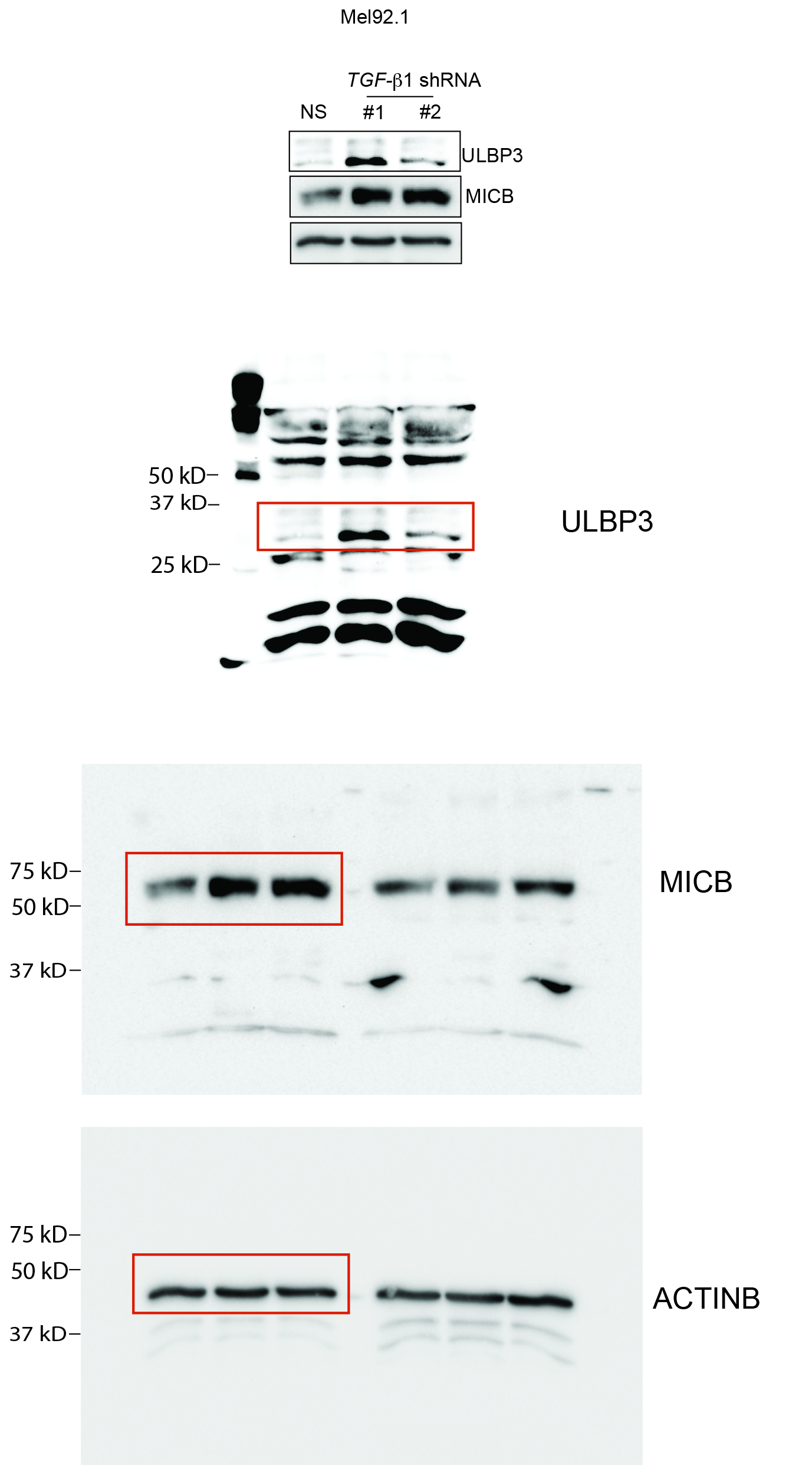

Supplement: Supplementary file 9 — Source data Fig. 4 [file 44321_2025_357_MOESM9_ESM.zip › Figure 4/4B/Mel92.1/Figure 4B-Mel92.1 Western blots.tif]

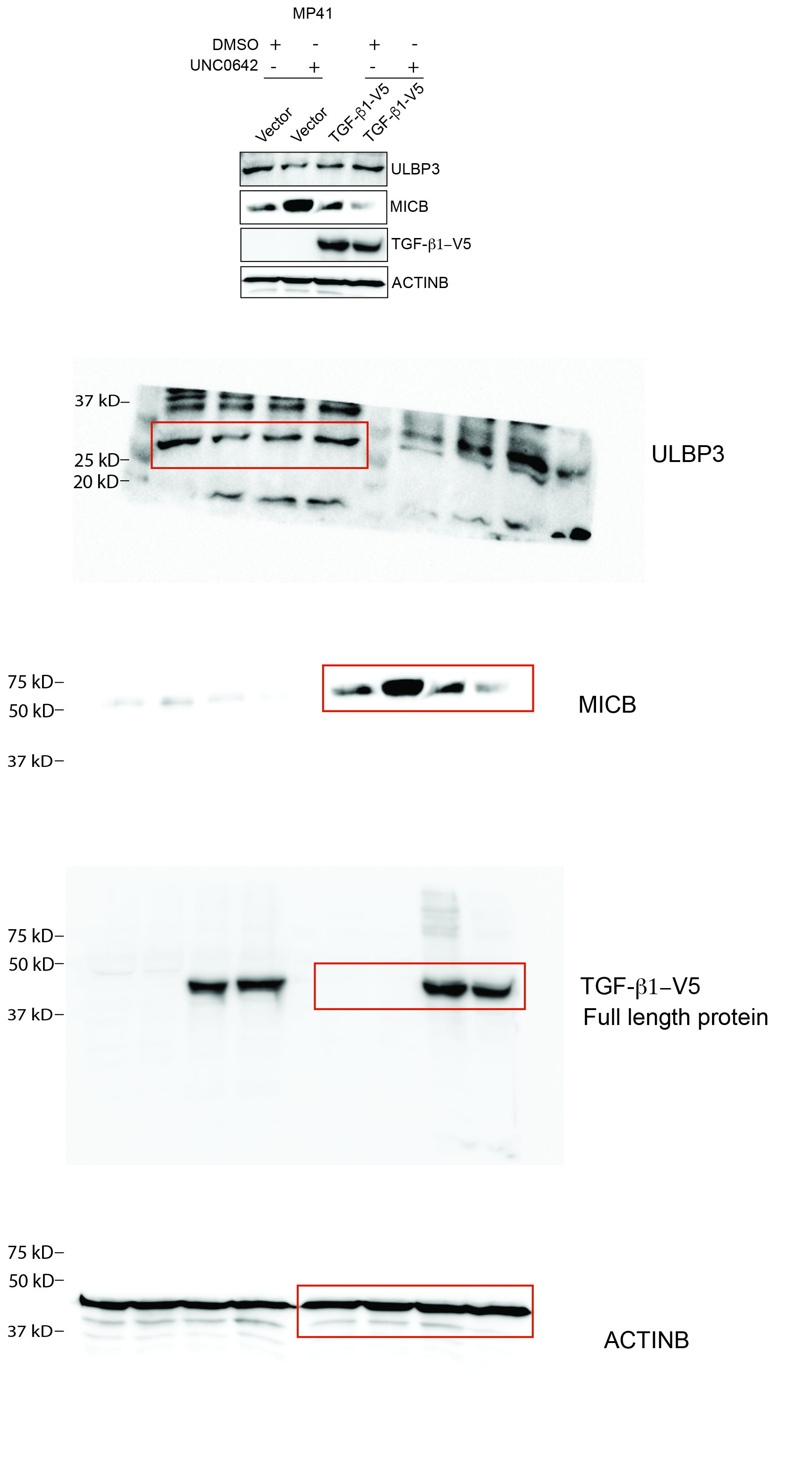

Supplement: Supplementary file 9 — Source data Fig. 4 [file 44321_2025_357_MOESM9_ESM.zip › Figure 4/4C/MP41/Figure 4C-MP41 Western blots.tif]

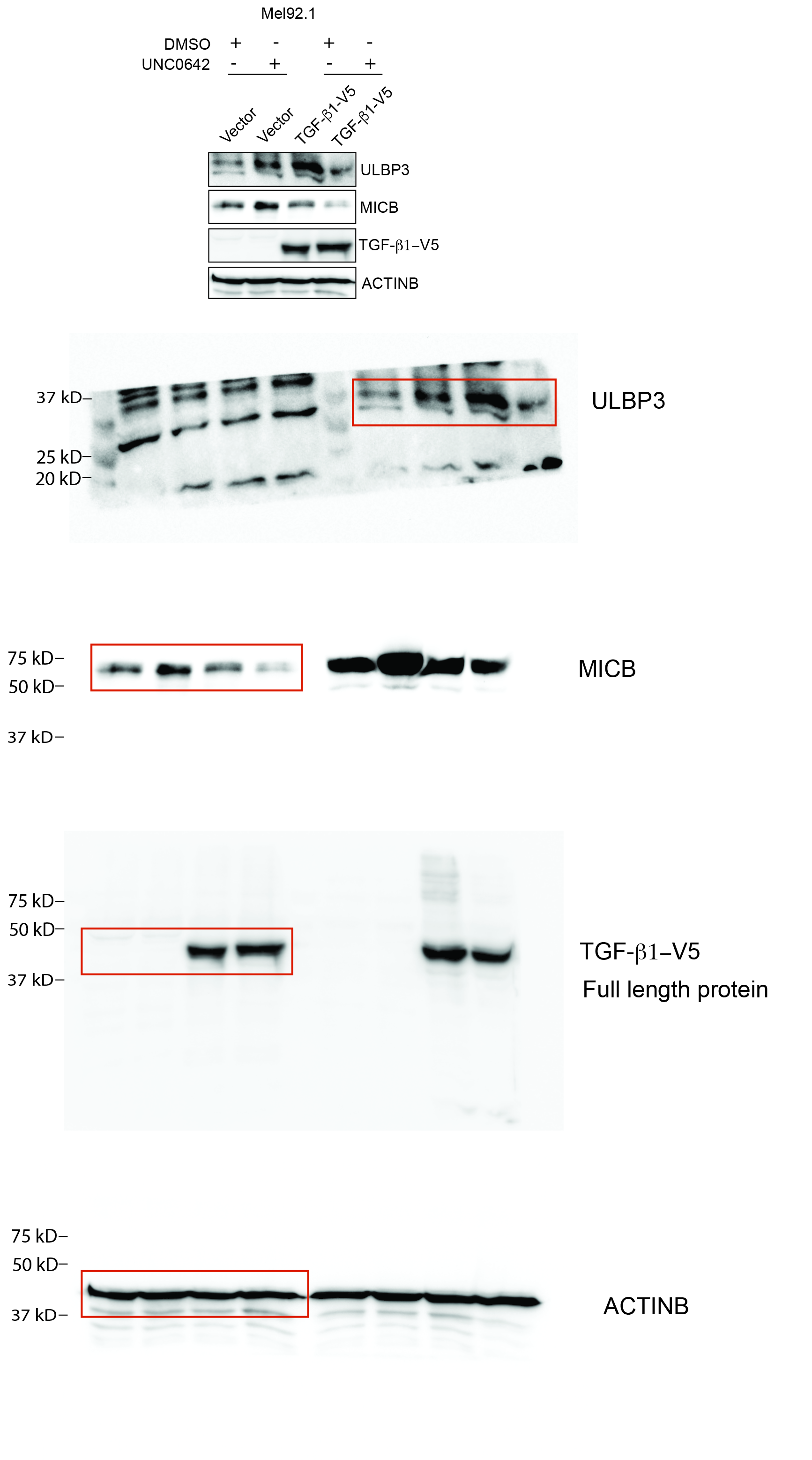

Supplement: Supplementary file 9 — Source data Fig. 4 [file 44321_2025_357_MOESM9_ESM.zip › Figure 4/4C/Mel92.1/Figure 4C-Mel92.1 Western blots.tif]

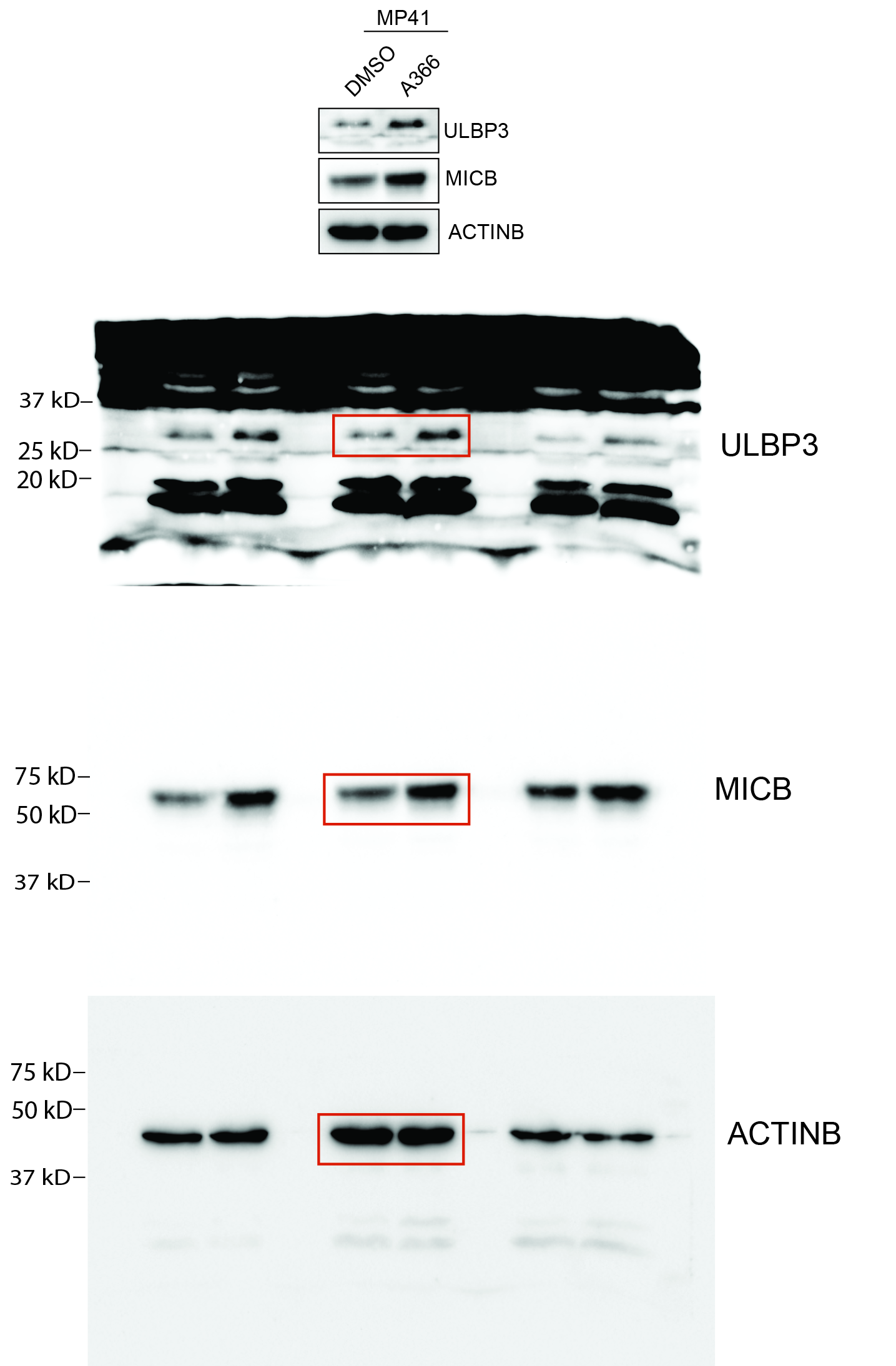

Supplement: Supplementary file 9 — Source data Fig. 4 [file 44321_2025_357_MOESM9_ESM.zip › Figure 4/4A/MP41 A366 treatment/Figure 4A-MP41 A366 treatment Western blots.tif]

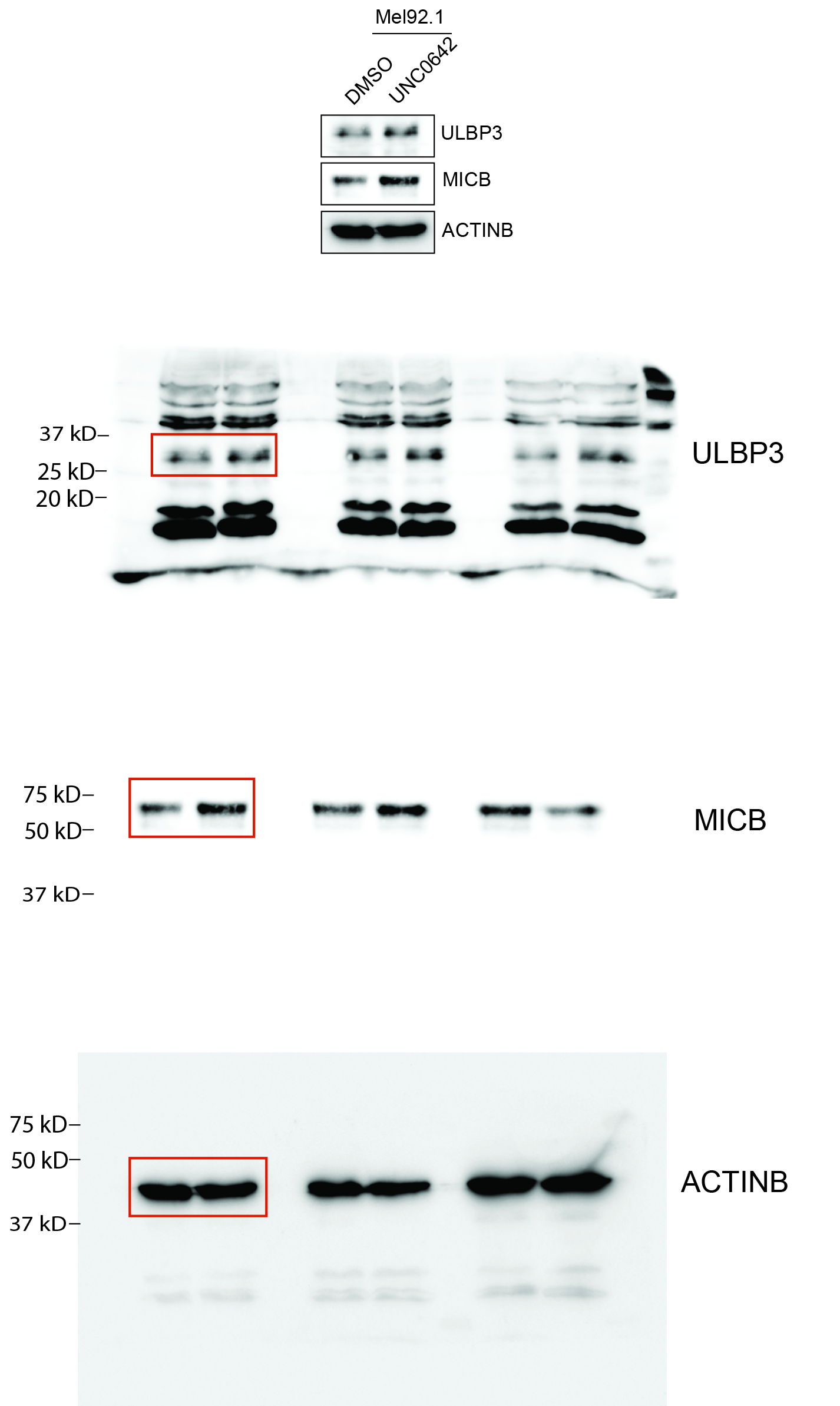

Supplement: Supplementary file 9 — Source data Fig. 4 [file 44321_2025_357_MOESM9_ESM.zip › Figure 4/4A/Mel92.1 UNC0642 treatment/Figure 4A-Mel92.1 UNC0642 treatment Western blots.tif]

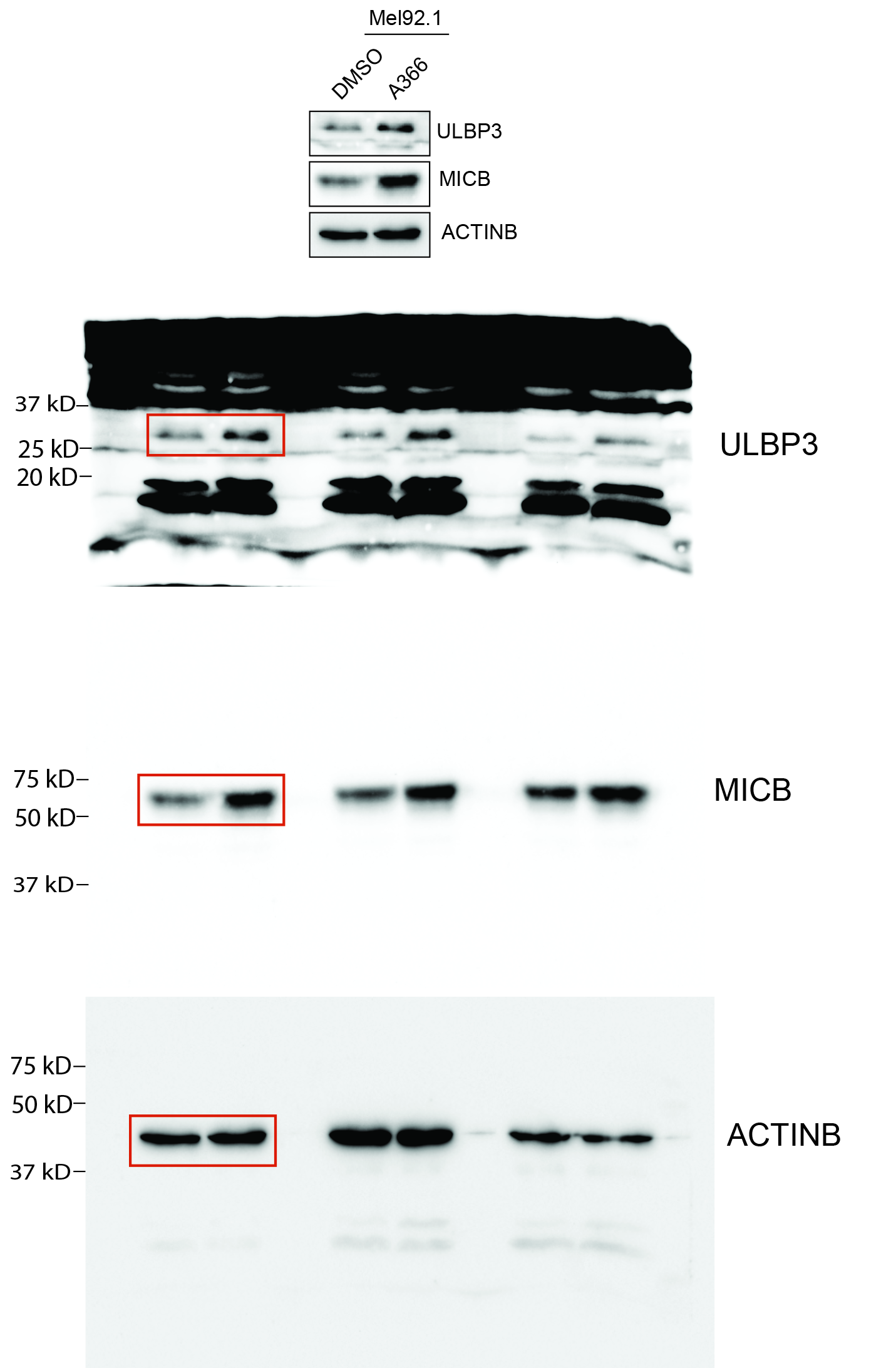

Supplement: Supplementary file 9 — Source data Fig. 4 [file 44321_2025_357_MOESM9_ESM.zip › Figure 4/4A/Mel92.1 A366 treatment/Figure 4A-Mel92.1 A366 treatment Western blots.tif]

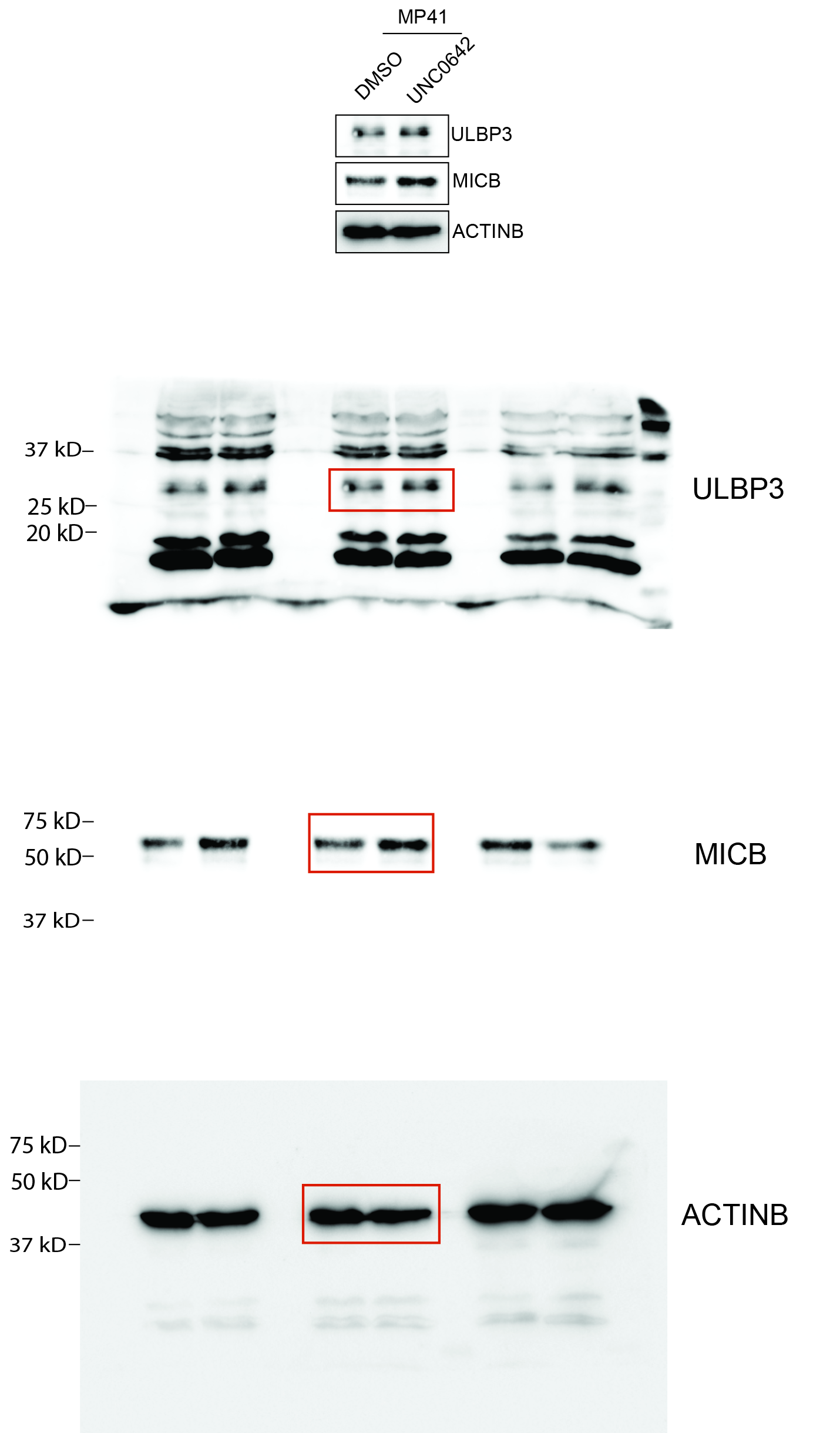

Supplement: Supplementary file 9 — Source data Fig. 4 [file 44321_2025_357_MOESM9_ESM.zip › Figure 4/4A/MP41 UNC0642 treatment /Figure 4A-MP41 UNC0642 treatment Western blots.tif]

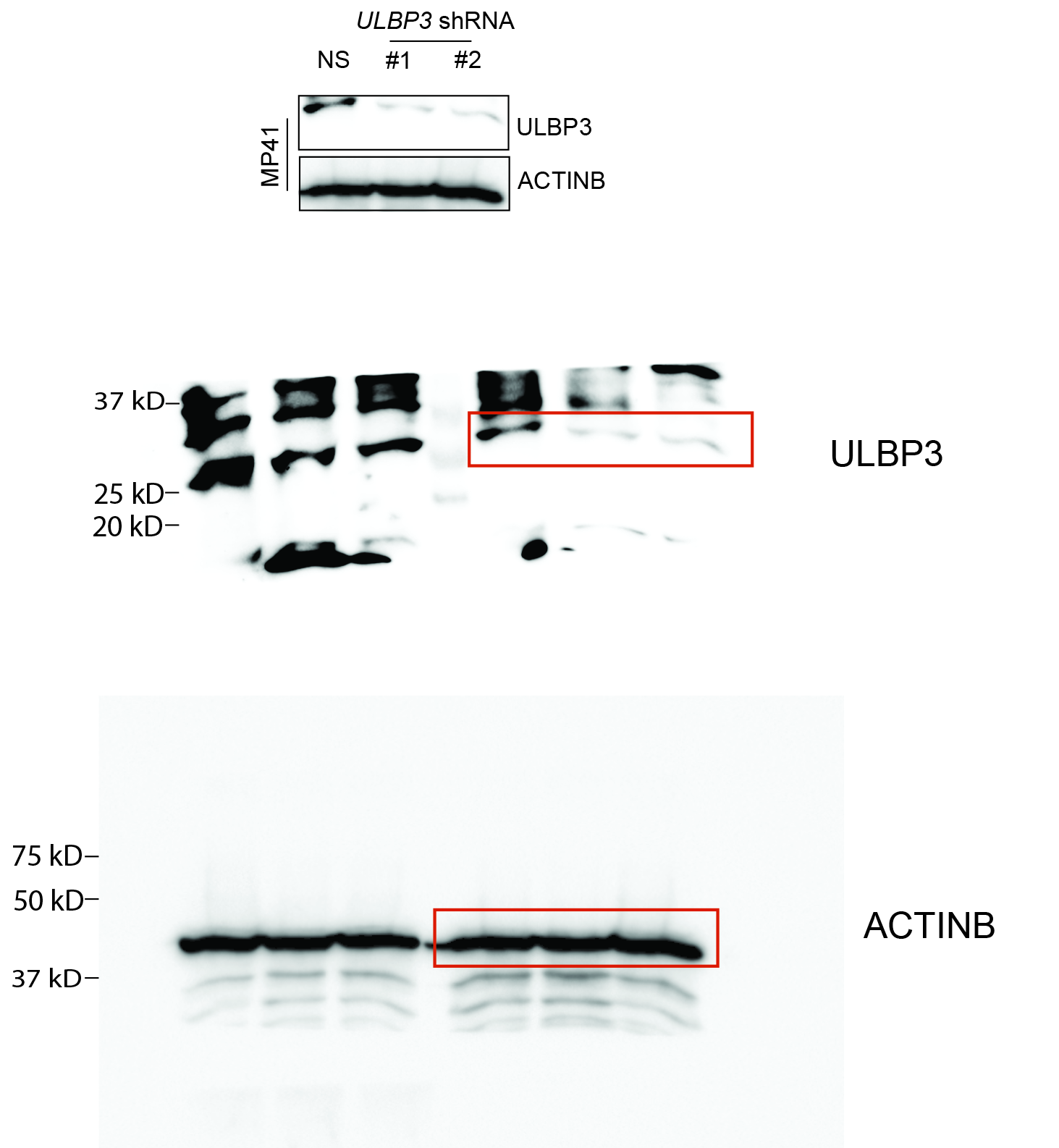

Supplement: Supplementary file 9 — Source data Fig. 4 [file 44321_2025_357_MOESM9_ESM.zip › Figure 4/4D/ULBP3 shRNA panel blots/MP41/Figure 4D-MP41 ULBP3 shRNA panel Western blots.tif]

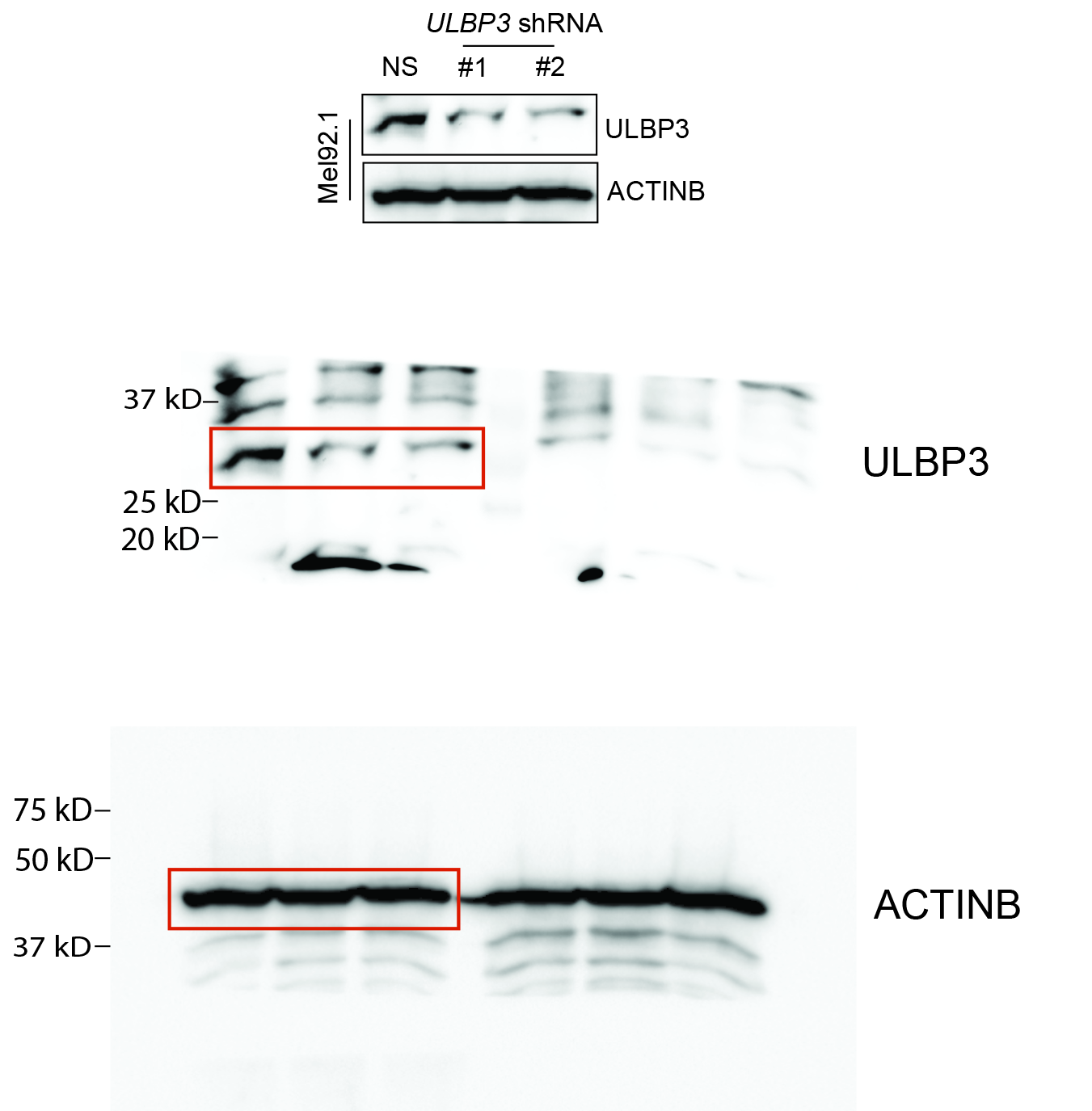

Supplement: Supplementary file 9 — Source data Fig. 4 [file 44321_2025_357_MOESM9_ESM.zip › Figure 4/4D/ULBP3 shRNA panel blots/Mel92.1/Figure 4D-Mel92.1 ULBP3 shRNA panel Western blots.tif]

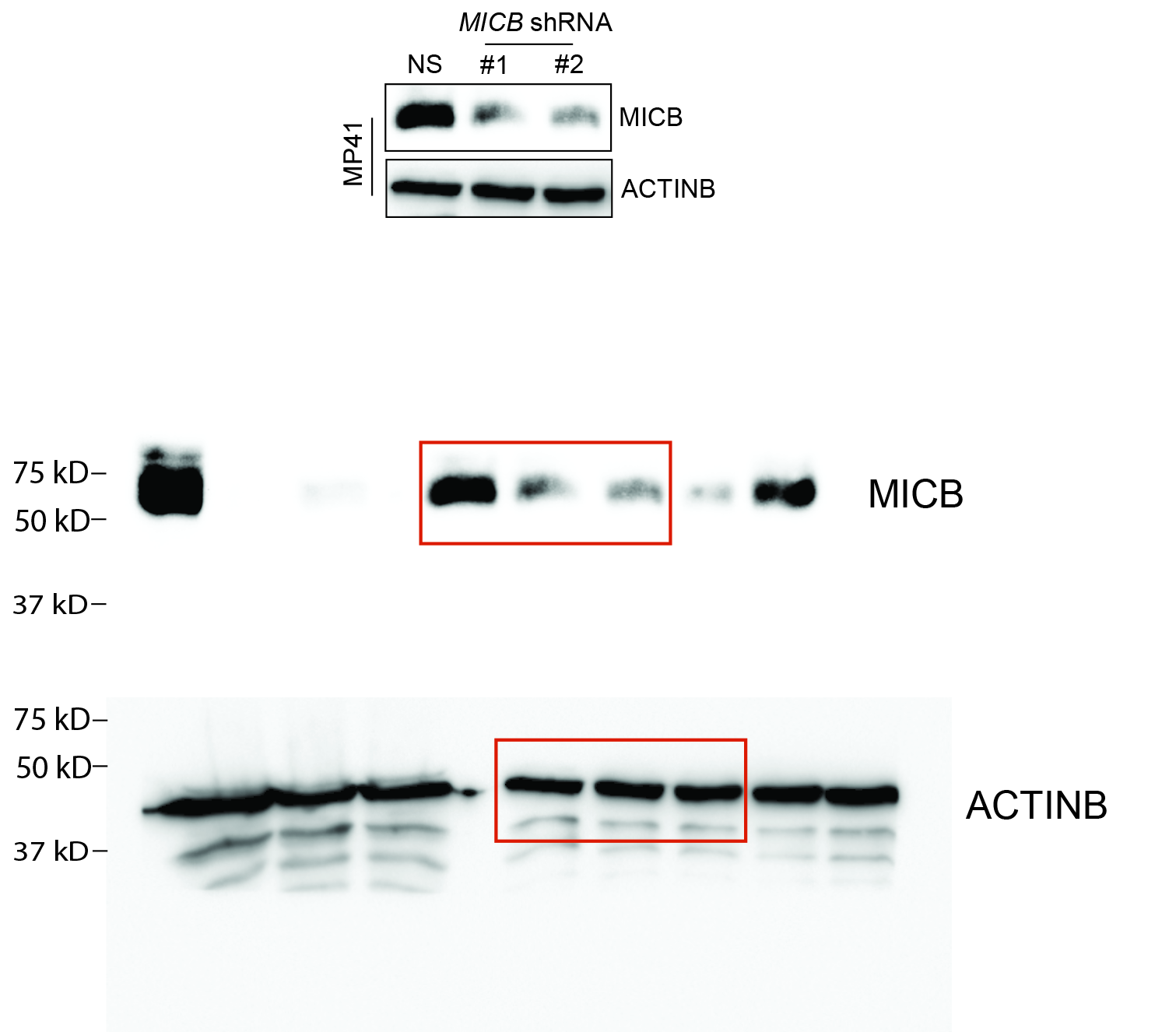

Supplement: Supplementary file 9 — Source data Fig. 4 [file 44321_2025_357_MOESM9_ESM.zip › Figure 4/4D/MICB shRNA panel blots/MP41/Figure 4D-MP41 MICB shRNA panel Western blots.tif]

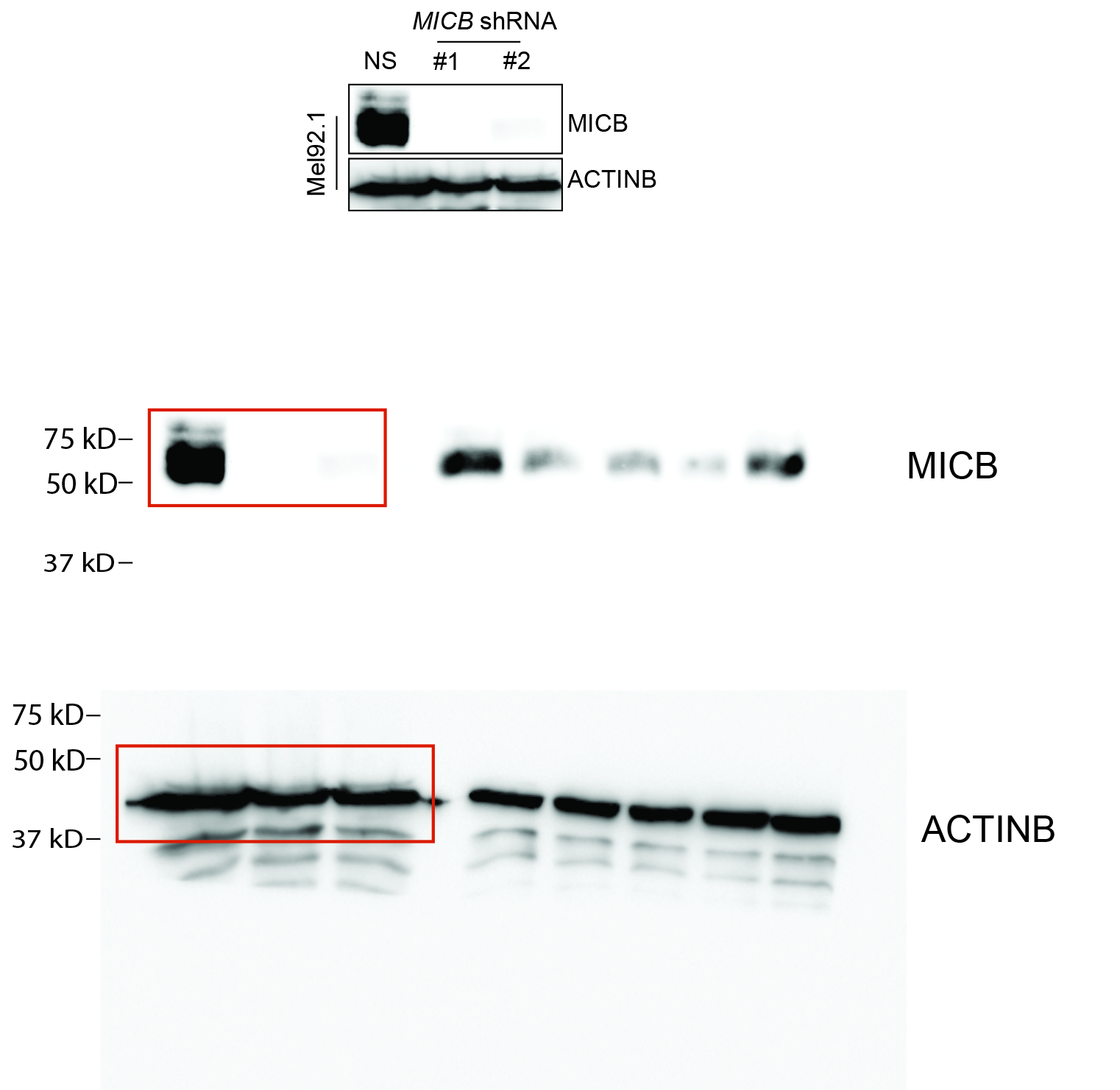

Supplement: Supplementary file 9 — Source data Fig. 4 [file 44321_2025_357_MOESM9_ESM.zip › Figure 4/4D/MICB shRNA panel blots/Mel92.1/Figure 4D-Mel92.1 MICB shRNA panel Western blots.tif]

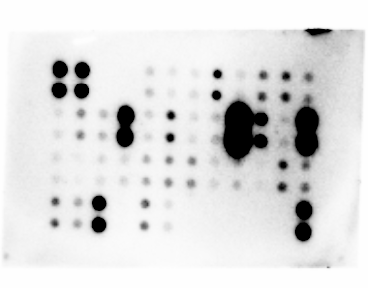

Supplement: Supplementary file 10 — Source data Fig. 5 [file 44321_2025_357_MOESM10_ESM.zip › Figure 5/5B/UNC0642 higher exposure.tif]

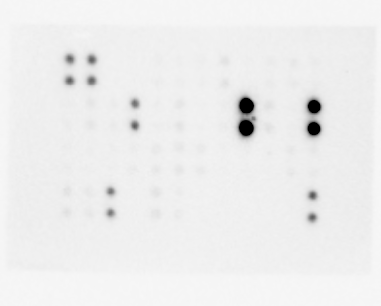

Supplement: Supplementary file 10 — Source data Fig. 5 [file 44321_2025_357_MOESM10_ESM.zip › Figure 5/5B/DMSO low exposure.tif]

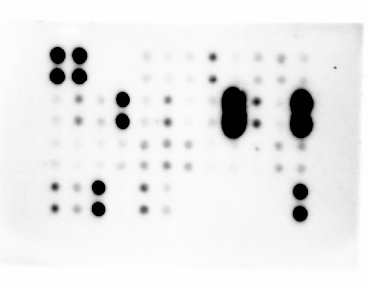

Supplement: Supplementary file 10 — Source data Fig. 5 [file 44321_2025_357_MOESM10_ESM.zip › Figure 5/5B/DMSO higher exposure.tif]

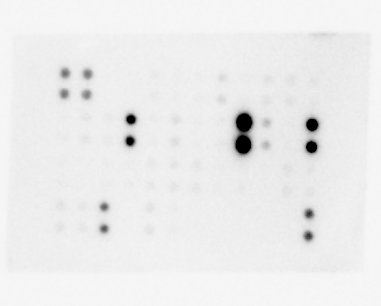

Supplement: Supplementary file 10 — Source data Fig. 5 [file 44321_2025_357_MOESM10_ESM.zip › Figure 5/5B/UNC0642 low exposure.tif]

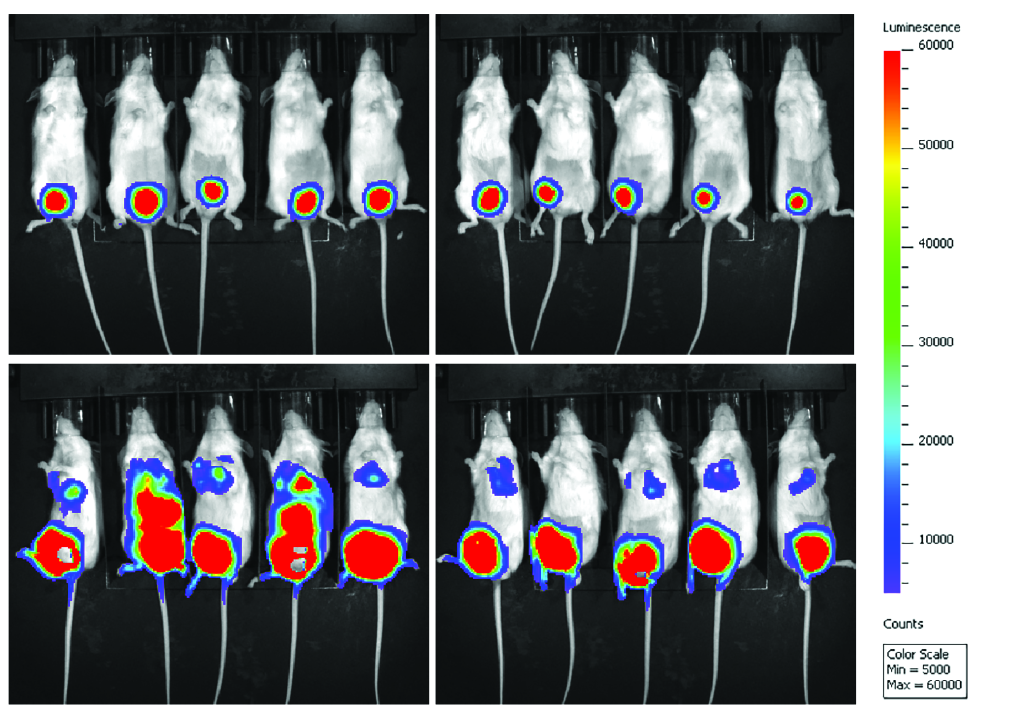

Supplement: Supplementary file 11 — Source data Fig. 6 [file 44321_2025_357_MOESM11_ESM.zip › Figure 6/6H/Figure 6H.tif]

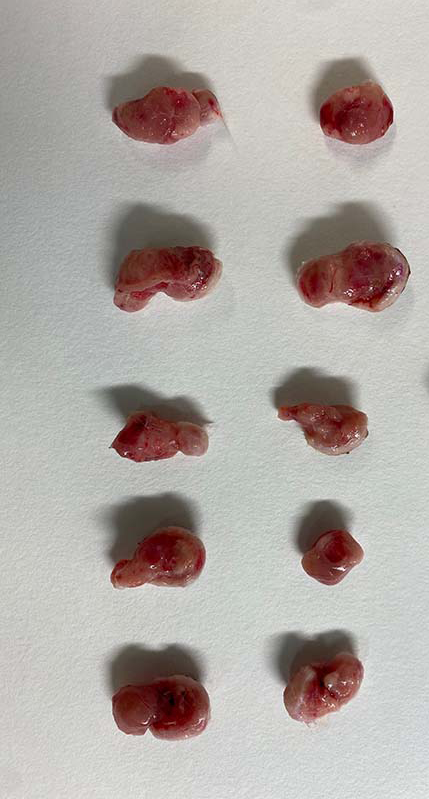

Supplement: Supplementary file 11 — Source data Fig. 6 [file 44321_2025_357_MOESM11_ESM.zip › Figure 6/6N/Figure 6N.tif]

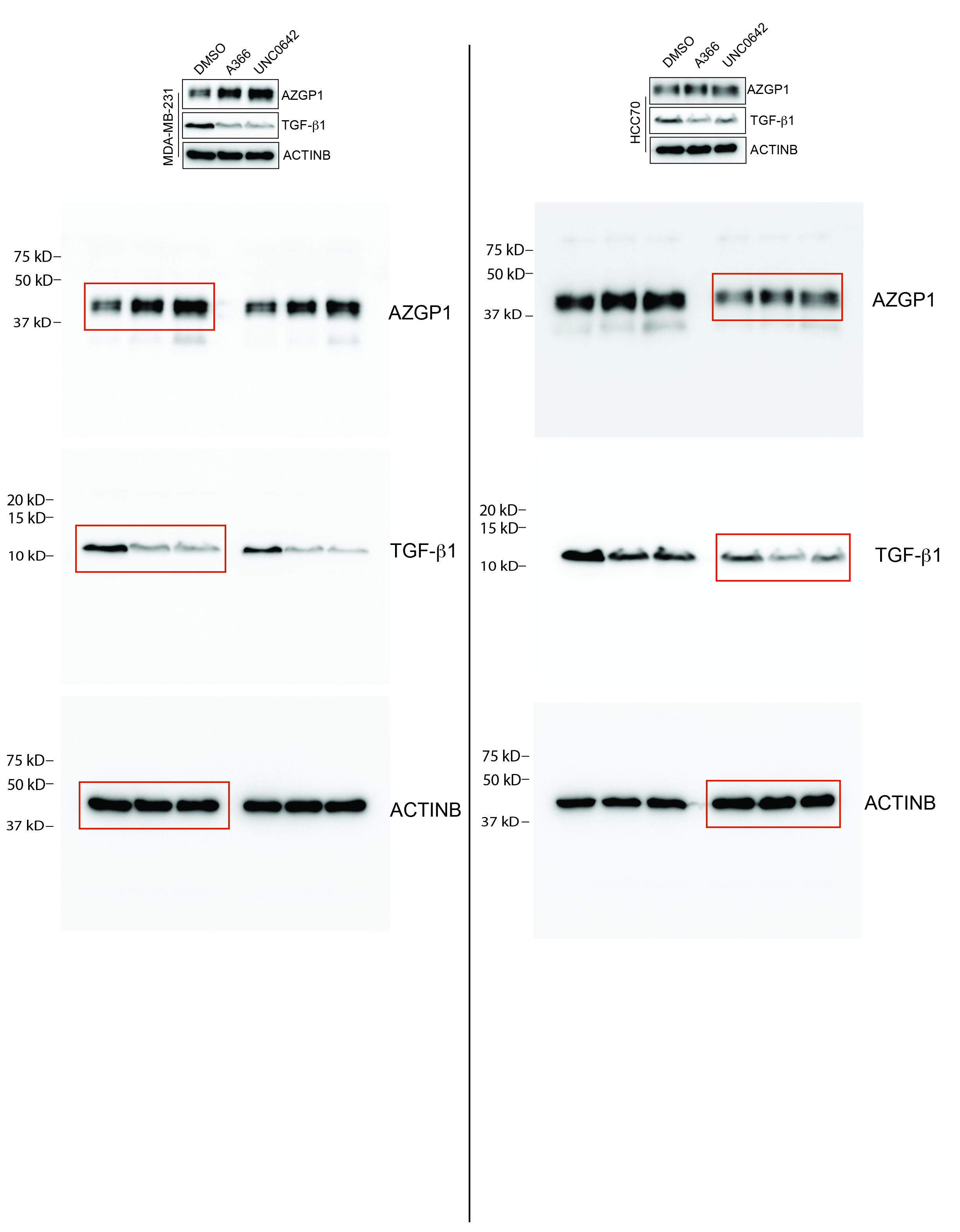

Supplement: Supplementary file 11 — Source data Fig. 6 [file 44321_2025_357_MOESM11_ESM.zip › Figure 6/6B/Figure 6B-MDA-MB-231 and HCC70 Western blots.tif]

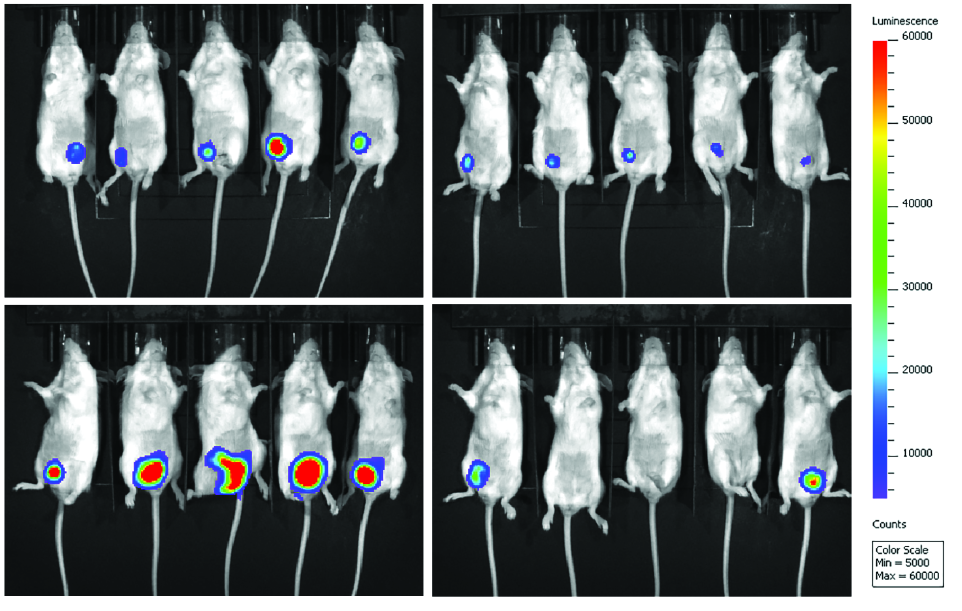

Supplement: Supplementary file 11 — Source data Fig. 6 [file 44321_2025_357_MOESM11_ESM.zip › Figure 6/6E/FIgure 6E.tif]

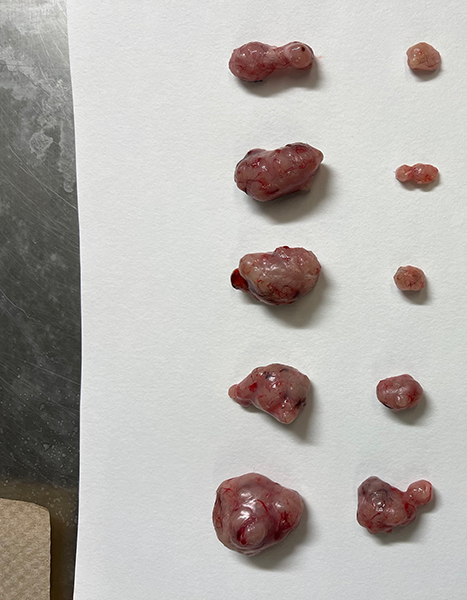

Supplement: Supplementary file 11 — Source data Fig. 6 [file 44321_2025_357_MOESM11_ESM.zip › Figure 6/6K/FIgure 6K.tif]

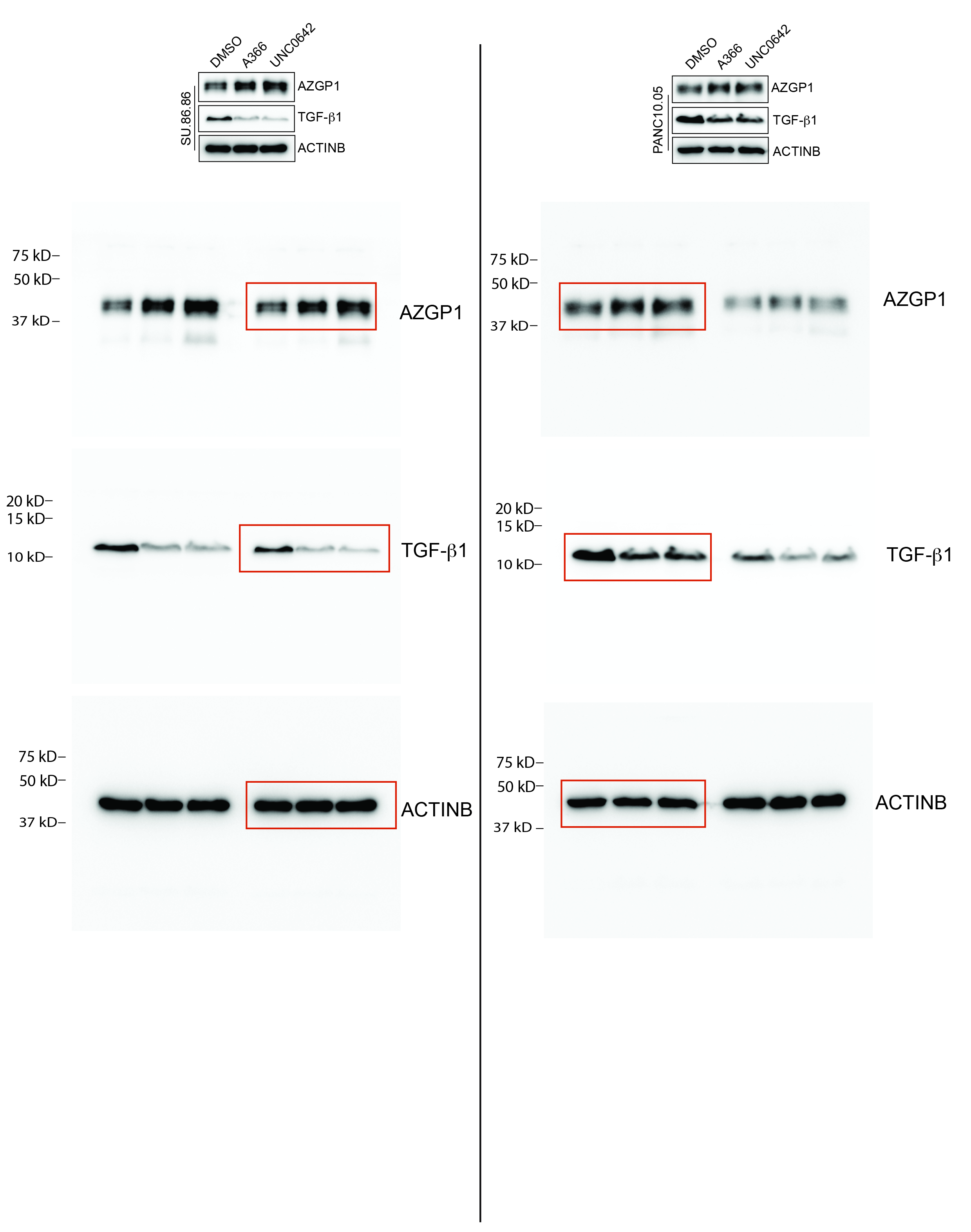

Supplement: Supplementary file 11 — Source data Fig. 6 [file 44321_2025_357_MOESM11_ESM.zip › Figure 6/6D/Figure 6D-SU.86.86 and PANC10.05 Western blots.tif]

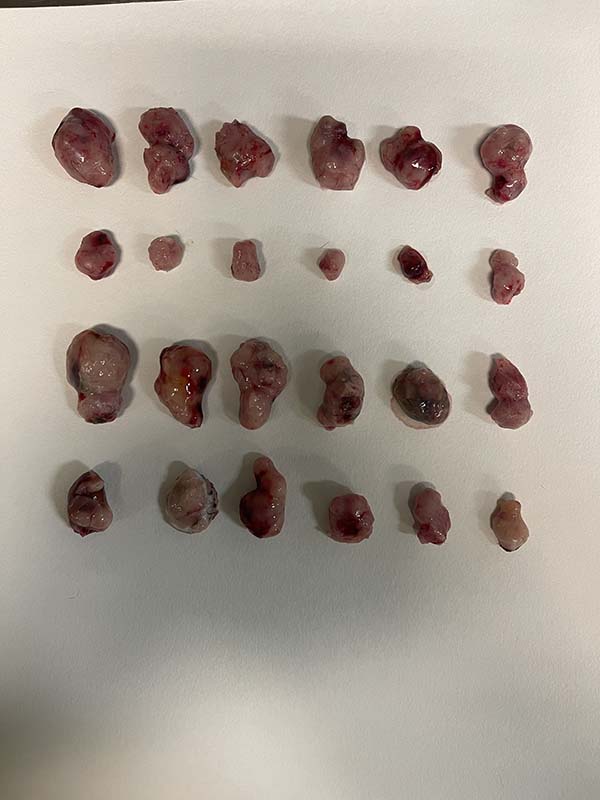

Supplement: Supplementary file 12 — Source data Fig. 7 [file 44321_2025_357_MOESM12_ESM.zip › Figure 7/7B/Figure 7B.JPG]

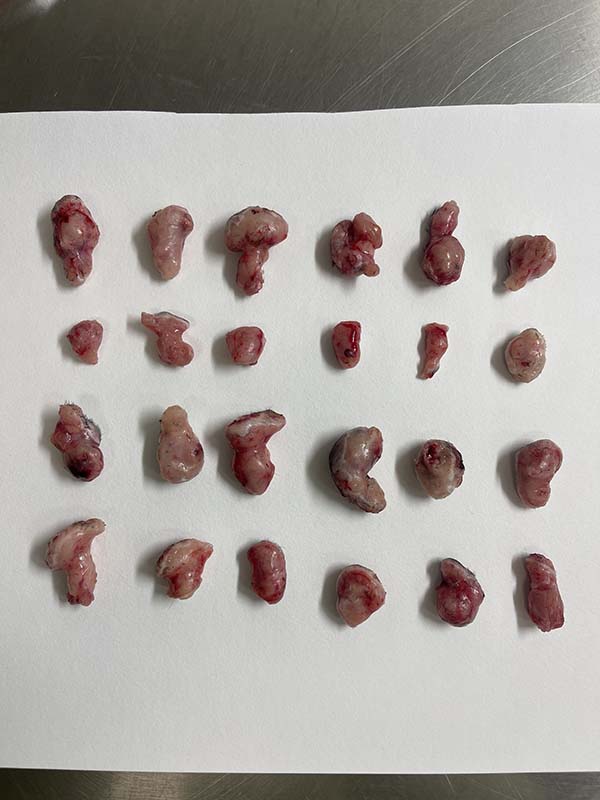

Supplement: Supplementary file 12 — Source data Fig. 7 [file 44321_2025_357_MOESM12_ESM.zip › Figure 7/7F/Figure 7F.jpeg]

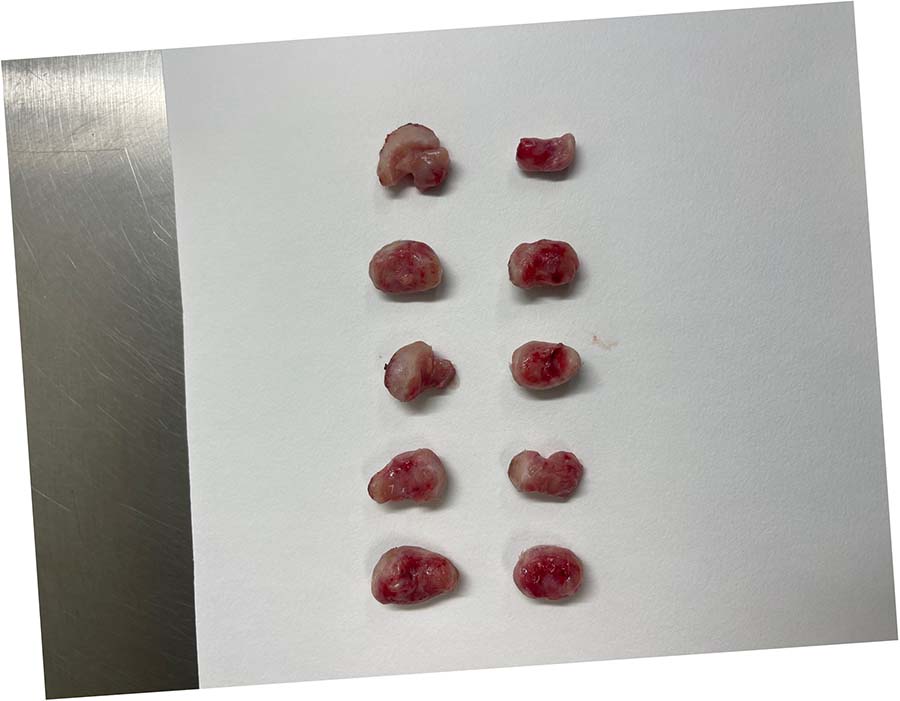

Supplement: Supplementary file 13 — Source data Fig. 8 [file 44321_2025_357_MOESM13_ESM.zip › Figure 8/8I/Figure 8I.jpg]

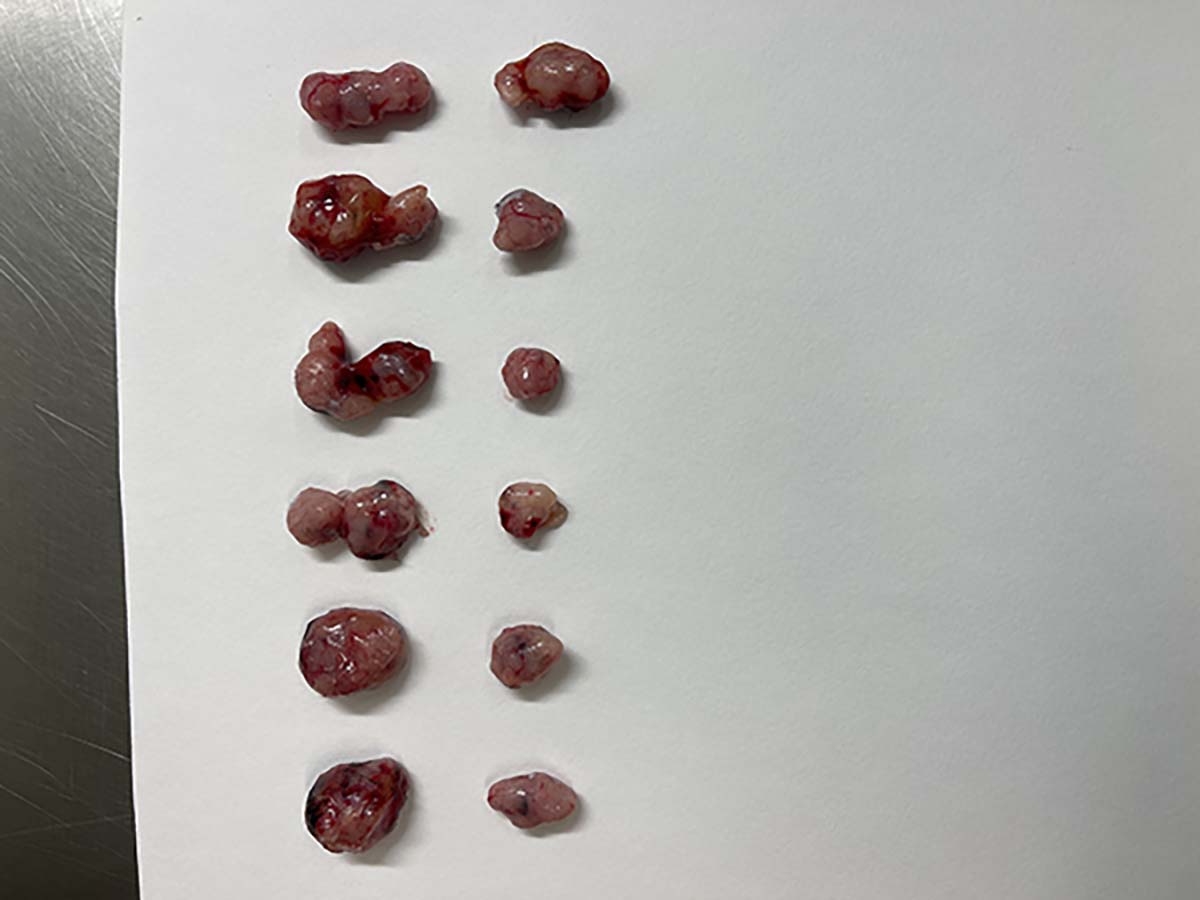

Supplement: Supplementary file 13 — Source data Fig. 8 [file 44321_2025_357_MOESM13_ESM.zip › Figure 8/8F/Figure 8F.jpeg]

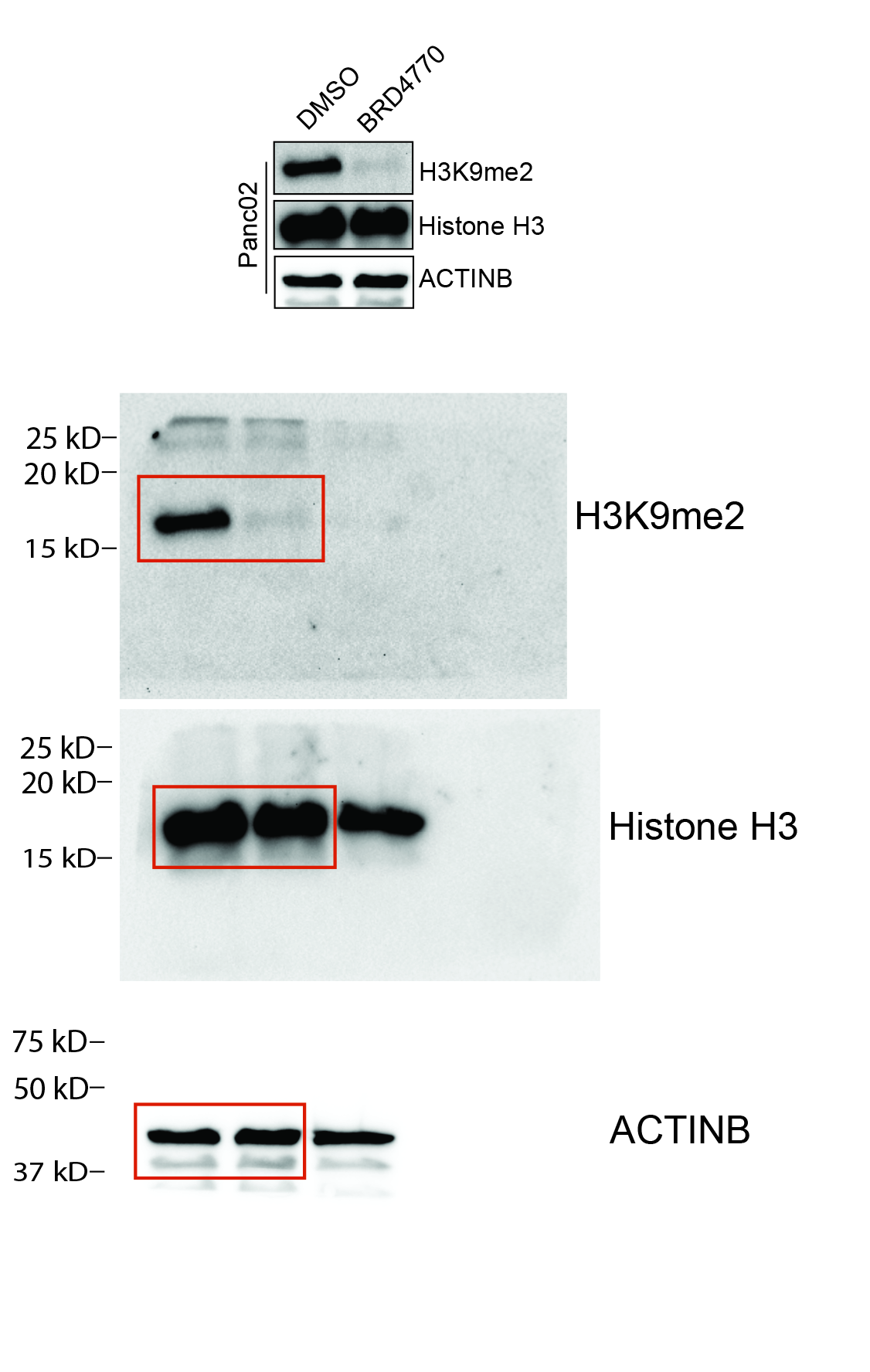

Supplement: Supplementary file 13 — Source data Fig. 8 [file 44321_2025_357_MOESM13_ESM.zip › Figure 8/8D/Figure 8D-Panc02 Western blot.tif]

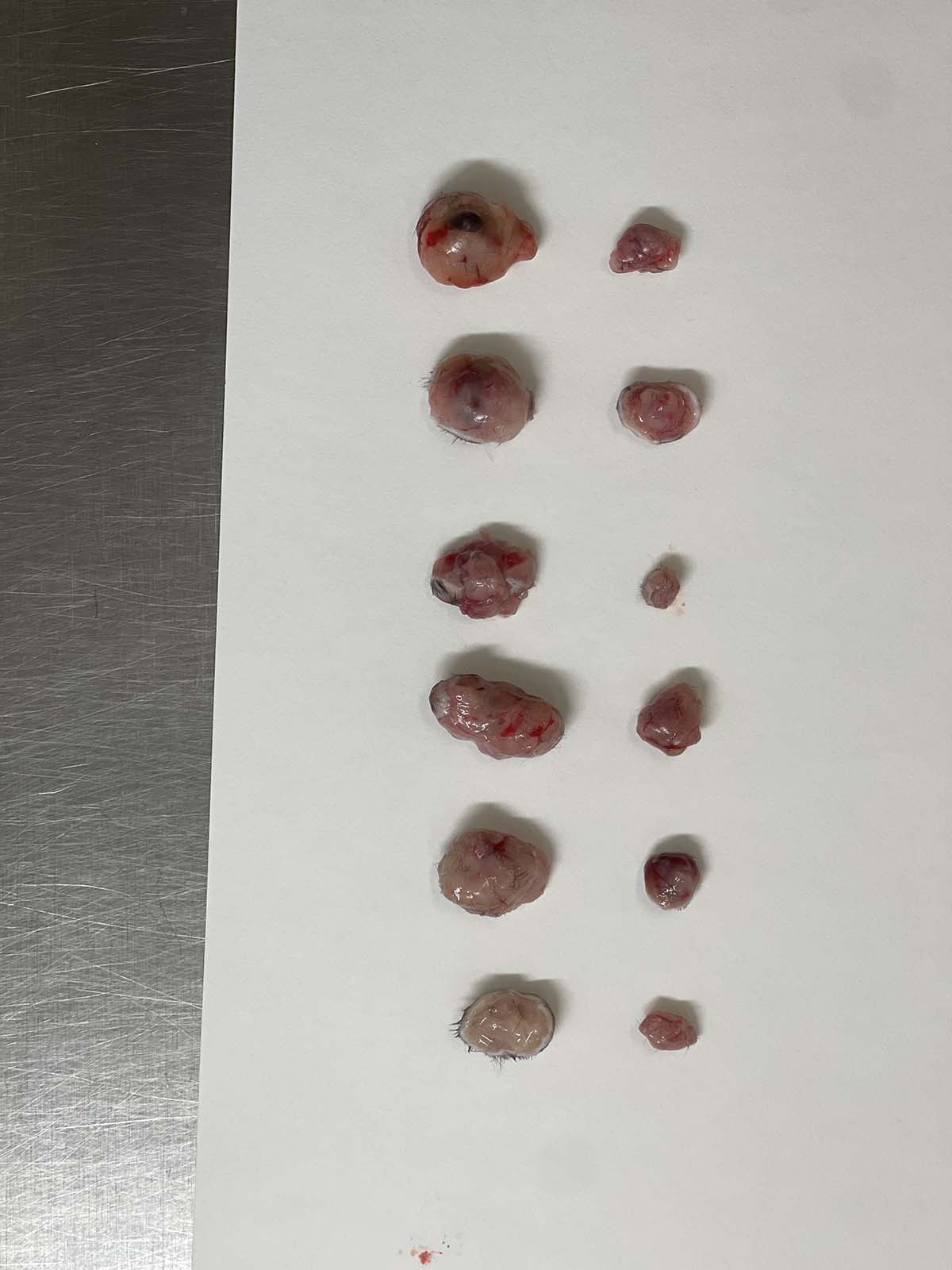

Supplement: Supplementary file 13 — Source data Fig. 8 [file 44321_2025_357_MOESM13_ESM.zip › Figure 8/8B/Figure 8B.jpeg]

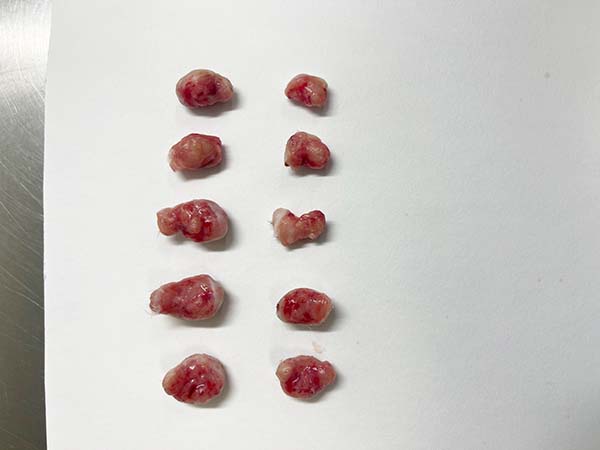

Supplement: Supplementary file 13 — Source data Fig. 8 [file 44321_2025_357_MOESM13_ESM.zip › Figure 8/8K/Figure 8K.jpg]
