# Supplementary material for: Loss of EHMT2 enhances NK cell-driven anti-tumor immunity through TGF-β1 suppression
Source: EMBO Mol Med. 2025 Dec 9;18(1):232–74. doi: 10.1038/s44321-025-00357-6 (PMC12808752; doi:10.1038/s44321-025-00357-6)
Supplement: Supplementary file 14 — Expanded View Figures [file 44321_2025_357_MOESM14_ESM.pdf]

# Expanded View Figures

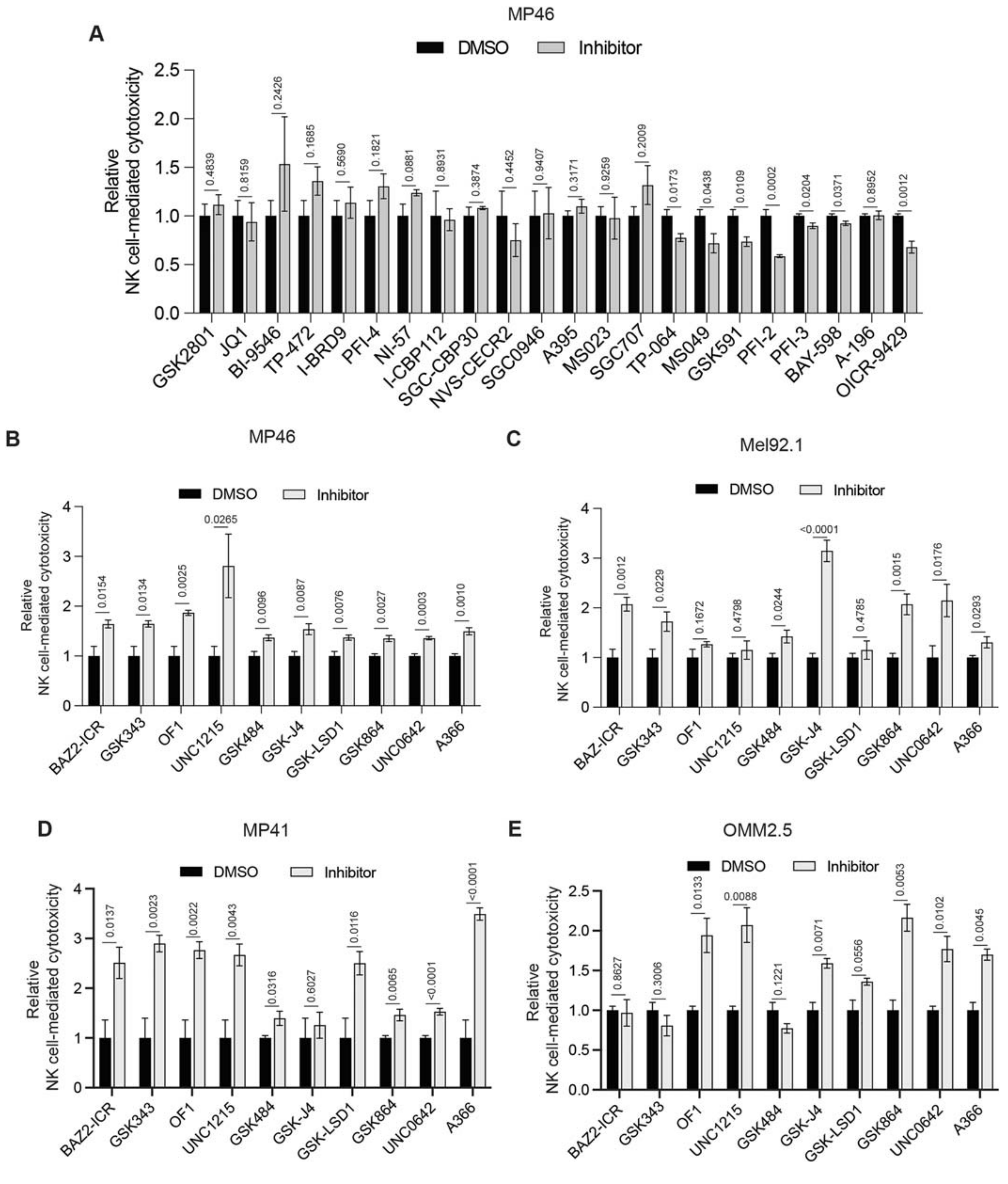

**Figure EV1. An epigenetic regulator inhibitor screen identifies suppressors of NK cell-mediated cytotoxicity.**

(**A**) MP46 cells were analyzed for NK cell-mediated cytotoxicity using an LDH-based cytotoxicity assay after treatment with DMSO or indicated small-molecule epigenetic inhibitors for 48 h. Relative NK cell-mediated cytotoxicity for the indicated inhibitors is plotted. ($n = 5$ for DMSO and $n = 3$ for BI-9546; $n = 4$ for DMSO and $n = 3$ for TP-472; $n = 4$ DMSO and $n = 4$ for I-BRD9, $n = 4$ PFI-1, $n = 4$ for I-CBP112, $n = 4$ for NVS-CECR2, $n = 4$ for SGC0946, $n = 4$ for MS023, $n = 4$ for SGC707; $n = 4$ DMSO and $n = 5$ A395; for all other sample DMSO and inhibitors conditions ($n = 5$ each). $P$ values were calculated using unpaired two-tailed Student's $t$-test. (**B**) MP46 cells were analyzed for NK cell-mediated cytotoxicity using an LDH-based cytotoxicity assay after treatment with DMSO or indicated small-molecule epigenetic inhibitors for 48 h. Relative NK cell-mediated cytotoxicity for the indicated inhibitors is plotted. ($n = 5$ for DMSO and BAZ2-ICR, $n = 5$ for GSK343, $n = 5$ for OF-1 and $n = 5$ UNC1215; for all other samples DMSO $n = 4$ and inhibitors $n = 5$)) $P$ values were calculated using unpaired two-tailed Student's $t$-test. (**C**) Mel92.1 cells were analyzed for NK cell-mediated cytotoxicity using an LDH-based cytotoxicity assay after treatment with indicated DMSO or small-molecule epigenetic inhibitors for 48 h. Relative NK cell-mediated cytotoxicity for the indicated inhibitors is plotted. ($n = 6$ for DMSO, $n = 6$ UNC0642 and $n = 6$ A366; for all other samples $n = 5$ for DMSO and $n = 5$ for inhibitors). $P$ values were calculated using unpaired two-tailed Student's $t$-test. (**D**) MP41 cells were analyzed for NK cell-mediated cytotoxicity using an LDH-based cytotoxicity assay after treatment with DMSO or indicated small-molecule epigenetic inhibitors for 48 h. Relative NK cell-mediated cytotoxicity for the indicated inhibitors is plotted. ($n = 6$ for DMSO, $n = 6$ UNC0642 and $n = 6$ A366; for all other samples $n = 5$ for DMSO and $n = 5$ for inhibitors). $P$ values were calculated using unpaired two-tailed Student's $t$-test. (**E**) OMM2.5 cells were analyzed for NK cell-mediated cytotoxicity using an LDH-based cytotoxicity assay after treatment with indicated DMSO or small-molecule epigenetic inhibitors for 48 h. Relative NK cell-mediated cytotoxicity for the indicated inhibitors is plotted. ($n = 3$ for DMSO and $n = 3$ for inhibitors). $P$ values were calculated using unpaired two-tailed Student's $t$-test. All quantitative data were shown as the mean ± SEM.

 © The Author(s)

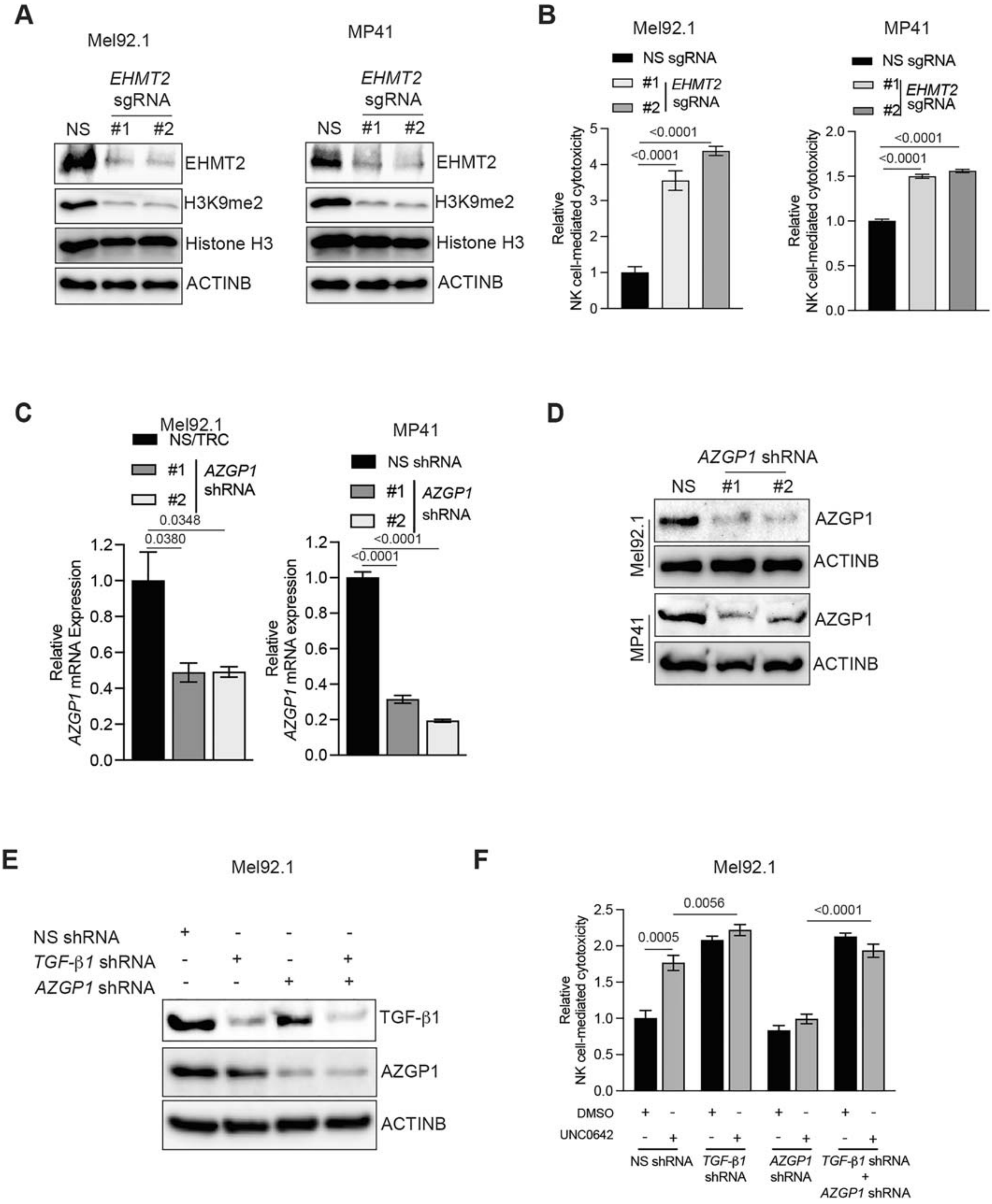
A
Mel92.1
EHMT2 sgRNA
NS #1 #2
EHMT2
H3K9me2
Histone H3
ACTINB
MP41
B
NS sgRNA
#1 #2 EHMT2 sgRNA
Relative NK cell-mediated cytotoxicity
<0.0001
C
NS/TRC
#1 #2 AZGP1 shRNA
0.0348
0.0380
Relative AZGP1 mRNA Expression
NS shRNA
Relative AZGP1 mRNA expression
D
AZGP1 shRNA
AZGP1
E
TGF-β1 shRNA
AZGP1 shRNA
TGF-β1
F
0.0056
0.0005
DMSO
UNC0642
NS shRNA
TGF-β1 shRNA
AZGP1 shRNA
TGF-β1 shRNA + AZGP1 shRNA

**Figure EV2. Analysis of EHMT2 knockout cancer cells in NK cell-mediated cytotoxicity assay and validation of AZGP1 knockdown.**

(**A**) Mel92.1 and MP41 cells expressing either nonspecific single-guide RNA (NS sgRNA) or *EHMT2* sgRNAs were analyzed for the indicated proteins using immunoblotting. Histone H3 and ACTINB were used as loading controls. (**B**) Mel92.1 and MP41 cells expressing either NS sgRNA or *EHMT2* sgRNAs were analyzed for NK cell-mediated cytotoxicity using an LDH-based cytotoxicity assay. Relative NK cell-mediated cytotoxicity under indicated conditions are plotted. ($n = 6$). *P* values were calculated using unpaired two-tailed Student's *t*-test. (**C**) Mel92.1 and MP41 cells expressing either nonspecific (NS) shRNA or *AZGP1* shRNAs were analyzed for mRNA expression for *AZGP1* mRNA were analyzed using RT-qPCR analysis. Relative mRNA expression for indicated genes are plotted. *ACTINB* was used for normalization. ($n = 3$). *P* values were calculated using unpaired two-tailed Student's *t*-test. (**D**) Mel92.1 and MP41 cells expressing either NS shRNA or *AZGP1* shRNAs were analyzed for the expression of AZGP1 by immunoblotting. ACTINB was used as a loading control. (**E**) Mel92.1 cells expressing nonspecific NS shRNA, *AZGP1* shRNA, *TGF-β1* shRNA or both *AZGP1* shRNA and *TGF-β1* shRNA were analyzed by immunoblotting for the indicated proteins. ACTINB was used as a loading control. (**F**) Mel92.1 cells expressing either NS shRNA, *AZGP1* shRNA, *TGF-β1* shRNA or both *AZGP1* shRNA and *TGF-β1* shRNA were treated with DMSO or EHMT2 inhibitor UNC0642 (1 μM) for 48 h and were analyzed for NK cell-mediated cytotoxicity using an LDH-based cytotoxicity assay. Relative NK cell-mediated cytotoxicity under the indicated conditions is plotted. ($n = 6$). *P* values were calculated using unpaired two-tailed Student's *t*-test. All quantitative data were presented as the mean ± SEM.

 © The Author(s)

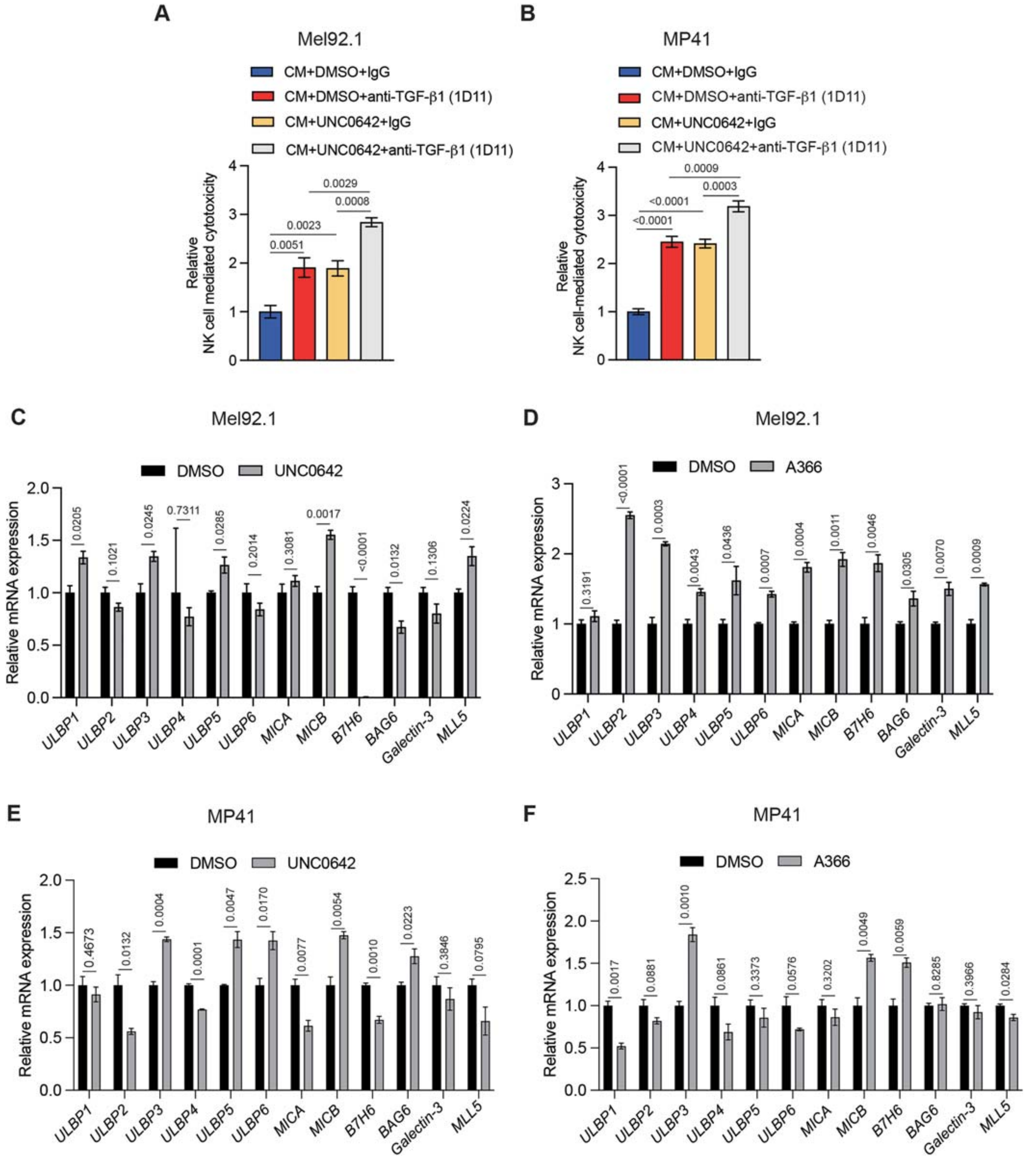
A
Mel92.1
CM+DMSO+IgG
CM+DMSO+anti-TGF-β1 (1D11)
CM+UNC0642+IgG
CM+UNC0642+anti-TGF-β1 (1D11)
Relative NK cell mediated cytotoxicity
B
MP41
Relative NK cell-mediated cytotoxicity
C
Mel92.1
DMSO
UNC0642
Relative mRNA expression
D
A366
E
MP41
F
ULBP1
ULBP2
ULBP3
ULBP4
ULBP5
ULBP6
MICA
MICB
B7H6
BAG6
Galectin-3
MLL5

**Figure EV3. Simultaneous inhibition of EHMT2 and neutralization of TGF-β1 augments NK cell-mediated cytotoxicity, and analysis of NK cell ligands in UM cell lines following EHMT2 inhibition.**

(**A**) Mel92.1 cells were treated with the EHMT2 inhibitor UNC0642 (1 μM) or DMSO for 72 h in Opti-MEM. Following this, conditioned media (CM) was then collected, concentrated, and used to treat NK cells for 24 h in the presence of either control IgG or a TGF-β1-neutralizing antibody (1D11). NK cells were then assessed for cytotoxic activity against Mel92.1 cells using an LDH-based cytotoxicity assay. The relative NK cell-mediated cytotoxicity under the indicated conditions is shown. ($n = 5$). *P* values were calculated using unpaired two-tailed Student's *t*-test. (**B**) MP41 cells were treated with the EHMT2 inhibitor UNC0642 (1 μM) or DMSO for 72 h in Opti-MEM. Following this, conditioned media (CM) was then collected, concentrated, and used to treat NK cells for 24 h in the presence of either control IgG or a TGF-β1-neutralizing antibody (1D11). NK cells were then assessed for cytotoxic activity against MP41 cells using an LDH-based cytotoxicity assay. The relative NK cell-mediated cytotoxicity under the indicated conditions is shown. ($n = 6$). *P* values were calculated using unpaired two-tailed Student's *t*-test. (**C**) Mel92.1 cells treated with DMSO or UNC0642 (3 μM) for 48 h were analyzed for the indicated mRNAs using RT-qPCR. mRNA expression relative to DMSO-treated cells is plotted. *ACTINB* was used for normalization. ($n = 3$). *P* values were calculated using unpaired two-tailed Student's *t*-test. (**D**) Mel92.1 cells treated with DMSO or A366 (3 μM) for 48 h were analyzed for the indicated mRNAs using RT-qPCR. mRNA expression relative to DMSO-treated cells is plotted. *ACTINB* was used for normalization. ($n = 3$). *P* values were calculated using unpaired two-tailed Student's *t*-test. (**E**) MP41 cells treated with DMSO or UNC0642 (3 μM) for 48 h were analyzed for the indicated mRNAs using RT-qPCR. mRNA expression relative to DMSO-treated cells is plotted. *ACTINB* was used for normalization. ($n = 3$). *P* values were calculated using unpaired two-tailed Student's *t*-test. (**F**) MP41 cells treated with DMSO or A366 (3 μM) for 48 h were analyzed for the indicated mRNAs using RT-qPCR. mRNA expression relative to DMSO-treated cells is plotted. *ACTINB* was used for normalization. ($n = 3$). *P* values were calculated using unpaired two-tailed Student's *t*-test. All quantitative data were presented as mean ± SEM.

 © The Author(s)

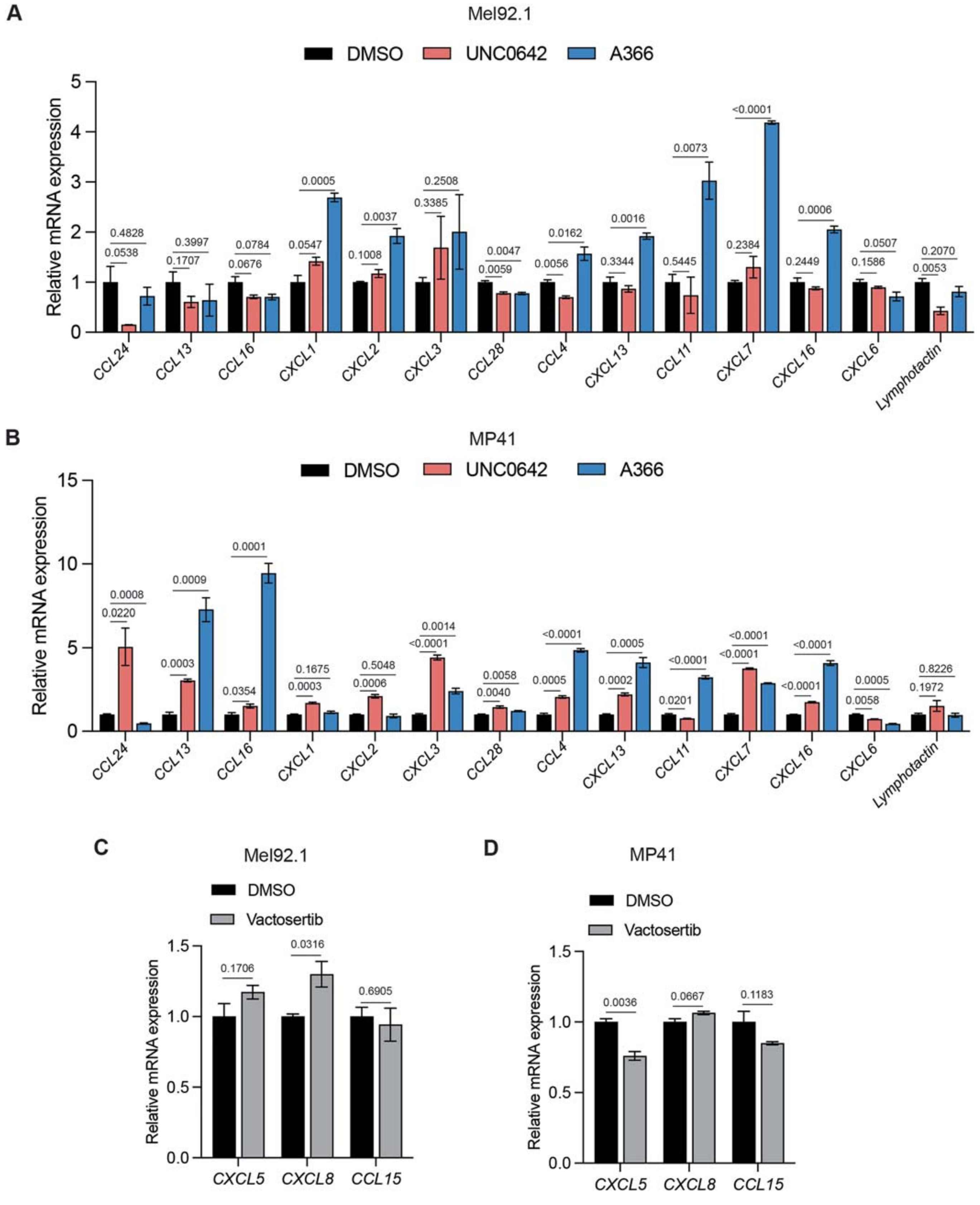
A
Mel92.1
DMSO
UNC0642
A366
Relative mRNA expression
0.4828
0.0538
0.3997
0.1707
0.0784
0.0676
0.0005
0.0547
0.0037
0.1008
0.2508
0.3385
0.0047
0.0059
0.0162
0.0056
0.0016
0.3344
0.0073
0.5445
<0.0001
0.2384
0.0006
0.2449
0.0507
0.1586
0.2070
0.0053
CCL24
CCL13
CCL16
CXCL1
CXCL2
CXCL3
CCL28
CCL4
CXCL13
CCL11
CXCL7
CXCL16
CXCL6
Lymphotactin
B
MP41
DMSO
UNC0642
A366
Relative mRNA expression
0.0008
0.0220
0.0009
0.0003
0.0001
0.0354
0.1675
0.0003
0.5048
0.0006
0.0014
<0.0001
0.0058
0.0040
<0.0001
0.0005
0.0005
0.0002
<0.0001
0.0201
<0.0001
<0.0001
<0.0001
<0.0001
0.0005
0.0058
0.8226
0.1972
CCL24
CCL13
CCL16
CXCL1
CXCL2
CXCL3
CCL28
CCL4
CXCL13
CCL11
CXCL7
CXCL16
CXCL6
Lymphotactin
C
Mel92.1
DMSO
Vactosertib
Relative mRNA expression
0.1706
0.0316
0.6905
CXCL5
CXCL8
CCL15
D
MP41
DMSO
Vactosertib
Relative mRNA expression
0.0036
0.0667
0.1183
CXCL5
CXCL8
CCL15

© The Author(s)

**Figure EV4. Analysis of human chemokines expression in uveal melanoma cell lines following EHMT2 inhibitor treatment.**

(**A**) Mel92.1 cells treated with DMSO, or UNC0642 (1 μM), A366 (1 μM) for 72 h were analyzed for indicated mRNAs using RT-qPCR. mRNA expression relative to DMSO-treated cells is plotted. *ACTINB* was used for normalization. ($n = 3$). *P* values were calculated using unpaired two-tailed Student's *t*-test. (**B**) MP41 cells treated with DMSO, or UNC0642 (1 μM), A366 (1 μM) for 72 h were analyzed for indicated mRNAs using RT-qPCR. mRNA expression relative to DMSO-treated cells is plotted. *ACTINB* was used for normalization. ($n = 3$). *P* values were calculated using unpaired two-tailed Student's *t*-test. (**C**) Mel92.1 cells treated with vactosertib (1 μM) for 48 h were analyzed for indicated mRNAs using RT-qPCR. mRNA expression relative to DMSO-treated cells is plotted. *ACTINB* was used for normalization. ($n = 3$). *P* values were calculated using unpaired two-tailed Student's *t*-test. (**D**) MP41 cells were treated with vacotosertib (1 μM) for 48 h were analyzed for the indicated mRNAs using RT-qPCR. mRNA expression relative to DMSO-treated cells is plotted. *ACTINB* was used for normalization. ($n = 3$). *P* values were calculated using unpaired two-tailed Student's *t*-test. All quantitative data were shown as the mean ± SEM.

 © The Author(s)

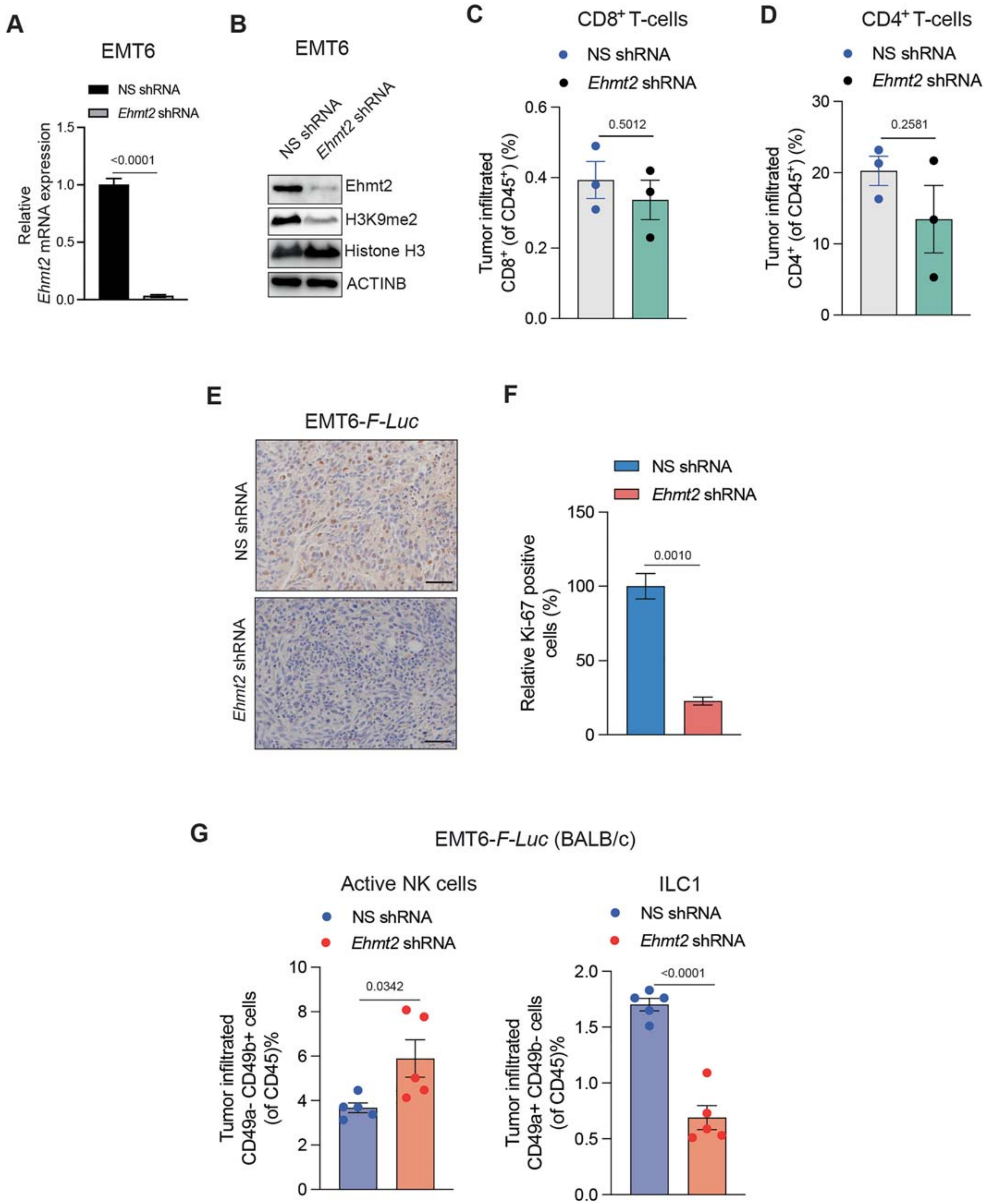
A
EMT6
NS shRNA
Ehmt2 shRNA
Relative Ehmt2 mRNA expression
<0.0001
B
EMT6
NS shRNA
Ehmt2 shRNA
Ehmt2
H3K9me2
Histone H3
ACTINB
C
CD8+ T-cells
NS shRNA
Ehmt2 shRNA
Tumor infiltrated CD8+ (of CD45+) (%)
0.5012
D
CD4+ T-cells
NS shRNA
Ehmt2 shRNA
Tumor infiltrated CD4+ (of CD45+) (%)
0.2581
E
EMT6-F-Luc
NS shRNA
Ehmt2 shRNA
F
NS shRNA
Ehmt2 shRNA
Relative Ki-67 positive cells (%)
0.0010
G
EMT6-F-Luc (BALB/c)
Active NK cells
NS shRNA
Ehmt2 shRNA
Tumor infiltrated CD49a- CD49b+ cells (of CD45)%
0.0342
ILC1
NS shRNA
Ehmt2 shRNA
Tumor infiltrated CD49a+ CD49b- cells (of CD45)%
<0.0001

**Figure EV5. Analysis of EMT6 tumors.**

(**A**) EMT6 cells expressing either nonspecific (NS) shRNA or *Ehmt2* shRNA were analyzed for mRNA expression for *Ehmt2* mRNA were analyzed using RT-qPCR analysis. Relative mRNA expression is plotted as the mean ± SEM. *Actinb* was used for normalization. ($n = 3$). *P* values were calculated using unpaired two-tailed Student's *t*-test. (**B**) EMT6 cells expressing either NS shRNA or *Ehmt2* shRNA were analyzed for the expression of the indicated proteins by immunoblotting. Histone H3 and ACTINB were used as loading controls. (**C**) Firefly luciferase (*F-Luc*)-labeled EMT6 cells expressing either NS shRNA or *Ehmt2* shRNA were orthotopically injected into the mammary fat pad of female BALB/c mice. Measurement of tumor-infiltered $CD8^+$ T-cells (%) in the EMT6 tumors under indicated conditions using FACS analysis. ($n = 3$). *P* values were calculated using unpaired two-tailed Student's *t*-test. (**D**) Firefly luciferase (*F-Luc*)-labeled EMT6 cells expressing either NS shRNA or *Ehmt2* shRNA were orthotopically injected into the mammary fat pad of female BALB/c mice. Measurement of tumor-infiltered $CD4^+$ T-cells (%) in the EMT6 tumors under indicated conditions using FACS analysis and plotted. ($n = 3$). *P* values were calculated using unpaired two-tailed Student's *t*-test. (**E**) Ki-67 expression was analyzed by immunohistochemistry (IHC) in EMT6 tumor sections expressing NS shRNA and *Ehmt2* shRNA. Representative Ki-67 staining images of EMT6 tumor sections expressing NS shRNA and *Ehmt2* shRNA at 20× magnifications are shown. Scale bar, 50 µm. (**F**) Quantitation of Ki-67 staining for the experiment presented in panel (**E**). ($n = 3$). *P* values were calculated using unpaired two-tailed Student's *t*-test. (**G**) EMT6 cells expressing NS shRNA or *Ehmt2* shRNAs were injected subcutaneously into BALB/c mice. Measurement of tumor-infiltrated NK cells ($Lin^-CD49a^-CD49b^+$) and ILC1 ($Lin^-CD49a^+CD49b^-$) in the EMT6 tumors under the indicated conditions using FACS analysis and plotted. ($n = 5$). *P* values were calculated using unpaired two-tailed Student's *t*-test. All quantitative data were shown as the mean ± SEM.

 © The Author(s)

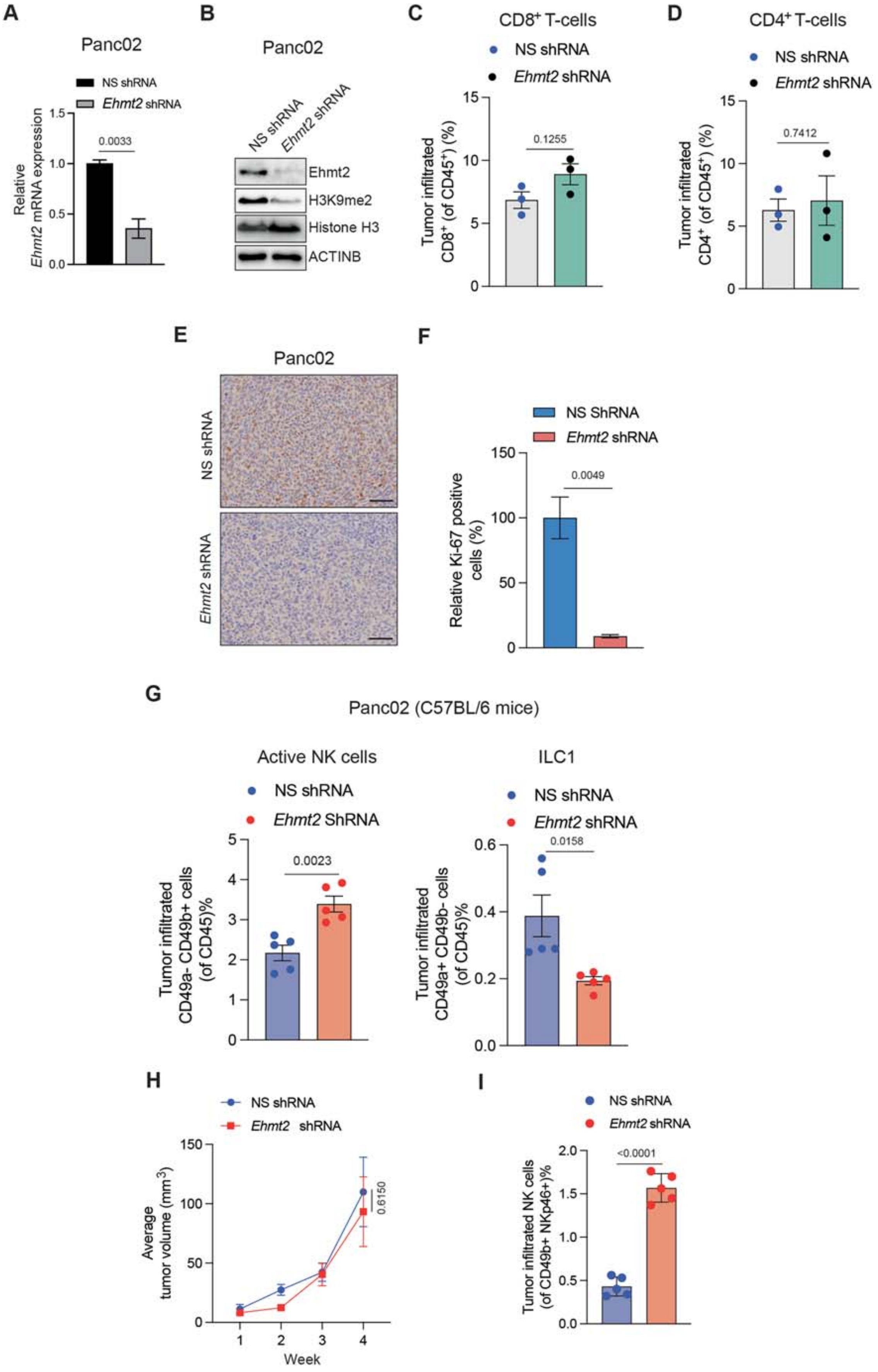
A
Panc02
NS shRNA
Ehmt2 shRNA
Relative Ehmt2 mRNA expression
0.0033
B
Panc02
NS shRNA
Ehmt2 shRNA
Ehmt2
H3K9me2
Histone H3
ACTINB
C
CD8+ T-cells
NS shRNA
Ehmt2 shRNA
Tumor infiltrated CD8+ (of CD45+) (%)
0.1255
D
CD4+ T-cells
NS shRNA
Ehmt2 shRNA
Tumor infiltrated CD4+ (of CD45+) (%)
0.7412
E
Panc02
NS shRNA
Ehmt2 shRNA
F
NS ShRNA
Ehmt2 shRNA
Relative Ki-67 positive cells (%)
0.0049
G
Panc02 (C57BL/6 mice)
Active NK cells
NS shRNA
Ehmt2 ShRNA
Tumor infiltrated CD49a- CD49b+ cells (of CD45)%
0.0023
ILC1
NS shRNA
Ehmt2 shRNA
Tumor infiltrated CD49a+ CD49b- cells (of CD45)%
0.0158
H
NS shRNA
Ehmt2 shRNA
Average tumor volume (mm3)
0.6150
Week
I
NS shRNA
Ehmt2 shRNA
Tumor infiltrated NK cells (of CD49b+ NKp46+)%
<0.0001

© The Author(s)

**Figure EV6. Analysis of Panc02 tumors.**

(**A**) Panc02 cells expressing either nonspecific (NS) shRNA or *Ehmt2* shRNA were analyzed for mRNA expression for *Ehmt2* mRNA were analyzed using RT-qPCR analysis. Relative mRNA expression is plotted. *Actinb* was used for normalization. ($n = 3$). *P* values were calculated using unpaired two-tailed Student's *t*-test. (**B**) Panc02 cells expressing either NS shRNA or *Ehmt2* shRNA were analyzed for the expression of the indicated proteins by immunoblotting. Histone H3 and ACTINB were used as loading controls. (**C**) Panc02 cells expressing NS shRNA or *Ehmt2* shRNAs were injected subcutaneously into C57BL/6 mice. Measurement of tumor-infiltered $CD8^+$ T-cells (%) in the Panc02 tumors under indicated conditions using FACS analysis and plotted. ($n = 3$). *P* values were calculated using unpaired two-tailed Student's t-test. (**D**) Panc02 cells expressing NS shRNA or *Ehmt2* shRNAs were injected subcutaneously into C57BL/6 mice. Measurement of tumor-infiltered $CD4^+$ T-cells cells (%) in the Panc02 tumors under indicated conditions using FACS analysis and plotted. ($n = 3$). *P* values were calculated using unpaired two-tailed Student's *t*-test. (**E**) Ki-67 expression was analyzed by immunohistochemistry (IHC) in Panc02 tumor sections expressing NS shRNA and *Ehmt2* shRNA. Representative Ki-67 staining images of Panc02 tumor sections expressing NS shRNA and *Ehmt2* shRNA at 20× magnifications are shown. Scale bar, 50 µm. (**F**) Quantitation of Ki-67 staining for the experiment presented in panel (**E**) and plotted. ($n = 3$). *P* values were calculated using unpaired two-tailed Student's *t*-test. (**G**) Panc02 cells expressing NS shRNA or *Ehmt2* shRNAs were injected subcutaneously into C57BL/6 mice. Measurement of tumor-infiltrated NK cells ($Lin^-CD49a^-CD49b^+$), and ILC1 ($Lin^-CD49a^+CD49b^-$) in the Panc02 tumors under the indicated conditions using FACS analysis and plotted. ($n = 5$). *P* values were calculated using unpaired two-tailed Student's *t*-test. (**H**) Panc02 cells expressing either NS shRNA or *Ehmt2* shRNA were injected subcutaneously into C57BL/6 mice. Tumor volumes at the indicated times are plotted. ($n = 5$). For the analysis of tumor progression in mice, the statistical assessment was performed using the area under the curve method, followed by unpaired two-tailed Student's *t*-tests. (**I**) Measurement of tumor-infiltrated NK cells (%) in the Panc02 tumors under the indicated conditions using FACS analysis and plotted. ($n = 5$). *P* values were calculated using unpaired two-tailed Student's *t*-test. All quantitative data were shown as the mean ± SEM.

 © The Author(s)

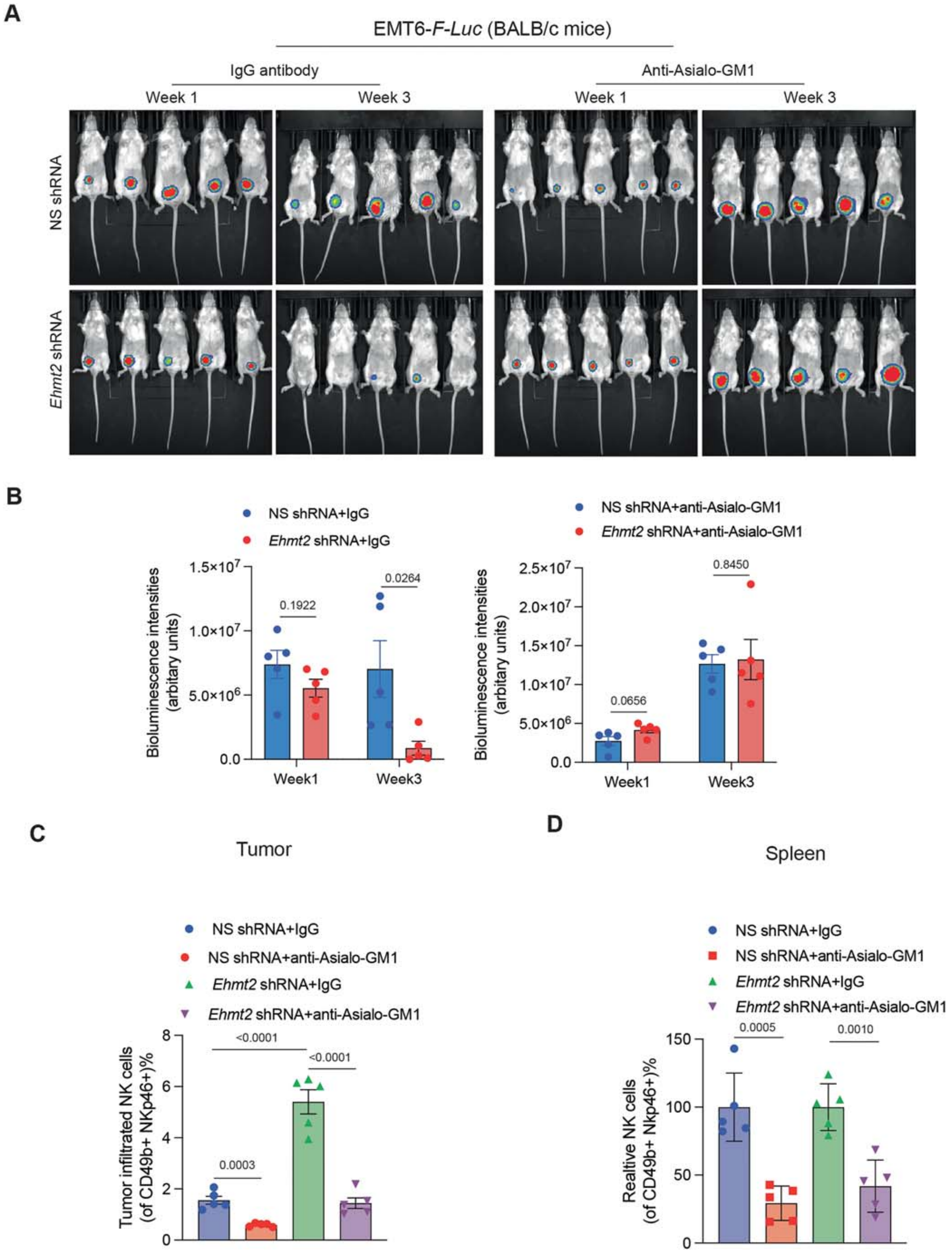
A
EMT6-F-Luc (BALB/c mice)
IgG antibody
Anti-Asialo-GM1
Week 1
Week 3
Week 1
Week 3
NS shRNA
Ehmt2 shRNA
B
NS shRNA+IgG
Ehmt2 shRNA+IgG
Bioluminescence intensities (arbitary units)
1.5×10^7
1.0×10^7
5.0×10^6
0.0
0.1922
0.0264
Week1
Week3
NS shRNA+anti-Asialo-GM1
Ehmt2 shRNA+anti-Asialo-GM1
2.5×10^7
2.0×10^7
1.5×10^7
1.0×10^7
5.0×10^6
0.0
0.0656
0.8450
C
Tumor
NS shRNA+IgG
NS shRNA+anti-Asialo-GM1
Ehmt2 shRNA+IgG
Ehmt2 shRNA+anti-Asialo-GM1
Tumor infiltrated NK cells (of CD49b+ NKp46+)%
8
6
4
2
0
<0.0001
<0.0001
0.0003
D
Spleen
NS shRNA+IgG
NS shRNA+anti-Asialo-GM1
Ehmt2 shRNA+IgG
Ehmt2 shRNA+anti-Asialo-GM1
Realtive NK cells (of CD49b+ Nkp46+)%
150
100
50
0
0.0005
0.0010

◀ **Figure EV7. NK cells are necessary for *Ehmt2* knockdown-mediated tumor suppression in the immunocompetent syngeneic mouse model of EMT6.**

(**A**) Firefly luciferase (*F-Luc*)-labeled EMT6 cells expressing either nonspecific (NS) shRNA or *Ehmt2* shRNA were injected orthotopically into the mammary fat pad of female BALB/c mice ($n = 5$) and treated with either IgG isotype control antibody (100 μg/mouse) or anti-Asialo-GM1 antibody (100 μg/mouse) one day before EMT6 cell line injections and then once a week during the course of the experiment. Bioluminescence images of mice at the indicated weeks after injection are shown. (**B**) Bioluminescence intensities of the mice at the indicated weeks under the indicated conditions for the experiment presented in panel (**A**) are plotted. ($n = 5$). *P* values were calculated using unpaired two-tailed Student's *t*-test. (**C**) Measurement of tumor-infiltrated NK cells (%) in EMT6 tumors expressing either NS shRNA or *Ehmt2* shRNA from BALB/c mice treated with IgG isotype control or anti-Asialo-GM1 antibodies using FACS analysis. ($n = 5$). *P* values were calculated using unpaired two-tailed Student's *t*-test. (**D**) Measurement of relative NK cells (%) in spleens from mice harboring EMT6 tumors expressing either NS shRNA or *Ehmt2* shRNA from mice treated with IgG isotype control or anti-Asialo-GM1 antibodies using FACS analysis. ($n = 5$). *P* values were calculated using unpaired two-tailed Student's *t*-test. All quantitative data were shown as the mean ± SEM.


© The Author(s)

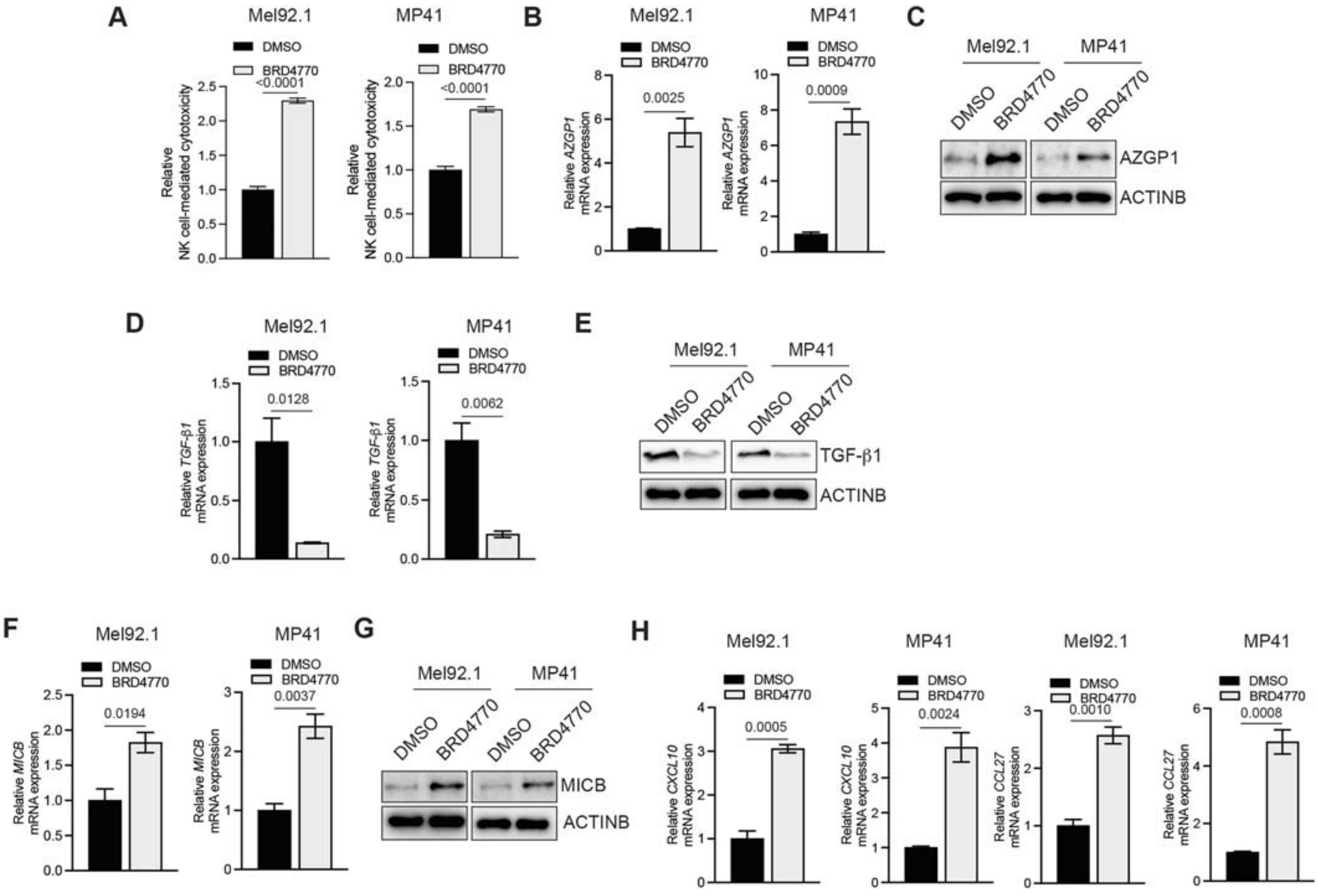


**Figure EV8. The EHMT2 inhibitor BRD4770 enhances NK cell-mediated cytotoxicity, upregulates AZGP1, suppresses TGF-β1, and induces CXCL10 and CCL27 expression.**

(**A**) Mel92.1 and MP41 cells were treated with DMSO or BRD4770 (5 μM) for 48 h and NK cell-mediated cytotoxicity was measured using an LDH-based cytotoxicity assay. Relative NK cell-mediated cytotoxicity under the indicated conditions for indicated UM cell lines is plotted. ($n = 5$). *P* values were calculated using unpaired two-tailed Student's *t*-test. (**B**) Mel92.1 and MP41 cells treated with DMSO or BRD4770 (5 μM) for 48 h and were analyzed for *AZGP1* mRNA using RT-qPCR. mRNA expression relative to DMSO-treated cells is plotted. *ACTINB* was used for normalization. (n = 3). *P* values were calculated using unpaired two-tailed Student's *t*-test. (**C**) Mel92.1 and MP41 cells were treated with DMSO or BRD4770 (5 μM) for 48 h and were analyzed for AZGP1 expression by immunoblotting. ACTINB was used as a loading control. (**D**) Mel92.1 and MP41 cells were treated with DMSO or BRD4770 (5 μM) for 48 h and were analyzed for *TGF-β1* mRNA using RT-qPCR. mRNA expression relative to DMSO-treated cells is plotted. *ACTINB* was used for normalization. ($n = 3$). *P* values were calculated using unpaired two-tailed Student's *t*-test. (**E**) Mel92.1 and MP41 cells were treated with DMSO or BRD4770 (5 μM) for 48 h and were analyzed for TGF-β1 expression by immunoblotting. ACTINB was used as a loading control. (**F**) Mel92.1 and MP41 cells were treated with DMSO or BRD4770 (5 μM) for 48 h and were analyzed for *MICB* mRNA using RT-qPCR. mRNA expression relative to DMSO-treated cells is plotted. *ACTINB* was used for normalization. ($n = 3$). *P* values were calculated using unpaired two-tailed Student's *t*-test. (**G**) Mel92.1 and MP41 cells were treated with DMSO or BRD4770 (5 μM) for 48 h and were analyzed for MICB expression by immunoblotting. ACTINB was used as a loading control. (**H**) Mel92.1 and MP41 cells were treated with DMSO or BRD4770 (5 μM) for 48 h and were analyzed for *CXCL10* and *CCL27* mRNAs using RT-qPCR. mRNA expression relative to DMSO -treated cells is plotted. *ACTINB* was used for normalization. ($n = 3$). *P* values were calculated using unpaired two-tailed Student's *t*-test. All quantitative data were shown as the mean ± SEM.
